# Cost-effectiveness of easy-access, risk-informed oral pre-exposure prophylaxis in HIV epidemics in sub-Saharan Africa: a modelling study

**DOI:** 10.1016/S2352-3018(22)00029-7

**Published:** 2022-04-27

**Authors:** Andrew N Phillips, Anna Bershteyn, Paul Revill, Loveleen Bansi-Matharu, Katharine Kripke, Marie-Claude Boily, Rowan Martin-Hughes, Leigh F Johnson, Zindoga Mukandavire, Lise Jamieson, Gesine Meyer-Rath, Timothy B Hallett, Debra ten Brink, Sherrie L Kelly, Brooke E Nichols, Eran Bendavid, Edinah Mudimu, Isaac Taramusi, Jennifer Smith, Shona Dalal, Rachel Baggaley, Siobhan Crowley, Fern Terris-Prestholt, Peter Godfrey-Faussett, Irene Mukui, Andreas Jahn, Kelsey K Case, Diane Havlir, Maya Petersen, Moses Kamya, Catherine A Koss, Laura B Balzer, Tsitsi Apollo, Thato Chidarikire, John W Mellors, Urvi M Parikh, Catherine Godfrey, Valentina Cambiano, HIV Modelling Consortium

**Affiliations:** a Institute for Global Health, University College London, London, UK; b Department of Population Health, New York University Grossman School of Medicine, New York, NY, USA; c Centre for Health Economics, University of York, York, UK; d Avenir Health, Takoma Park, MD, USA; e Department of Infectious Disease Epidemiology, Imperial College London, London, UK; f MRC Centre for Global Infectious Disease Analysis, School of Public Health, Imperial College London, London, UK; g Burnet Institute, Melbourne, VIC, Australia; h Centre for Infectious Disease Epidemiology and Research, University of Cape Town, Cape Town, South Africa; i Centre for Data Science and Artificial Intelligence, Emirates Aviation University, Dubai, United Arab Emirates; j Health Economics and Epidemiology Research Office (HE2RO), Department of Internal Medicine, School of Clinical Medicine, Faculty of Health Sciences, University of the Witwatersrand, Johannesburg, South Africa; k Department of Medical Microbiology, Amsterdam University Medical Centre, Amsterdam, Netherlands; l Department of Global Health, School of Public Health, Boston University, Boston, MA, USA; m Department of Medicine, Stanford University, Stanford, CA, USA; n Department of Decision Sciences, University of South Africa, Pretoria, South Africa; o National AIDS Council, Harare, Zimbabwe; p World Health Organisation, Geneva, Switzerland; q The Global Fund to Fight AIDS, Tuberculosis and Malaria, Geneva, Switzerland; r Joint UN Programme on HIV/AIDS, Geneva, Switzerland; s London School of Hygiene & Tropical Medicine, London, UK; t Drugs for Neglected Diseases Initiative, Nairobi, Kenya; u Ministry of Health, Lilongwe, Malawi; v Department of Medicine, University of California, San Francisco, CA, USA; w Divisions of Biostatistics and Epidemiology, School of Public Health, University of California, Berkeley, CA, USA; x Department of Medicine, Makerere University, Kampala, Uganda; y Infectious Diseases Research Collaboration, Kampala, Uganda; z Department of Biostatistics and Epidemiology, School of Public Health and Health Sciences, University of Massachusetts, Amherst, MA, USA; aa Ministry of Health and Child Care, Harare, Zimbabwe; ab National Department of Health, Pretoria, South Africa; ac Department of Medicine, Division of Infectious Diseases, University of Pittsburgh, Pittsburgh, PA, USA; ad Office of the Global AIDS Coordinator, Department of State, Washington, DC, USA

## Abstract

**Background:**

Approaches that allow easy access to pre-exposure prophylaxis (PrEP), such as over-the-counter provision at pharmacies, could facilitate risk-informed PrEP use and lead to lower HIV incidence, but their cost-effectiveness is unknown. We aimed to evaluate conditions under which risk-informed PrEP use is cost-effective.

**Methods:**

We applied a mathematical model of HIV transmission to simulate 3000 setting-scenarios reflecting a range of epidemiological characteristics of communities in sub-Saharan Africa. The prevalence of HIV viral load greater than 1000 copies per mL among all adults (HIV positive and negative) varied from 1·1% to 7·4% (90% range). We hypothesised that if PrEP was made easily available without restriction and with education regarding its use, women and men would use PrEP, with sufficient daily adherence, during so-called seasons of risk (ie, periods in which individuals are at risk of acquiring infection). We refer to this as risk-informed PrEP. For each setting-scenario, we considered the situation in mid-2021 and performed a pairwise comparison of the outcomes of two policies: immediate PrEP scale-up and then continuation for 50 years, and no PrEP. We estimated the relationship between epidemic and programme characteristics and cost-effectiveness of PrEP availability to all during seasons of risk. For our base-case analysis, we assumed a 3-monthly PrEP cost of US$29 (drug $11, HIV test $4, and $14 for additional costs necessary to facilitate education and access), a cost-effectiveness threshold of $500 per disability-adjusted life-year (DALY) averted, an annual discount rate of 3%, and a time horizon of 50 years. In sensitivity analyses, we considered a cost-effectiveness threshold of $100 per DALY averted, a discount rate of 7% per annum, the use of PrEP outside of seasons of risk, and reduced uptake of risk-informed PrEP.

**Findings:**

In the context of PrEP scale-up such that 66% (90% range across setting-scenarios 46–81) of HIV-negative people with at least one non-primary condomless sex partner take PrEP in any given period, resulting in 2·6% (0·9–6·0) of all HIV negative adults taking PrEP at any given time, risk-informed PrEP was predicted to reduce HIV incidence by 49% (23–78) over 50 years compared with no PrEP. PrEP was cost-effective in 71% of all setting-scenarios, and cost-effective in 76% of setting-scenarios with prevalence of HIV viral load greater than 1000 copies per mL among all adults higher than 2%. In sensitivity analyses with a $100 per DALY averted cost-effectiveness threshold, a 7% per year discount rate, or with PrEP use that was less well risk-informed than in our base case, PrEP was less likely to be cost-effective, but generally remained cost-effective if the prevalence of HIV viral load greater than 1000 copies per mL among all adults was higher than 3%. In sensitivity analyses based on additional setting-scenarios in which risk-informed PrEP was less extensively used, the HIV incidence reduction was smaller, but the cost-effectiveness of risk-informed PrEP was undiminished.

**Interpretation:**

Under the assumption that making PrEP easily accessible for all adults in sub-Saharan Africa in the context of community education leads to risk-informed use, PrEP is likely to be cost-effective in settings with prevalence of HIV viral load greater than 1000 copies per mL among all adults higher than 2%, suggesting the need for implementation of such approaches, with ongoing evaluation.

**Funding:**

US Agency for International Development, US President's Emergency Plan for AIDS Relief, and Bill & Melinda Gates Foundation.

## Introduction

Oral pre-exposure prophylaxis (PrEP) consisting of emtricitabine (or lamivudine) and tenofovir disoproxil fumarate is highly effective at preventing HIV,^1^ but its use is low in sub-Saharan Africa, the region in which HIV infection rates are highest. WHO recommends PrEP as an “additional prevention choice for people at substantial risk of HIV”, provisionally defined as populations with annual HIV incidence higher than 3%, informed by estimates of what would be considered cost-effective,^2^, ^3^, ^4^, ^5^, ^6^, ^7^ and countries are left to decide policies based on available resources.^8^ PrEP programmes have often seen low levels of continued use.^1^, ^9^, ^10^

**Research in context**

**Evidence before this study**

Oral pre-exposure prophylaxis (PrEP) consisting of emtricitabine (or lamivudine) and tenofovir disoproxil fumarate has high efficacy in preventing HIV acquisition, but there are challenges to developing implementation approaches that realise its full benefits in sub-Saharan Africa. Approaches that allow easy, unrestricted local access to PrEP with education and support might increase risk-informed PrEP use and lead to lower HIV incidence, but the cost-effectiveness of such approaches is uncertain. We searched Web of Science with the search terms “PrEP” or “pre-exposure prophylaxis” AND (list of countries in sub-Saharan Africa) AND (cost-effective*) on April 19, 2021, for articles in English, with no date restrictions. Our search identified 67 articles. Several modelling studies have evaluated PrEP specifically targeted at high-risk groups and generally found it to be cost-effective. Less targeted PrEP has generally been found to not be cost-effective.

**Added value of this study**

We used an individual-based model of HIV to consider the cost-effectiveness of easy-access PrEP for men and women with education to inform use during so-called seasons of risk (ie, periods in which individuals are at risk of acquiring HIV infection). We refer to this as risk-informed PrEP. We find that risk-informed PrEP during seasons of risk is likely to be cost-effective in settings with a prevalence of HIV viral load greater than 1000 copies per mL among all adults of higher than 2%.

**Implications of all the available evidence**

There is a substantive case for the roll-out of easily accessible, over-the-counter PrEP from pharmacies in communities with prevalence of HIV viral load greater than 1000 copies per mL among all adults of higher than 2%. Access will need to be convenient, local, and non-stigmatising and be accompanied by PrEP education to support risk-informed use. Early stages of the roll-out should be monitored to understand actual patterns of PrEP use, to inform real-world cost-effectiveness.

Providing easy and unrestricted access to PrEP drugs (eg, over the counter in local pharmacies), along with community-supported education campaigns to inform when PrEP use would be advised and when not, which we refer to as risk-informed PrEP use, could help to realise the benefits of PrEP. However, the potential of this strategy to be cost-effective is uncertain. An unrestricted PrEP delivery approach was successfully implemented in the Sustainable East Africa Research in Community Health (SEARCH) trial,^11^, ^12^ although this was in the context of a study and still required health system interaction. Modelling in a South African context suggests that risk-targeted PrEP is potentially not only cost-effective, but ultimately cost-saving.^13^, ^14^ In this study, we explore the conditions under which widely accessible PrEP could be cost-effective in sub-Saharan Africa. Our primary analyses assume a concentration of PrEP use during periods of risk with high adherence to daily pill taking, which we hypothesise to be attainable via community-supported programmes.

## Methods

### Model description

Our methods are described in detail in the appendix (pp 1–87) and here we provide a summary. HIV Synthesis is an individual-based simulation model that has been described previously.^14^, ^15^ Each model run generates a simulated population of adults from 1989. Variables that are updated 3-monthly for the entire population, include age, sex, primary and non-primary condomless sex partners, whether currently a female sex worker, HIV testing, male circumcision status, presence of sexually transmitted infections (STIs) other than HIV, and use of oral PrEP. In HIV-positive people, variables include time from infection, CD4 count, viral load, specific antiretroviral drugs being used, antiretroviral adherence, and specific drug-resistance mutations.

3000 so-called setting-scenarios were generated by sampling parameter values to represent a range of settings in sub-Saharan Africa and to incorporate uncertainty in model assumptions (a full list of parameters is provided in the appendix pp 75–85, and a description of characteristics in the resulting setting scenarios is provided in the appendix pp 6–9).

### Policy comparison

For each setting-scenario, we considered the situation in mid-2021 and did a pairwise comparison of the outcomes of two policies: immediate PrEP scale-up and then continuation for 50 years, and no PrEP. We hypothesised that, in the context of easy-access PrEP with education and unrestricted availability, PrEP would be used in a risk-informed way (ie, only during so-called seasons of risk, which consist of one or more 3-month periods in which people have condomless sex with at least one non-primary partner, when a primary condomless partner is known to have HIV but is not on antiretroviral therapy, or when a woman aged <50 years suspects there is a high risk her primary partner might have unsuppressed HIV; implemented as explained in the appendix pp 45–46). In our primary analysis, we assumed that PrEP was not used at other times, but in a sensitivity analysis we considered the effect of also using PrEP during periods when there is no risk. Although we assumed that PrEP would be used during seasons of risk, we do not suggest that programmes restrict use in this way.

Values of parameters determining scale-up, uptake, and continuation of PrEP use during seasons of risk (appendix pp 2–5, 45–46, 75–85) result in a mean of 66% of people who are HIV negative with at least one non-primary condomless sex partner in the current 3 month period being on PrEP at any given point in time (table 1), and high adherence to use of PrEP during periods of risk that we hypothesised to be attainable. We assumed PrEP efficacy of 90–95% if the partner who is HIV positive does not carry virus resistant to both emtricitabine or lamivudine and tenofovir disoproxil fumarate, and efficacy of 50% (25% in sensitivity analysis) when the partner has virus with both Lys65Arg and Met184Val resistance mutations that affect sensitivity to tenofovir and emtricitabine or lamivudine.^16^ Given our parameter values around resistance acquisition, the risk of resistance emergence for people who inadvertently take PrEP while having HIV (appendix pp 2–5, 45–46) is on average 7% with Lys65Arg and 38% with Met184Val, by 3 months of infection.^17^, ^18^, ^19^ To estimate the reduction in HIV incidence in PrEP users due to PrEP, we compared the incidence in PrEP users in the 3000 setting-scenarios with an additional 300 setting-scenarios in which PrEP efficacy was set to zero.

**Table 1:** Characteristics and predicted effects of risk-informed PrEP over 5 years

			**No PrEP**	**Scaled-up PrEP**
Proportion of HIV-negative people on PrEP				
	Adolescent girls and young women (15–24 years)		0%	2·2% (0·9 to 4·2)
	Adult women (15–64 years)		0%	2·8% (1·0 to 5·6)
	Adult men (15–64 years)		0%	2·5% (0·5 to 7·0)
	Sex workers		0%	47% (32 to 64)
Of HIV-negative people with at least one non-primary condomless sex partner, proportion who are on PrEP			0%	66% (46 to 81)
Considering all non-primary condomless sex partnerships had by HIV-negative people, proportion of these for which the person was on PrEP			0%	69% (49 to 85)
Proportion of people aged 15–64 years experiencing a 3-month period in which criteria for risk-informed PrEP were fulfilled				
	In past year		..	9% (4 to 19)
	In past 5 years		..	19% (9 to 33)
Of people on PrEP (in any given 3-month period), proportion who had a non-primary condomless sex partner in the period			..	65% (31 to 91)
Mean number of condomless non-primary partners per 3-month period per non-sex-worker person on PrEP			..	1·9 (1·0 to 3·0)
Of people on PrEP, proportion with ≥80% adherence			..	87% (66 to 95)
Of people on PrEP, proportion with undetected HIV			..	0·9% (0·1 to 2·4)
Proportion of all adults that have HIV and their HIV has specific mutations				
	Met184Val		2·8% (0·1 to 6·0)	2·8% (0·1 to 6·2)
	Lys65Arg		1·3% (0·7 to 1·7)	1·3% (0·8 to 1·7)
HIV incidence in people aged 15–49 years, per 100 person-years				
	Incidence over 5 years		0·51 (0·11 to 1·18)	0·29 (0·05 to 0·71)
	Relative incidence*		..	0·56 (0·34 to 0·81)
HIV incidence in people on PrEP, per 100 person-years†			..	3·2 (0·5 to 8·8)
Proportion of new infections from a non-primary partner			59% (30 to 86)	49% (17 to 82)
HIV programme costs per year (discounted at 3% per year), in millions				
	PrEP cost‡		$0	$21·7 (7·3 to 48·9)
		Difference in cost	..	$21·7 (7·3 to 48·9)
	Cost of HIV treatment and care		$181·47 (61·6 to 343·6)	$181·1 (61·0 to 342·8)
		Difference in cost	..	−$0·3 (−4·7 to 4·1)
	Total programme costs§		$224·9 (88·6 to 405·5)	$248·4 (104·3 to 437·3)
		Difference in cost	..	$23·5 (6·5 to 56·0)
Cost per infection averted¶			..	$1297 (356 to 5719)

For each setting-scenario, the mean was calculated over 3-month periods between mid-2021 and mid-2026, unless otherwise stated. Data are shown as means with 90% ranges, unless otherwise stated; 95% CIs for mean values were within 5% of the mean value in all cases, so they are not shown. Absolute numbers relate to a population containing 10 million adults. All costs are in $US. PrEP=pre-exposure prophylaxis.

* Defined as incidence with PrEP scale-up divided by incidence with no PrEP for each setting-scenario.

† If PrEP efficacy is 0, the mean incidence rate in people on PrEP is 10·8 per 100 person-years.

‡ Not including HIV tests.

§ Costs in addition to PrEP and treatment and care include voluntary medical male circumcision and HIV testing.

¶ Median over setting-scenarios with 90% range; calculated as the mean difference in annual cost divided by the mean annual infections averted.

### Cost-effectiveness analysis

Assumed costs are provided in the appendix (pp 86–87). In our base case, we used a cost of PrEP provision of US$29 per 3 months, consisting of a drug cost of $11 (including supply chain costs, based on the South Africa tender price for PrEP drugs^13^), $4 for an HIV test per 3 months, and $14 per 3 months for additional costs necessary to facilitate education and access. These additional costs depend on the delivery approach; in our primary analysis, we used a cost similar to that used in a cost-effectiveness evaluation in South Africa.^13^ In sensitivity analyses, we considered lower and higher costs.^6^, ^20^

We simulated the absolute numbers of health-related events, costs, and disability-adjusted life years (DALYs) for a base population of 10 million adults in 2021 over a 5-year and 50-year period (a justification of the 50-year time horizon is provided on appendix p 4; a 20-year period is also presented on appendix pp 13–14). Resource use and costs were analysed from a health-care system perspective. We also calculated net DALYs, which are a measure of the health effects of an intervention that encompass the full implications of the intervention being delivered by the health-care system.^21^

In our primary analysis, we used a cost-effectiveness threshold of $500 per DALY averted and a 3% discount rate for both costs and health outcomes to calculate net DALYs averted. In sensitivity analyses, we considered a cost-effectiveness threshold of $100 per DALY averted and a discount rate of 7% per annum. Country-specific thresholds are uncertain but $500 averted per DALY is likely to be at the upper end on the basis of evidence of how resources would otherwise be used*.*^22^

### Role of the funding source

The funder of the study had no role in study design, data collection, data analysis, data interpretation, or writing of the report.

## Results

PrEP use as modelled resulted in a mean of 2·2% (90% range across setting-scenarios 0·9–4·2) of adolescent girls and young women (aged 15–24 years), 47% (32–64) of female sex workers, 2·8% (1·0–5·6) of adult women, and 2·5% (0·5–7·0) of adult men using PrEP at any given time (table 1). The mean proportion of people aged 15–64 years experiencing a 3-month period in the past year in which criteria for risk-informed PrEP were fulfilled was 9% (4–19), and in the past 5 years was 19% (9–33). The mean number of condomless non-primary partners per 3-month period per person (non-sex worker) on PrEP was 1·9 (1·0–3·0). On average over a 5-year period, 87% (66–95) of people on PrEP had greater than 80% pill-taking adherence during the 3-month period.

Over a 5-year time horizon (table 1), this level of PrEP scale-up was estimated to result in 0·56 times (90% range 0·34–0·81) the incidence of HIV without PrEP scale up in people aged 15–49 years (ie, a 44% reduced incidence). The incidence of new HIV infections in people on PrEP was 3·2 per 100 person-years. This compares with 10·8 in a counterfactual situation in which PrEP efficacy was set to zero, suggesting a 70% reduction in incidence in PrEP users due to PrEP. The mean additional annual cost incurred in the context of a country with an adult population of 10 million was $23·5 million per year (6·5 million–56·0 million), resulting in a median cost per infection averted of $1297 (356–5719). Considering the budget impact of PrEP, in the context of a median HIV prevalence in our setting-scenarios of approximately 10%, projected PrEP costs for risk-informed PrEP over 5 years were around 10% of total programme costs (table 1).

Over a 50-year time horizon (2021–71; table 2), the modelled PrEP scale-up across scenarios was estimated to result in 0·51 times (90% range 0·22 to 0·77) the incidence of HIV without PrEP in people aged 15–49 years (ie, a 49% reduced incidence) and 14% (0 to 43) fewer AIDS deaths. Among adolescent girls and young women, the predicted incidence reduction was 54%. PrEP was predicted to result in a lower prevalence of HIV viral load greater than 1000 copies per mL among all adults (HIV positive and negative), from a mean of 2·3% (0·5 to 5·2) over 2021–71 without PrEP to 1·6% (0·3 to 3·7) with PrEP scale-up. Over the 50-year period, the mean annual DALYs averted was 21 600 (–2800 to 58 300) in the context of an adult population of 10 million in 2021, and total annual programme cost savings were $0·9 million (–26·8 million to 23·8 million) compared with no PrEP. The money saved by scaling up PrEP could be used elsewhere to avert 1800 more DALYs per year, based on a cost per DALY averted of $500, meaning a decline in net DALYs of 23 400 (–35 800 to 101 800)*.*

**Table 2:** Predicted effects of risk-informed PrEP programmes over 50 years

			**No PrEP**	**Scaled-up PrEP**
HIV incidence				
	Adults (15–49 years)		0·43 (0·08 to 1·07)	0·24 (0·02 to 0·65)
		Relative incidence*	..	0·51 (0·22 to 0·77)
	Adolescent girls and young women (15–24 years)		0·48 (0·07 to 1·37)	0·23 (0·01 to 0·68)
		Relative incidence*	..	0·46 (0·14 to 0·74)
HIV prevalence in adults aged 15–49 years			6·2% (1·4 to 13·9)	4·2% (0·8 to 9·7)
Prevalence of HIV viral load >1000 copies per mL among all adults (HIV positive and negative)			2·3% (0·5 to 5·2)	1·6% (0·3 to 3·7)
AIDS deaths				
	Number per year over 50 years		15 700 (4200 to 35 300)	13 500 (3300 to 30 800)
	Averted per year		..	2200 (−400 to 6800)
DALYs averted per year over 50 years (mean per year, discounted at 3% per year)			..	21 600 (−2800 to 58 300)
Infections				
	Number per year over 50 years		55 900 (10 500 to 134 500)	32 600 (3000 to 88 900)
	Averted per year over 50 years		..	23 400 (5000 to 54 800)
HIV programme costs per year (discounted at 3% per year), in millions				
	PrEP cost†		$0	$17·9 (5·6 to 40·9)
		Difference in cost	..	$17·9 (5·6 to 40·9)
	Cost of HIV treatment and care		$107·0 (31·0 to 219·0)	$89·1 (25·1 to 184·7)
		Difference in cost	..	−$17·9 (−42·6 to −3·6)
	Total programme costs		$138·9 (48·3 to 267·3)	$138·0 (53·6 to 257·0)
		Difference in cost	..	−$0·9 (−26·8 to 23·8)
Proportion of setting-scenarios in which scaled-up PrEP is cost-saving, %‡			..	51%
Difference in net DALYs (mean per year over 50 years) based on a cost-effectiveness threshold of $500			..	−23 400 (35 800 to −101 800)

For each setting-scenario, the mean was calculated over 3-month periods between mid-2021 and mid-2071, unless otherwise stated. Data are shown as means with 90% ranges, unless otherwise stated; 95% CIs for mean values were within 5% of the mean value in all cases, so they are not shown. Absolute numbers relate to a population containing 10 million adults. All costs are in $US. PrEP=pre-exposure prophylaxis. DALY=disability-adjusted life-year.

* Defined as incidence with PrEP scale-up divided by incidence with no PrEP for each setting-scenario.

† Not including HIV tests.

‡ Cost-saving means that DALYs are averted and costs are saved.

Positive associations were found between the proportion of setting-scenarios in which PrEP is cost-effective and HIV incidence, HIV prevalence, prevalence of HIV viral load greater than 1000 copies per mL among all adults, and the mean number of condomless non-primary partners per 3 months per person with non-primary partners; a negative association was found with the percentage of people aged 15–49 years with one or more non-primary condomless sex partner per 3 months and the proportion of men who are circumcised (table 3). The cost-effectiveness of risk-informed PrEP did not seem to be associated with the proportion of seasons of risk covered by PrEP use over 5 years.

**Table 3:** Percentage of setting-scenarios in which scaled-up PrEP is cost-effective according to baseline characteristics (based on cost-effectiveness threshold of US$500 per DALY averted)

		**Proportion of setting scenarios in which scaled-up PrEP is cost-effective, % (95% CI)**
Overall		71% (70–73)
HIV incidence among people aged 15–49 years in 2021, per 100 person-years		
	<0·2	47% (42–52)
	0·2 to <0·5	64% (61–67)
	0·5 to <1·0	81% (79–84)
	1·0 to <1·5	88% (84–91)
	≥1·5	96% (90–99)
HIV prevalence in people aged 15–49 years in 2021, %		
	0–3%	40% (34–47)
	4–7%	61% (58–64)
	8–11%	73% (70–76)
	12–15%	83% (79–86)
	16–19%	89% (85–92)
	≥20%	91% (86–94)
Of people with HIV, proportion with viral load <1000 copies per mL, %		
	<65%	72% (68–76)
	65–72%	75% (73–78)
	≥73%	67% (65–70)
Prevalence of HIV viral load >1000 copies per mL among all adults (HIV positive and negative) aged 15–64 years, %		
	<1%	35% (26–46)
	1% to <2%	58% (54–62)
	2% to <3%	65% (61–68)
	3% to <5%	78% (75–80)
	≥5%	86% (83–88)
Proportion of men aged 15–49 years who are circumcised, %		
	<33%	82% (78–85)
	33–66%	72% (70–75)
	≥67%	65% (62–67)
Proportion of people aged 15–49 years with one or more non-primary condomless sex partner per 3 months, %		
	<2%	81% (78–83)
	2% to <4%	77% (75–80)
	4% to <6%	65% (60–69)
	≥6%	50% (46–54)
Mean number of condomless non-primary partners per 3 months per non-sex worker with non-primary partners in 2021*		
	<2	49% (46–53)
	2 to <3	78% (75–80)
	≥3	85% (83–87)
Proportion of all non-primary sex partnerships that involve a sex worker, %		
	<50%	71% (68–75)
	50–74%	69% (66–71)
	≥75%	74% (71–77)
Proportion of seasons of risk covered by PrEP use over 5 years, %†		
	<65%	73% (70–76)
	65–79%	71% (68–74)
	≥80%	70% (68–73)
Effective sensitivity of HIV testing, %‡		
	50%	68% (60–75)
	70%	70% (62–77)
	90%	73% (68–77)
	95%	70% (68–72)
	98%	75% (69–81)
	100%	74% (67–80)

PrEP=pre-exposure prophylaxis.

* Not including female sex workers.

† 3-month periods in which risk-informed PrEP criteria fulfilled, but no assumption was made regarding pill-taking adherence during the period.

‡ Sensitivity of the testing approach to identify a positive person; this is a combination of the inherent sensitivity of the test and (in the case of self-testing being available as an option) the probability of the test actually being performed.

Reduced test sensitivity had little effect on the prevalence of people living with HIV among PrEP users (appendix p 15). The prevalence of drug resistance (Lys65Arg and Met184Val) was similar according to HIV test sensitivity (appendix p 16). We did not find a trend of lower cost-effectiveness with lower HIV test sensitivity (table 3). PrEP scale-up was cost-effective in 62% of setting-scenarios if the cost of PrEP was $10 rather than $4 and 74% if the cost was $2 (appendix p 17).

**Figure 1:**
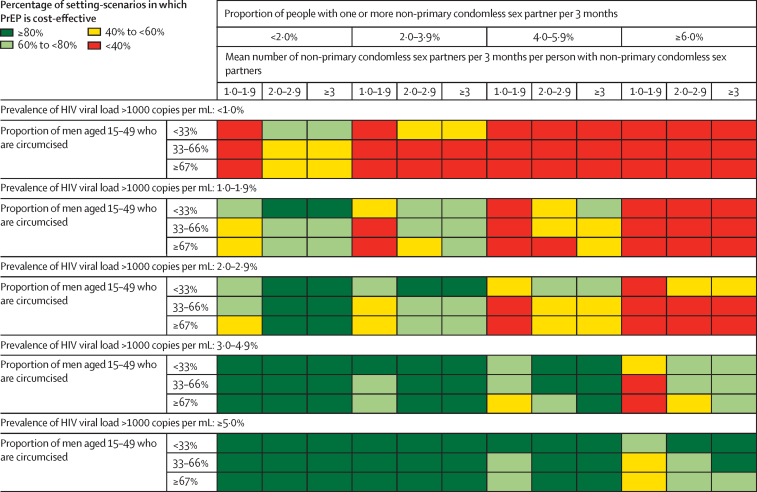
Percentage of setting-scenarios in which PrEP scale-up is predicted to be cost-effective according to key epidemic characteristics Further details are provided in the appendix (p 5). PrEP=pre-exposure prophylaxis.

**Figure 2:**
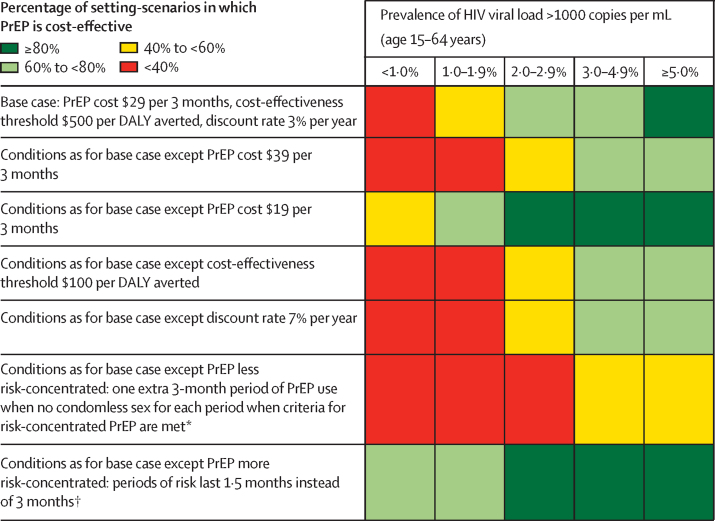
Percentage of setting-scenarios in which PrEP scale-up is predicted to be cost-effective in the base case and various one-way sensitivity analyses Costs are in $US. PrEP cost includes drug costs, visit costs, and HIV testing costs. Results are pooled over the factors in figure 1. DALY=disability-adjusted life-year. PrEP=pre-exposure prophylaxis. *Implemented by doubling the PrEP cost. †Implemented by halving the PrEP cost.

Risk-informed PrEP was likely to be cost-effective in the majority of situations with a prevalence of HIV viral load greater than 1000 copies per mL among all adults of 2% or higher, whereas PrEP was not cost-effective if this prevalence was less than 1% (figure 1). In the 1·0–1·9% range, risk-informed PrEP tended to be cost-effective only if the proportion of people with more than one non-primary condomless sex partner per 3 months was less than around 4%. If using a $100 per DALY averted cost-effectiveness threshold or a 7% per year discount rate, PrEP was only likely to be cost-effective when the prevalence of HIV viral load greater than 1000 copies per mL among all adults was 3% or more (figure 2). If PrEP was used when there was a non-primary condomless partner in the previous 3-month period, even if criteria for risk-informed PrEP were not met in this period, then PrEP was unlikely to be cost-effective. If risk-informed PrEP use was instead concentrated in periods of 1·5 months (rather than 3 months) around the time of condomless sex risk, it was very likely to be cost-effective if the prevalence of HIV viral load greater than 1000 copies per mL among all adults was 1% or more. We also separately considered 300 additional setting-scenarios in which implementation of PrEP was restricted to adolescent girls and young women and sex workers only; this resulted in a mean 37% decline in HIV incidence in adolescent girls and young women over a 50-year period, compared with a 54% decline in adolescent girls and young women in our base case in which PrEP was available to all adults. This approach was cost-effective in 61% of setting scenarios.

We did a sensitivity analysis in which PrEP was assumed to have only 25% efficacy against virus with Lys65Arg and Met184Val mutations. The impact on HIV incidence was reduced (42% compared with 49%) and the percentage of setting-scenarios in which PrEP was cost-effective reduced to 63% compared with 71% in our primary analysis.

In further sensitivity analyses, we considered the effect of changes in parameter values that lowered the uptake of risk-informed PrEP (lower willingness to take PrEP; lower initiation probability in people fulfilling the criteria for risk-informed PrEP; a higher rate of PrEP discontinuation and lower rate of re-initiation; or all of these changes together), each for 300 additional setting-scenarios. These resulted in mean percentages of HIV-negative people with at least one non-primary condomless sex partner being on PrEP of 31% for the lower willingness scenario, 67% for the lower initiation scenario, 55% for the higher discontinuation scenario, and 19% for the combined scenario, compared with 66% in our primary analysis. There was a resulting reduced impact of PrEP on HIV incidence over 50 years of 28%, 43%, 41%, and 17%, respectively, compared with 49% in our primary analysis. However, cost effectiveness of risk-informed PrEP was not diminished compared with our primary analysis: risk-informed PrEP was cost-effective in 69%, 77%, 72%, and 65% of setting-scenarios, respectively, compared with 71% in the primary analysis. We note that this was in the context of a fixed unit cost for PrEP per person on PrEP.

Lastly, we considered a sensitivity analysis in which pill-taking adherence to PrEP was around 50% and nobody had adherence greater than 80% (although PrEP drug cost was assumed to be the same as in our primary analysis). This led to a percentage reduction in incidence over 50 years of 26%, and PrEP was cost-effective in only 31% of setting-scenarios.

## Discussion

Our modelling results suggest that making PrEP easily accessible in the community (eg, over the counter and without charge at pharmacies or through community distribution) for all adults during seasons of risk (ie, risk-informed PrEP) is likely to be cost-effective in communities with prevalence of HIV viral load greater than 1000 copies per mL among all adults (HIV positive and negative) of higher than 2%. Supported by the proof of concept from the SEARCH study that used a similarly inclusive approach,^11^, ^12^ albeit still based on direct engagement with the health-care system, our analysis reinforces the case for roll-out of the approach, which will need to be convenient, local, and non-stigmatising, and supported by PrEP education to facilitate risk-informed use. Such an approach is consistent with the new UNAIDS strategy for 2021–26 and its target that 95% of people at risk of HIV have access to and use appropriate, person-centred, and effective combination prevention options, and that we accelerate PrEP uptake for all people who are at substantial risk of HIV infection, including through simplified and differentiated approaches for delivery.^23^

The choice of settings in which easy PrEP availability will be implemented is likely to be guided by the prevalence of HIV viral load greater than 1000 copies per mL among all adults, with a particularly strong case for setting-scenarios in which this is higher than 2%. This situation presents challenges for national HIV programmes in countries in which there are some settings where easy-access PrEP would likely be cost-effective and others where it might not. We suggest an approach whereby PrEP is available country-wide. There will be some people at high HIV risk in all settings and so ideally PrEP would be accessible for all, initially with surveillance to understand PrEP use and programme costs.

PrEP impact depends on good pill-taking adherence during periods in which an individual intends to be on PrEP. Our assumptions result in a mean of 87% of people on PrEP taking at least 80% of their daily pills. This level of adherence has not been attained by most programmes, but we hypothesise that, although challenging, it is attainable over time as community knowledge and support increases. Results from implementation in rural communities in the SEARCH study are encouraging and show that high pill-taking adherence to PrEP during periods of risk does lead to substantial declines in incidence.^11^, ^12^ In SEARCH, HIV incidence was 74% lower among people on PrEP than in recent matched controls, with a median follow-up of 1·6 years; in our modelling, incidence was 70% lower over 5 years among people on PrEP.

We anticipate that lessons on how to support risk-informed use and pill-taking adherence during periods of use can be drawn from SEARCH and adapted to other contexts. Other studies of improving PrEP knowledge and risk perception are ongoing (eg, NCT03565575).

A potential concern with initiating a roll-out of community-led PrEP programmes is that there will be overuse of PrEP (ie, substantial use by people without an appreciable risk of HIV acquisition), which would not be financially sustainable. We are not aware of evidence thus far for such use, but it is plausible. Monitoring of the proposed approach in a staggered roll-out would be important to assess whether PrEP as implemented will prove to be as cost-effective as predicted. Education is needed around PrEP to encourage its use at all times during periods of risk, but not to encourage it at all times. Another concern might be over the level of initial investment by countries needed to provide large quantities of PrEP in communities to meet possible demand. Although upfront investment would be required, the relatively long shelf life of oral PrEP of 4 years^24^ limits the risk of supplies remaining unused.

We found that attempts to restrict PrEP eligibility to adolescent girls and young women would reduce the benefits to adolescent girls and young women as well as to the community overall, consistent with previous modelling.^25^

Making PrEP easily accessible in the community for all adults to access during seasons of risk will require community buy-in. In some settings, this access might actually be community-led, with the many potential advantages that this approach has for sustained access and support for PrEP users.^26^ There is potential for economies of scope if the community can mobilise and educate beyond PrEP with a wider prevention package. An additional advantage of community-led risk-informed PrEP is that it could promote health self-efficacy and awareness.^27^ Easy access in pharmacies should also make PrEP accessible for sex workers, who can be highly mobile.

Even if ease of PrEP availability to all adults during seasons of risk is achieved along with high levels of PrEP knowledge, there remains a challenge for individuals to be able to adequately anticipate periods of condomless sex with partners of unknown HIV status and hence prepare by taking PrEP. A supportive factor here would be concurrent easy availability of post-exposure prophylaxis (PEP), which has a similar efficacy to PrEP if used within 72 h of exposure (but preferably within 24 h).^28^ Having both PrEP and PEP reliably available locally in communities should enable knowledgeable and motivated community members to provide themselves with a high level of protection during seasons of risk. Easily accessible PEP and emergency contraception could act as a gateway to risk-informed PrEP. Because 4 weeks of PEP use with a regimen such as dolutegravir, tenofovir disoproxil fumarate, and lamivudine is no more costly than 3 months of PrEP, our representation of risk-informed PrEP can be considered as including a component of accompanying PEP use as needed, as long as a course of PEP is taken no more than twice in any 3 month period, given that the daily cost of PEP is approximately 1·5 times that of PrEP.^29^

We acknowledge uncertainty in cost estimates for oral PrEP and HIV testing, especially in future projections. The cost of PrEP drugs might increase or decrease over time, (eg, due to possible introduction of tenofovir alafenamide, which has a lower cost than tenofovir disoproxil fumarate). Delivery costs could also change as evidence accrues regarding new delivery and education approaches and their costs. PrEP programmes in which staff spend time on demand creation and screening for eligibility, as well as conducting STI testing, will result in higher costs compared with an approach in which the drug is available easily, such as over the counter in pharmacies, in the context of community education. Although screening tests for hepatitis and creatinine are suggested for PrEP users, several programmes take the view that this need not be an absolute requirement and that it is sufficient to advise users to consider attending a clinic to get tests for hepatitis and creatinine, and possibly hepatitis B vaccination, especially for those younger than 50 years for whom the risk of renal dysfunction is particularly low. At such a clinic visit, it would probably be appropriate to test for STIs; we did not include any possible benefits from this in our modelling of easy access PrEP. We did not lower our costs for PrEP delivery in recognition of there being less STI, hepatitis, or creatinine testing and for this reason we might have overestimated delivery costs of easy-access PrEP. HIV testing costs would be substantially lower than the $4 we assumed if self-test kits were to be simply distributed from pharmacies. Any costs of ongoing monitoring of PrEP programmes would also need to be factored into the non-drug PrEP costs ($14 in our primary analysis).

In a previous analysis, we found that if PrEP use leads to increases in off-PrEP condomless sex, then PrEP is unlikely to be cost-effective.^14^ Increases in condomless sex with partners of unknown HIV status should be continually assessed and addressed with strengthened messaging on the need to take PrEP as needed to prevent HIV acquisition and improved access to condoms to prevent other STIs to maximise the chance that PrEP achieves its potential as a cost-effective prevention option.

PrEP scale-up is not predicted to lead to any substantial increases in the proportion of the population carrying HIV with resistance mutations to PrEP drugs. We previously showed that the effects on resistance are modest if the first-line antiretroviral regimen is dolutegravir, tenofovir disoproxil fumarate, and lamivudine.^14^ With the expansion of dolutegravir-based antiretroviral therapy, the chance that drug resistance will pose a barrier to effective and cost-effective use of PrEP is reduced. However, the reduced impact and cost-effectiveness of PrEP when we assumed low efficacy against virus with Met184Val and Lys65Arg mutations in a sensitivity analysis illustrates the fact that surveillance of drug resistance remains a crucial activity for countries and would take on an even greater importance if PrEP were available as we propose. The use of oral self-test kits for people on PrEP seems to have no substantive detrimental impact on the prevalence of HIV in people on PrEP and hence also not on population levels of drug resistance to PrEP drugs.

In addition to those discussed already, a further limitation of our modelling is that little reliable data are available on patterns of sexual behaviour, so we aimed to reflect the uncertainty and variability by sampling from a range of parameter values in generating our setting-scenarios. We aimed for our setting-scenarios to reflect the range of epidemic characteristics within subsettings in countries in sub-Saharan Africa. Generating a range of setting-scenarios allowed us to evaluate the effects of epidemic and programme characteristics on PrEP cost-effectiveness, although some aspects are more representative of southern and east Africa rather than central or west Africa, such as the proportion of men who are circumcised. Although we considered the cost-effectiveness of PrEP in the context of shorter periods than 3 months by simply reducing the PrEP cost, we did not explicitly model 1-month periods because of the additional complexity. Also, our modelling concerned only heterosexually transmitted HIV. Furthermore, we note that the comparator for our policy of risk-informed PrEP is no PrEP when, in reality, there is currently already some use of PrEP in sub-Saharan Africa.

In summary, under the assumption that making PrEP easily accessible for all adults in sub-Saharan Africa in the context of community education leads to risk-informed use, PrEP is likely to be cost-effective in settings with prevalence of HIV viral load greater than 1000 copies per mL among all adults higher than 2%, suggesting the need for implementation of such approaches, with ongoing evaluation.

## Data sharing

The model program is available on Figshare (https://figshare.com/articles/software/hiv_synthesis_oral_prep_figshare_sas/19500584).

## Declaration of interests

Unless otherwise stated, all authors are salaried employees of the institutions to which they are affiliated in the header. JWM declares grants from the National Institutes of Health (NIH), US Agency for International Development (USAID), Gilead Sciences, and Janssen Pharmaceuticals Research to the University of Pittsburgh; consulting fees from Gilead Sciences; shares with Abound Bio; and share options with Infectious Diseases Connect. VC reports research grants from UK Research and Innovation (UKRI), Unitaid, National Institute for Health Research, USAID, Medical Research Council (MRC), and Bill & Melinda Gates Foundation; and consulting fees from WHO. TBH declares research grants to their institution from Bill & Melinda Gates Foundation, WHO, UNAIDS, NIH, MRC, and Department for International Development/Foreign, Commonwealth and Development Office; and consulting fees from WHO, Global Fund to Fight AIDS, Tuberculosis and Malaria, and Gilead. ANP declares research grants from UKRI, Wellcome Trust, USAID, NIH, and Bill & Melinda Gates Foundation; and consulting fees from Bill & Melinda Gates Foundation. All other authors declare no competing interests.
