# Supplementary material for: Cost-effectiveness of easy-access, risk-informed oral pre-exposure prophylaxis in HIV epidemics in sub-Saharan Africa: a modelling study
Source: Lancet HIV. 2022 Apr 27;9(5):e353–62. doi: 10.1016/S2352-3018(22)00029-7 (PMC9065367; doi:10.1016/S2352-3018(22)00029-7)
Supplement: Supplementary appendix [file mmc1.pdf]

THE LANCET
HIV

Supplementary appendix

This appendix formed part of the original submission and has been peer reviewed. We post it as supplied by the authors.

Supplement to: Phillips AN, Bershteyn A, Revill P, et al. Cost-effectiveness of easy-access, risk-informed oral pre-exposure prophylaxis in HIV epidemics in sub-Saharan Africa: a modelling study. *Lancet HIV* 2022; **5:** e353–62.

Cost-effectiveness of easy-access, risk-informed oral pre-exposure prophylaxis (PrEP) in HIV epidemics in sub-Saharan Africa

Appendix

1 Extended description of methods and additional results Page 2

2 Model Details Page 18

Extended Description of methods (reference numbers refer to the references in the paper)

HIV Synthesis is an individual based simulation model which has been described previously (e.g., 14,15). Each model run generates a simulated population of adults from 1989 to 2021 and beyond with 3-monthly attributes for the entire population including age, sex, primary and non-primary condomless sex partners (without distinguishing between paid or unpaid; non-primary partnerships are short-term and a person does not need to have a primary partner to have a non-primary partnership), whether currently a female sex worker, HIV testing, male circumcision status, presence of sexually transmitted infections other than HIV, and use of oral PrEP. In HIV-positive people, variables include time from infection, CD4 count, viral load, specific antiretroviral (ARV) drugs being used, ARV adherence, and specific drug resistance mutations. The latter three variables jointly determine at any point in time the levels of suppressive antiviral effect of a regimen.

3000 "setting-scenarios" were generated by sampling several parameter values to represent the range of settings and communities in SSA and to incorporate uncertainty in model assumptions. Sampled parameters include male circumcision rates; propensity to become a sex worker; HIV testing rates; linkage to and retention on ART; ART adherence; resistance emergence, transmission, and persistence; ART interruption; extent of implementation of viral load monitoring; and rate of switching to 2nd line after detected virologic failure (see Table S27 below). Table SR1 below illustrates the characteristics of the range of setting-scenarios, showing diversity in many epidemic aspects, including the extent of sex work, and the extent to which condomless sex among non-sex workers is widespread over individuals or more concentrated. We show comparative national-level estimates where available, however we intend the setting-scenarios to reflect sub-settings within countries as much as countries as a whole. Table SR2 below indicates how these characteristics change over time given the assumptions (in 2041 and 2070).

For each setting-scenario, we consider the situation in mid-2021 and perform a pairwise comparison of outcomes of two policies: immediate PrEP scale up and then continuation for 50 years, and no PrEP. We hypothesize that, in the context of easy-access PrEP with education and unrestricted availability, women and men will use PrEP in a *risk-informed* way; i.e., only during “seasons of risk”, which consist of one or more 3 month periods (the model time step is 3 months) in which they have condomless sex with at least one non-primary partner, when a primary condomless partner is known to have HIV but is not on ART, or when a woman of age < 50 suspects there is a high risk her primary partner might have unsuppressed HIV (implemented as explained in section 6 below). If the individual remains in a “season at risk” in the following 3 months, then PrEP is renewed for an additional 3 months. In our primary analysis we assume that PrEP will not be used at other times, although in sensitivity analysis we consider the effect of also using PrEP during periods when there is no risk. While we assume PrEP will be used during seasons of risk, we do not suggest that programmes restrict use in this way, but rather allow self-selection by users with perceived need.

Each adult in the model for whom the above-mentioned criteria for risk-informed PrEP are met in a given three-month period will only initiate PrEP if they are motivated to do so (the proportion of people willing to initiate PrEP varies between 0.55 and 0.98 across the 3000 setting-scenarios [0.55 to 0.75 in 300 separate setting scenarios as a sensitivity analysis]). Amongst those who are willing to take PrEP, it is only initiated if the individual tests negative for HIV. Since we intend that PrEP will be easily accessible and hypothesize that high uptake is achievable there is then a 90% chance that PrEP is initiated (value of 50% explored in sensitivity analysis of 300 setting scenarios) amongst people willing to take PrEP who test negative. Thereafter, PrEP continuation is accompanied by confirmation of HIV negative status every 3 months. After stopping PrEP due to no longer being at risk , PrEP can be restarted (with 90% probability) if the person re-enters a season of risk and tests HIV negative. People may choose to stop PrEP despite continuing to have new condomless sex partners (5% probability of discontinuation per 3 months), and then, if this is the case, there is assumed to be a 70% chance of resumption per 3 month period of a season of risk (values of 20% and 20%, respectively, explored in sensitivity analysis with 300 separate setting scenarios). If oral HIV self-testing is used to monitor for HIV acquisition in PrEP users, rather than requiring a clinic-based test there is concern that this could lead to a reduced probability of the test being properly performed. We therefore consider in our primary analysis a range of effective test sensitivities focussing primarily on 90% (test sensitivity values of 50%, 70%, 90%, 95% and 100% in 5%, 5%, 75%, 10% and 5% of setting scenarios, respectively). There would be an effective test sensitivity of 50%, for example, if a person only had a 50% chance of doing the HIV test each 3 months while on PrEP.

It is assumed that daily PrEP use during seasons of risk is encouraged, although we account for < 100% pill-taking adherence during 3 month periods of intended use. We assume PrEP efficacy of 90% or 95% (each in 50% of setting-scenarios, reflecting uncertainty) if the HIV-positive partner does not carry virus resistant to both emtricitabine/lamivudine and TDF (1) and that the effectiveness of PrEP in a given 3 month period is the product of efficacy x pill-taking adherence (on a scale of 0 to 1) for that period. PrEP is assumed to have efficacy of 50% (25% in sensitivity analysis) when the partner has virus with both K65R and M184V resistance mutations that affect sensitivity to tenofovir and emtricitabine/lamivudine (16).

Our primary analyses involve high adherence to use of PrEP during periods of risk (87% (90% range over setting scenarios 66% - 95%) of users taking 80% of their daily pills) that we hypothesize to be attainable (we consider a lower adherence in a sensitivity analysis of 300 setting scenarios with adherence 50% and nobody having > 80% adherence). Our assumptions on scale-up, uptake and continuation of PrEP use during seasons of risk result in a mean of 66% of HIV negative people with at least one non-primary condomless sex partner in the current 3 month period being on PrEP at any one given point in time (Table 1). To estimate the reduction in HIV incidence in PrEP users due to PrEP we compare the incidence in PrEP users in the 3000 setting scenarios with a further additional 300 setting-scenarios in which PrEP efficacy is set to zero.

PrEP can inadvertently be used in people living with HIV if they initiate PrEP when already infected with HIV or because they become infected while taking PrEP. Individuals can start PrEP while infected because they are in the primary infection window period, or due to <100% HIV test sensitivity, or due to being in the primary infection window period, assumed to last 3 months. People become infected while taking PrEP because of incomplete adherence, infection with a PrEP drug resistant virus, or less than 100% PrEP efficacy. Given our parameter values around resistance acquisition, the risk of resistance emergence for persons who inadvertently take PrEP while having (drug-sensitive) HIV is on average 7% and 38% by 3 months of infection with K65R and M184V respectively (17, 18, 19). We assume that the rate of voluntary medical male circumcision, (non-PrEP-related) HIV testing, and ART initiation and retention remain constant after mid-2021 and are not influenced by PrEP scale-up.

Costs assumed are given in Table S29 below. In our base case we use a cost of PrEP provision of US$ ($)29 per 3 months, consisting of a drug cost of $11 (including supply chain costs, based on the South Africa tender price for PrEP drugs (13)), $4 for an HIV test per 3 months, and $14 per 3 months for additional costs necessary to facilitate education and access. This latter cost will depend on the delivery approach and in our primary analysis we use a cost similar to that used in a cost-effectiveness evaluation in South Africa (13) while in sensitivity analyses, we considered a higher value of $24, giving a total cost of PrEP provision of $39 per 3 months. We also consider that the cost of enabling access to PrEP could be lower than the $14 in our primary analysis and, further, that drug costs could decline with increased demand, leading us to a total of $19 per 3 months instead of $29 in a further sensitivity analysis. These costs are broadly in line with recent PrEP cost analyses in SSA (6, 20), although community accessed PrEP may be less costly than previous PrEP programme approaches. Nevertheless, we recognise that the costs of delivering easy-access PrEP with the levels of education needed are not yet known and early programmes would need to be evaluated to allow real-life assessment. The intent here is to understand the potential cost-effectiveness of easy-access risk-informed PrEP to give sufficient motivation to it being initiated, in different guises in different places, and better characterized. It is not the intent that this exactly reflects an existing tested intervention. The costs are not incurred in any 3 month period in which PrEP is not used.

We simulate the absolute numbers of health-related events, costs, and disability adjusted life years (DALYs) for a base population of 10 million adults in 2021 over a 5- and 50-year (and 20-year in Appendix) period. The longer time horizons are chosen to allow us to account for the lifetime survival benefits of having avoided HIV during adolescence and young adulthood, and the knock-on effect of reducing further transmissions. For example, for an adolescent girl who takes PrEP, and hence avoids HIV, when without PrEP use she would have become infected there are health and healthcare cost benefits well beyond the next 5 or 20 years. Failure to account for these would mean under-estimation of the cost-effectiveness compared with other interventions. Resource use and cost were analysed from a healthcare system perspective. We also calculate net DALYs averted, which is the difference between DALYs averted by an intervention and DALYs that could have been

averted with any additional health care system resources required to implement it, or, if the intervention saves health care system costs, it is the DALYs averted by the intervention plus the DALYs that can also be averted with the cost savings offered (21). In other words, net DALYs is a measure of the health effects of an intervention that encompasses the full implications of the intervention being delivered by the health care system.

In our primary analysis we use a cost-effectiveness threshold of US$500 per DALY averted and a 3% discount rate for both costs and health outcomes to calculate net DALYs averted while, in sensitivity analyses, we consider a cost-effectiveness threshold of US$100 per DALY averted and a discount rate of 7% per annum. Country-specific thresholds are uncertain but US$500 averted per DALY is likely to be at the upper end on the basis of evidence concerning how resources would otherwise be used (22).

Supplementary information for Figure 1 (in the paper).

Figure 1 shows the percentage of settings-scenarios in which PrEP scale-up is predicted to be cost-effective according to key epidemic characteristics. This percentage is the predicted probability of being cost-effective based on a logistic model fitted over setting scenarios, calculated as described below.

Log_e odds of PrEP being cost effective =

-0.53

+1.24 if prevalence of HIV VL > 1000 copies/mL 1% - 2%
+1.77 if prevalence of HIV VL > 1000 copies/mL 2% - 3%
+3.03 if prevalence of HIV VL > 1000 copies/mL < 3% - 5%
+3.90 if prevalence of HIV VL > 1000 copies/mL $\geq$ 5%

+0.99 if mean number of non-primary condomless sex partners per 3 months per person with non-primary condomless sex partners 2-3
+1.21 if if mean number of non-primary condomless sex partners per 3 months per person with non-primary condomless sex partners $\geq$ 3

-0.57 if proportion of people with one or more non-primary condomless sex partner per 3 months 2% - 3.9%
-1.43 if proportion of people with one or more non-primary condomless sex partner per 3 months 4% - 5.9
-2.41 if proportion of people with one or more non-primary condomless sex partner per 3 months $\geq$ 6%

-0.62 if proportion of men aged 15-49 who are circumcised 33% - 66%
-0.83 if proportion of men aged 15-49 who are circumcised $\geq$ 67%

Estimated percentage of setting scenarios in which PrEP cost effective = (exp(ln odds) / (1 + exp(ln odds)) x 100%

Table SR1. Description of setting-scenarios in 2017 and 2021. Based on n = 3000 setting-scenarios.

**Characteristic**	**Model** (median, 90% range)<br>2017	 2021	**Examples of observed data***
HIV prevalence			
Women/men (age 15-49)	13.2% (5.0% - 27.8%) / 8.6% (3.2% - 17.9%)	11.6% (4.1% - 25.3%) / 7.5% (2.7% - 16.2%)	Zimbabwe 2016 16%/11%% 2020 15%/9%, U Rep Tanzania 2017 6%/3%, Uganda 2017 8%/4%, Lesotho 2017 30%/19%, Eswatini 2017 34%/19%, Malawi 2016 12%/8%, Namibia 2017 15%/8%, Zambia 206 14%/8%, Cameroon 2017 5%/2% 2018 3%/2%**, Cote d'Ivoire 2017/18 4%/1%
Women / men (age 15-24)	2.9% (0.7% - 9.7%) / 0.8% (0.1% - 3.0%)	2.1% (0.5% - 7.6%) / 0.6% (0.1% - 2.3%)	Malawi 2016 3.4% / 1.5%, Zimbabwe 2016 6%/3% 2020 5%/2%, U Rep Tanzania 2.1% / 0.6%, Uganda 3.1% / 0.6%, Eswatini 13.9% / 4.1%, Namibia 5.4% / 2.5%, Cote d'Ivoire 1.0% / 0.3%
Female sex workers	65% (35% - 84%)	60% (30% - 81%)	Zimbabwe 2013 58% (1)
HIV incidence (/100 person years) Women / men (age 15-49)	0.74 (0.20 – 1.91) / 0.54 (0.15 – 1.31)	0.56 (0.13 – 1.70) / 0.41 (0.10 – 1.16)	Malawi 2016 0.44/0.22, Zambia 2016 1.00/0.28, Zimbabwe 2016 0.57/0.30 2020 0.67/0.23, Lesotho 2017 1.31/1.05, Namibia 2016 0.66/0.15, Eswatini 2017 1.73/0.85, Tanzania 2017 0.34/0.14, Cameroon 2017 0.40/0.08
Proportion of HIV positive people age 15+ diagnosed (women / men)	89% (81% - 96%) / 77% (64% - 90%)	92% (85% - 97%) / 82% (69% - 93%)	[&&] Malawi 2016 80%/72%, Zambia 2016 73%/69%, Zimbabwe 2016 80%/72% 2020 88%/84%, Namibia 2017 83%/71%, Tanzania 2017 65%/52%, Ethiopia 2018 83%/70%, Cote d'Ivoire 2017/18 43%/24%, Cameroon 2017 58%/51%
Proportion of diagnosed HIV positive people on ART (women / men)	91% (81% - 97%) / 88% (73% - 95%)	94% (84% - 98%) / 90% (76% - 97%)	Lesotho 2016/17 92%/92%, South Africa 2017 71%, Eswatini 2016/17 88%/90%, Namibia 2017 96%/94%, Zambia 2016 87%/88%, Tanzania 2016/17 95%/90%, Ethiopia 96%/99%, Malawi 2016 93%/89%, Uganda 2016/17 90%/85%, Cameroon 2017 93%/94%, Zimbabwe 2016 89%/88% 2020 98%/96%, Cote d'Ivoire 2017/18 93%/71%.

Proportion of all HIV positive people with VL < 1000 copies/mL	64% (49% - 75%)	72% (57% - 82%)	Zambia 2016 59%, Malawi 2016 68%, Zimbabwe 2016 60% 2020 76%, Eswatini 2017 73%, Lesotho 2017 68%, Tanzania 2017 52%, Uganda 2017 60%, Namibia 2017 77%, Ethiopia 2018 70%, Cote d'Ivoire 2017/18 40%, Cameroon 2017 47%.
Prevalence of HIV viral load > 1000 copies/mL amongst all adults (HIV positive and negative) (aged 15-64)	4.3% (1.6% - 9.2%)	3.2% (1.1% - 7.4%)	Zambia 2016 4.8% (age 15-59), Namibia 2017 2.8% (age 15-64), Malawi 2015/16 3.4% (age 15-64), Zimbabwe 2016 5.7% (age 15-64) 2020 3.1% (age 15+), Cote d'Ivoire 2018 1.7% (age 15-64), Eswatini 2017 7.3% (age 15+), Lesotho 2018 8.3% (age 15-59).
Proportion of ART experienced people who have started 2nd-line ART	5% (2% - 12%)	10% (3% - 24%)	Malawi ~3%% (Malawi MoH Quarterly Reports)<br>Zimbabwe 5% 2018 (Review of Zimbabwe HIV Programmes)
Of people on ART, proportion with VL < 1000. Women / men (age 15-64)	89% (76% - 95%) / 85% (69% - 92%)	92% (80% - 96%) / 88% (72% - 94%)	Zambia 2016 90%/88%, Malawi 2016 92%/90%, Zimbabwe 2016 88%/84% 2020 91%/89%, Namibia 2017 90%/92%, Tanzania 2017 83%/89%, Ethiopia 2018 87%/95%, Cote d'Ivoire 2017/18 78%/65%, Cameroon 2017 80%/81%.
Of people on ART, percent with CD4 < 500	53% (47% - 62%)	48% (43% - 56%)	Eswatini 40% 2016/17, Malawi 52% 2016, Tanzania 55% 2017, Zambia 59% 2016.
Of ART naïve ART initiators, % with NNRTI mutation / M184V / K65R	11% (3% - 24%) / 2% (0% - 5%) / 1% (0% - 3%)	19% (6% - 38%) / 3% (0% - 9%) / 2% (0% - 6%)	For NNRTI: Angola 2012 14%, Botswana 2016 8% , South Africa 2017 14% , Zimbabwe 2015 10%, Namibia 9%, Uganda 2016 16%, Cameroon 8%. (WHO HIV drug resistance report 2017) (2)
Percent of adults (women / men) age 15-49 with at least one non-primary (/ non-co-habiting) condomless partner in past 3 months ++	3.0% (0.9% - 9.0%) / 3.5% (0.9% - 13.7%)	2.4% (0.7% - 8.5%) / 3.1% (0.7% - 12.7%)	Malawi 2015/16 DHS of women age 15-49 having sexual intercourse in past year, 10% women report sex with a non-marital, non-co-habiting partner – of these 50% reported using a condom; of men having sexual intercourse in past year, 26% men report sex with a non-marital, non-co-habiting partner – of these 75% reported using a condom.<br>Zimbabwe 2015 DHS of women age 15-49 having sexual intercourse in past year, 14% women report sex with a non-marital, non-co-habiting partner – of these 67% reported using a condom; of men having sexual intercourse in past year, 37% men report sex with a non-marital, non-co-habiting partner – of these 85% reported using a condom.

			Sexual partnerships tend to be substantially under-reported, especially by women. (e.g. 3-5) &&
Mean number of non-primary partners per 3 months per person with non-primary partners &	2.7 (1.6 – 3.9)	2.7 (1.6 – 4.0)	No reliable data known to be available && Lesotho ~ 3.4, Cote d'Ivoire 3.7, Kenya 3.0, 2.8 Uganda, 3.8 Zambia, for partners in past year. (6)
Percentage of people on PrEP (before the PrEP scale-up in 2021)<br>Women age 15-24<br>Female sex workers	 0.0% (0.0% - 0.0%)<br>0.0% (0.0% - 0.0%)	 0.3% (0.1% - 1.0%)<br>8% (1% - 24%)	 -------<br>e.g. Senegal 60% (7) Kenya 31% 2018 (8)
Percentage of new infections for which the source is a non-primary partner	58% (28% - 86%)	59% (27% - 87%)	No reliable data known to be available.
Percentage of women aged 15-49 who are sex workers	0.9% (0.3% - 2.8%)	0.9% (0.3% - 2.8%)	1-4% Vandepitte et al, 1.2% Fearon et al. (9, 10)
Proportion of all non-primary sex partnerships which involve a sex worker	65% (26% - 91%)	68% (28% - 92%)	&& No data to directly inform, wide bounds reflect uncertainty.
Percentage of men aged 15-49 who are circumcised	39% (12% - 92%)	58% (15% - 94%)	Eswatini 2017 27.6 Lesotho 2017 67.6 Malawi 2016 25.3 Namibia 2016 38.5 South Africa 2017 61.6 Tanzania 2017 77.6 Uganda 2016 42.2 Zambia 2016 27.1 Zimbabwe 2016 14.1
AIDS death rate in people with HIV (per 100 person years)	1.4 (0.7 – 2.9)	1.3 (0.6 – 2.7)	UNAIDS estimates 2020: Malawi 1.1, Mozambique 2.0, Uganda 1.2, South Africa 0.9, Rwanda 1.1, Zimbabwe 1.3, Cote d'Ivoire 2.9, Ghana 3.4 (11)

[*] all observed data from PHIA surveys unless stated (https://phia.icap.columbia.edu/) (12) ; [&]not including female sex workers [++] includes female sex workers. [**] DHS [&&] both HIV diagnosis and, to a substantial extent, sexual partnerships (especially in reporting by women) tend to be under-estimated by self report (e.g., 3-5).

References for Table SR1

1. Cowan FM, Davey C, Fearon E, Mushati P, Dirawo J, Chabata S et al. Targeted combination prevention to support female sex workers in Zimbabwe accessing and adhering to antiretrovirals for treatment and prevention of HIV (SAPPH-IRe): a cluster-randomised trial. Lancet HIV 2018; 5: e417–26
2. HIV drug resistance report 2017. Geneva: World Health Organization; 2017. Licence: CC BY-NC-SA 3.0 IGO. http://www.who.int/hiv/pub/drugresistance/hivdr-report-2017/en
3. Desmond N, Nagelkerke N, Lora W, Chipeta E, Sambo M, Kumwenda M, et al. Measuring sexual behaviour in Malawi: a triangulation of three data collection instruments. BMC Public Health (2018) 18:807
4. Glynn JR, Kayuni N, Banda E, Parrott F, Floyd S, et al. (2011) Assessing the Validity of Sexual Behaviour Reports in a Whole Population Survey in Rural Malawi. PLoS ONE 6(7): e22840. doi:10.1371/journal.pone.0022840
5. Yeatman S, Trinitapoli J. Best-Friend Reports: A Tool for Measuring the Prevalence of Sensitive Behaviors. Am J Public Health. 2011;101:1666–1667. doi:10.2105/AJPH.2011.300194
6. Carael M, Cleland J, Deheneffe JC, Ferry B, Ingham R. Sexual behaviour in developing countries: implications for HIV control. AIDS 1995; 9:1171-1175
7. Sarr M, Gueye D, Mboup A, Diouf O, Bousso Bao MD, Ndiaye AJ et al. Uptake, retention, and outcomes in a demonstration project of pre-exposure prophylaxis among female sex workers in public health centers in Senegal. International Journal of STD & AIDS 2020, Vol. 31(11) 1063–1072
8. OPTIONS MARKET INTELLIGENCE REPORT: KENYA JULY 31, 2018 Key insights and communications implications for oral PrEP demand creation among female sex workers https://www.prepwatch.org/wp-content/uploads/2018/08/OPTIONS_FSW_Kenya_July2018.pdf
9. Vandepitte J, Lyerla J, Dallabetta G, Crabbe F, Alary M, Buve A. Estimates of the number of female sex workers in different regions of the world. Sex Transm Infect 2006;82(Suppl III):iii18–iii25. doi: 10.1136/sti.2006.020081
10. Fearon E, Chabata ST, Magutshwa S, Ndori-Mharadze T, Musemburi S, Chidawanyika H, et al. Estimating the Population Size of Female Sex Workers in Zimbabwe: Comparison of Estimates Obtained Using Different Methods in Twenty Sites and Development of a National-Level Estimate. J Acquir Immune Defic Syndr 2020;85:30–38
11. https://www.unaids.org/en/resources/documents/2020/unaids-data
12. Population Based HIV Impact Assessment. https://phia.icap.columbia.edu/

Table SR2. Description of predicted characteristics in the specific years 2021, 2041 and 2070 with and without PrEP scale-up. Mean (90% range over setting scenarios)

**Characteristic**	2021	2041		2070	
		No PrEP	Scaled up PrEP	No PrEP	Scaled up PrEP
HIV prevalence<br>Women/men (age 15-49)	12.8% (4.1% - 25.3%) / 8.2% (2.7% - 16.2%)	7.1% (1.54 - 17.1%) / 4.2% (0.8% - 10.1%)	4.3% (0.7% - 10.9%) / 2.6% (0.3% - 6.8%)	6.9% (1.1% - 17.4%) / 4.3% (0.7% - 11.1%)	3.5% (0.0% - 10.0%) / 2.5% (0.0% - 7.0%)
HIV incidence (/100 person years) Women / men (age 15-49)	0.69 (0.13 – 1.70) / 0.49 (0.10 – 1.16)	0.47 (0.06 – 1.26) / 0.34 (0.05 – 0.88)	0.23 (0.01 – 0.66) / 0.20 (0.01 – 0.57)	0.49 (0.05 – 1.37) / 0.35 (0.03 – 0.93)	0.26 (0.00 – 0.80) / 0.21 (0.00 – 0.65)
Proportion of HIV positive people age 15+ diagnosed (women / men)	92% (85% - 97%) / 82% (69% - 93%)	93% (87% - 98%) / 83% (71% - 93%)	96% (92% - 99%) / 89% (80% - 96%)	92% (85% - 97%) / 80% (69% - 92%)	94% (89% - 100%) / 83% (73% - 100%)
Proportion of diagnosed HIV+ people on ART (women / men)	93% (84% - 98%) / 89% (76% - 97%)	93% (86% - 98%) / 91% (81% - 97%)	93% (86% - 98%) / 91% (82% - 97%)	93% (85% - 98%) / 90% (79% - 97%)	92% (85% - 98%) / 89% (77% - 97%)
Proportion of all HIV positive people with VL < 1000 copes/mL	71% (57% - 82%)	76% (64% - 85%)	78% (66% - 88%)	75% (62% - 84%)	72% (57% - 88%)
Of adult population, proportion with viral load > 1000 copies/mL	3.6% (1.1% - 7.4%)	2.2% (0.5% - 5.1%)	1.5% (0.3% - 3.5%)	1.9% (0.3% - 4.9%)	1.2% (0.0% - 3.5%)
Proportion of ART experienced people who have started 2nd-line ART	11% (3% - 24%)	10% (2% - 23%)	13% (2% - 27%)	6% (0% - 16%)	13% (0% - 29%)
Of people on ART, proportion with VL < 1000. Women / men (age 15-64)	91% (80% - 96%) / 86% (72% - 94%)	94% (87% - 98%) / 89% (79% - 96%)	93% (84% - 97%) / 87% (75% - 95%)	95% (88% - 99%) / 90% (79% - 97%)	90% (79% - 97%) / 85% (69% - 95%)

Of people on ART, percent with CD4 < 500	48% (43% - 56%)	47% (42% - 52%)	49% (45% - 55%)	46% (41% - 53%)	52% (45% - 60%)
Of ART naïve ART initiators % with M184V / K65R	4% (0% - 9%) /<br>2% (0% - 6%)	3% (0% - 8%) /<br>2% (0% - 5%)	15% (0% - 33%) /<br>11% (0% - 28%)	2% (0% - 6%) /<br>1% (0% - 4%)	18% (0% - 37%) /<br>16% (0% - 36%)
Percent of adults (women / men) age 15-49 with at least one non-primary condomless partner in past 3 months	3.2% (0.7% - 8.5%) /<br>4.5% (0.7% - 12.7%)	2.8% (0.6% - 7.3%) /<br>4.2% (0.6% - 12.1%)	2.8% (0.6% - 7.4%) /<br>4.1% (0.6% - 12.1%)	2.8% (0.5% - 7.1%) /<br>4.2% (0.5% - 13.0%)	2.8% (0.5% - 7.1%) /<br>4.1% (0.5% - 12.4%)
Mean number of non-primary partners per 3 months per person with non-primary partners	2.7 (1.6 – 4.0)	2.7 (1.6 – 4.0)	2.6 (1.6 – 4.0)	2.7 (1.6 – 4.1)	2.7 (1.6 – 4.1)
Percentage of HIV negative people aged 15-64 on PrEP<br><br>Women age 15-24<br><br>Female sex workers	 <br><br>0.4% (0.1% - 1.0%)<br><br>9% (1% - 24%)	 <br><br>0.0% (0.0% - 0.0%)<br><br>0% (0% - 0%)	 <br><br>2.3% (0.9% - 4.5%)<br><br>49% (32% - 66%)	 <br><br>0.0% (0.0% - 0.0%)<br><br>0% (0% - 0%)	 <br><br>2.8% (1.0% - 6.0%)<br><br>48% (30% - 66%)
Percentage of new infections from a non-primary partner	58% (27% - 87%)	65% (33% - 91%)	61% (7% - 93%)	66% (36% - 91%)	68% (32% - 93%)
Percentage of women aged 15-49 who are sex workers	1.2% (0.3% - 2.8%)	1.1% (0.3% - 2.7%)	1.1% (0.3% - 2.7%)	1.1% (0.2% - 2.7%)	1.1% (0.2% - 2.7%)
Proportion of all non-primary sex partnerships which involve a sex worker	65% (28% - 92%)	68% (37% - 92%)	68% (37% - 93%)	66% (36% - 91%)	66% (35% - 90%)
Percentage of men aged 15-49 who are circumcised	56% (15% - 94%)	7% (26% - 97%)	72% (28% - 97%)	71% (15% - 97%)	71% (19% - 97%)

HIV death rate in people with HIV (per 100 person years)	1.4 (0.6 – 2.7)	1.3 (0.6 – 2.2)	1.5 (0.7 – 2.6)	1.2 (0.5 – 2.1)	1.7 (0.7 – 3.0)

Table SR3. Predicted effects of risk-informed PrEP programmes over a **20 year** time horizon.

**Characteristic of PrEP programme** (For each setting scenario we take the mean over 3 month periods 2021.5-2041.5)	**No PrEP** (Mean over setting scenarios; 90% range**)	**Scaled up PrEP**
HIV incidence<br><br>Adults age 15-49<br>Relative incidence^^	 <br>0.46 (0.09 -1.11)<br>----	 <br>0.25 (0.04 -0.66)<br>0.53 (0.30 – 0.78)
HIV prevalence in adults age 15-49	7.5% (2.1%-16.0%)	6.2% (1.6%-13.1%)
Number of AIDS deaths per year over 20 years +<br><br>AIDS deaths averted per year ^^^	15,300 (4,600-32,500)<br><br>----	14,200 (4,200-30,700)<br><br>1,100 (-300 – 3,400)
DALYs averted per year over 20 years (mean per year, discounted at 3% per year)	----	11,400<br>(9,700-36,300)
Number of infections per year over 20 years<br><br>Infections averted per year over 20 years	46,900 (10,900-107,200)<br><br>---	27,000 (4,600-66,800)<br><br>19,800 (4,000-46,100)
HIV programme costs per year (mean $m, discounted at 3% per year) and difference ^^^<br><br>PrEP cost ~<br><br>Cost of HIV treatment and care<br><br>Total programme costs	 <br><br>$0<br>----<br>$150.5 (48.4-293.0)<br>---<br>$190.1m (70.4-352.0)<br>----	 <br><br>$21.0m (7.0-46.9)<br>+$21.0m (7.0-46.9)<br>$139.7m (44.6-271.7)<br>-$10.8m (-26.2- -1.5)<br>$200.9m (82.4-360.0)<br>+$10.9m (-7.8- +37.0)
Incremental cost effectiveness ratio	----	Median over setting scenarios $1051 (90% range: cost saving – DALYs not averted)<br><br>Cost effective in 37% of setting scenarios (using threshold of $500 per DALY averted)

Difference in Net DALYs (mean per year over 20 years) based on a CET of $500	----	+10,300 (+66,400 - -38,600)

* For each setting scenario we take the mean over 3 month periods 2021.5-2041.5. + Absolute numbers relate to a population containing 10 million adults; ** 90% range over setting scenarios; ^^ defined as incidence with PrEP scale up divided by incidence with no PrEP for each setting scenario; ^^^ defined as difference with no PrEP compared with PrEP scale up for each setting scenario; " mean differenece in annual cost / mean annual infections averted; [&] not including female sex workers; [~] except for HIV tests.

Table SR4. Of people on PrEP, percent with (undetected) HIV according to sensitivity of HIV testing (mean over 5 years 2021.5-2026.5).

	Percent (90% uncertainty range)
Overall	0.9% (0.1%-2.4%)
Effective sensitivity of HIV testing^^	
50%	1.3% (0.2%-3.5%)
70%	1.0% (0.2%-2.5%)
90%	0.9% (0.1%-2.2%)
95%	0.9% (0.1%-2.4%)
98%	0.9% (0.1%-2.4%)
100%	0.8% (0.1%-2.0%)

^^ Sensitivity of the testing approach to identify a positive person – this is a combination of the inherent sensitivity of the test and (in the case of self-testing being available as an option) the probability of the test actually being performed.

Table SR5. Proportion of all adults that have HIV and their HIV has M184V / K65R mutations, according to HIV test sensitivity (mean over 5 years 2021.5-2026.5)

	Percent (90% uncertainty range)	
	No PrEP	Scaled up PrEP
Overall	2.8% / 1.5%	2.9% / 1.6%
Effective sensitivity of HIV testing^^		
50%	2.9% / 1.6%	3.0% / 1.6%
70%	2.9% / 1.6%	3.0% / 1.7%
90%	2.8% / 1.6%	2.9% / 1.6%
95%	2.8% / 1.5%	2.8% / 1.6%
98%	2.8% / 1.6%	2.9% / 1.6%
100%	2.7% / 1.5%	2.8% / 1.5%

^^ Sensitivity of the testing approach to identify a positive person – this is a combination of the inherent sensitivity of the test and (in the case of self-testing being available as an option) the probability of the test actually being performed.

Table SR6. Cost-effectiveness of risk informed PrEP according to sensitivity of HIV testing and unit cost of testing.

	% of setting-scenarios in which scaled up PrEP is cost-effective (based on cost-effectiveness threshold $500 per DALY averted) (95% confidence interval)
**Test cost $4**	
Overall	71% (70%-73%)
Effective sensitivity of HIV testing	
50%	68% (60%-75%)
70%	70% (62%-77%)
90%	73% (68%-77%)
95%	70% (68%-72%)
98%	75% (69%-81%)
100%	74% (67%-80%)
**Test cost $10**	
Overall	62% (60%-64%)
Effective sensitivity of HIV testing	
50%	54% (45%-62%)
70%	61% (52%-69%)
90%	62% (57%-66%)
95%	62% (60%-64%)
98%	65% (59%-71%)
100%	66% (59%-73%)
**Test cost $2**	
Overall	74% (72%-75%)
Effective sensitivity of HIV testing	
50%	72% (65%-79%)
70%	72% (64%-79%)
90%	76% (71%-80%)
95%	73% (71%-75%)
98%	77% (71%-82%)
100%	77% (70%-83%)

Model details

Contents

1 Introduction to the approach taken 20
2 Demographic model 20
2.1 General population death rates and determination of age in 1989 20
2.2 Pregnancy and parity 21
3 Sexual behaviour and risk of HIV acquisition 23
3.1 Determination of number of short term (condomless sex) partners at period t 23
3.2 Determination of having a long term (condomless sex) partner at period t 34
3.2.1 Starting a new long term condomless partnership at period t 34
3.2.2 Stopping a long term condomless partnership at period t 35
3.3 Determination of number of short term (condomless sex) partners who are HIV infected at time t 36
3.4 Determination of probability that a long term partner is HIV infected at time t 38
3.5 Determination of the risk of infection from a short term partner to the subject 39
3.6 Determination of the risk of infection from a long term partner to the subject 41
3.7 Determination of the risk of infection from the subject to a long term partner 41
4 Transmitted resistance 42
4.1 Transmitted resistance: details 43
4.2 Loss from majority virus of transmitted mutations 44
5 People being hard to reach for services 44
6 Oral PrEP 45
6.1 Overview of modelling of oral PrEP 45
7 Male Circumcision 46
8 HIV progression in absence of treatment 46
8.1 Determination of changes in viral load and CD4 count 47
9 HIV testing and diagnosis of HIV infection 48
10 Modelling the effect of ART 48
10.1 Modelling the effect of ART 49
10.2 Initiation of ART 50
10.3 Switch to second line after failure of first line ART 51
10.4 Adherence pattern 51
10.5 Effect of current drug toxicity and current TB or WHO stage 4 condition on adherence 54
10.6 Effect of age and gender on adherence 54

10.7 Effective adherence 58
10.8 Effect of viral load measurement above 1000 cps/mL on adherence 58
10.9 ART interruption / discontinuation 59
10.10 Interruption of ART without clinic/clinician being aware 60
10.11 Re-initiation of ART after interrupting in patients still under clinic follow-up 60
10.12 Interruption due to drug stock-outs 60
10.13 Loss to follow-up while off ART (for reasons apart from drug stock-outs) 60
10.14 Effect of ART on viral load, CD4 count, resistance development and drug toxicity 61
10.15 Number of active drugs 62
10.16 Classification of adherence levels 62
10.17 Determination of viral load, CD4 count and risk of resistance in people on ART 62
10.18 Variable patient-specific tendency for CD4 count rise on ART 64
10.19 Accelerated rate of CD4 count loss if PI not present in regimen 65
10.20 Variability in individual (underlying) CD4 counts for people on ART 65
10.21 Viral load and CD4 count changes during ART interruption 69
10.22 Incidence of new current toxicity and continuation of existing toxicity 69
10.23 Switching of drugs due to toxicity 70
10.24 Emergence of specific resistance mutations and their effect on drug activity 70
10.25 New resistance to NNRTI arising as a result of ART interruption 71
10.26 Loss of acquired mutations from majority virus 71
10.27 Determination of level of resistance to each drug 72
10.28 Calculation of activity level of each drug 73
10.29 Risk of clinical disease and death in HIV infected people 73
11 Distributions for parameters 74
12 Disability weights and costs 86
References 88

1 Introduction to the approach taken

The model is an individual-based stochastic simulation model including sexual behaviour, HIV transmission, HIV progression and effects of ART within a sub-Saharan African context (1-3). Being "individual-based" means that each time the model is run it generates a data set of the simulated lifetime adult experience of a population of people thought to reflect a setting (e.g. a country, district, town, or other small area). For each simulated person in the data set, there are variables such as age and condomless sex, male circumcision, oral PrEP use, HIV testing, and, if infected with HIV, on HIV diagnosis, CD4 count, viral load, use of specific ART drugs, adherence, resistance. Below we describe full details.

We apply the model by considering a series of "setting scenarios", each generated by one run of the model by sampling several parameter values to reflect the range of settings in SSA and also to incorporate uncertainty in assumptions. The sampled parameters, which are described in full below in Table S27, include male circumcision rates; initiation of sex work; HIV testing; linkage and retention; ART adherence; resistance emergence, transmission and persistence; ART interruption; extent of implementation of viral load monitoring; rate of switching to 2nd line after detected virologic failure.

2 Demographic model

The model runs from 1989 (assumed to be the start of the epidemic) with variables updated in 3 month periods. Each run of the simulation program creates 100,000 simulated people who will be age 15 or above at some point between 1989 and 2071. The model is programmed in SAS. For each model run we scale up the outputs to a total adult population size in the current base year (i.e. usually the year we are living in at the time or the following year) of 10,000,000.

2.1 General population death rates and determination of age in 1989

The initial age distribution for both males and females is sampled for each population simulation from three possible distributions representing three different population demographic structures (Table S1). These are chosen such that in the absence of HIV, and given the death rates assumed (Table S2), the resulting population pyramids and growth rates represent the range of those seen across the setting scenarios (4). Thus a proportion of simulated people have an age below 15 in 1989 (and most are yet to be born). The only variable that is modelled and updated up to reaching the age of 15 (when becoming potentially sexually active) is age itself. The "youngest" person in 1989 is age -67 (i.e. will be born in 2056 and reach age 15 in 2071, just before the modelled period ends.

Table S1. Distribution of ages of simulated individuals in 1989

Age group	Probability of being in age group in 1989<br>Population demographic structure 1	Population demographic structure 2	Population demographic structure 3
-68 to -56	0.180	0.150	0.128
-55 to -46	0.165	0.130	0.119
-45 to -36	0.144	0.120	0.113
-35 to -26	0.114	0.110	0.104
-25 to -16	0.090	0.100	0.097
-15 to -6	0.080	0.090	0.090
-5 to 4	0.068	0.080	0.081
5 to 14	0.047	0.065	0.074
15 to 24	0.036	0.048	0.060
25 to 34	0.027	0.040	0.050
35 to 44	0.021	0.030	0.038
45 to 54	0.016	0.021	0.026
55 to 64	0.012	0.016	0.020

Age specific death rates for uninfected people are based on death rates in South Africa in 1997 (before the significant impact of HIV-related deaths) and are shown in Table S2.

Table S2. Age specific death rates (per year)

Age group	Annual death rate	Age group	Annual death rate
Males		Females	
15 – 19	0.0020	15 – 19	0.0015
20 – 24	0.0032	20 – 24	0.0028
25 – 29	0.0058	25 – 29	0.0040
30 – 34	0.0075	30 – 34	0.0040
35 – 39	0.0080	35 – 39	0.0042
40 – 44	0.0100	40 – 44	0.0055
45 – 49	0.0120	45 – 49	0.0075
50 – 54	0.0190	50 – 54	0.0110
55 – 59	0.0250	55 – 59	0.0150
60 – 64	0.0350	60 – 64	0.0210
65 – 69	0.0450	65 – 69	0.0300
70 – 74	0.0550	70 – 74	0.0380
75 – 79	0.0650	75 – 79	0.0500
80 – 84	0.1000	80 – 84	0.0700
≥85	0.4000	≥85	0.1500

2.2 Pregnancy and parity

Pregnancy is modelled at the point of childbirth. In a given three-month period, a women has a pregnancy risk (i.e. modelled as childbirth) if she had one or more short- or long-term condomless partners 9 months previously. The baseline probability per condomless partner of a woman being

pregnant in each three-month period is fixed throughout the simulation. This can be modified by a number of population-level and individual-level factors, described in Table S3. Pregnancy risk is calculated separately for each condomless partner. 95% of women are assumed to ever be able to become pregnant and each woman can have a maximum of 10 children. A woman can become pregnant 6 months after their previous birth.

Table S3. Factors affecting risk of pregnancy

<table>
<tr><td>Factor</td><td>Value</td></tr>
<tr><td>Baseline pregnancy risk per three months, prob_pregnancy_base</td><td>0.03 + U(0,1)*0.08. Based on fertility data from (4)</td></tr>
<tr><td>Modifier for overall population fertility</td><td>Overall population-level fertility rate can be higher or lower than the base assumption, sampled from distribution shown below.
<table>
<tr><td>Probability</td><td>33%</td><td>33%</td><td>33%</td></tr>
<tr><td>Factor applied to prob_pregnancy_base</td><td>1.75</td><td>1.0</td><td>1/1.75</td></tr>
</table></td></tr>
<tr><td>Age</td><td>Pregnancy probability is affected by a woman's age. Based on fertility data from (4)
<table>
<tr><td>Age (years)</td><td>15-24</td><td>25-34</td><td>35-44</td><td>45-54</td><td>55+</td></tr>
<tr><td>Value of fold_preg</td><td>2</td><td>1.9</td><td>1.0</td><td>0.2</td><td>0</td></tr>
</table></td></tr>
<tr><td>Pregnancy risk from short-term condomless partner</td><td>If a woman had one or more short-term condomless partners 9 months previously, pregnancy risk is reduced (per partner) due to the assumed lower number of sex acts with a short-term compared to long-term partner. The factor fold_tr_newp is applied at population level and is the same reduction that also applies to HIV transmission risk from a short-term condomless partner (see Table S27).
<table>
<tr><td>Probability</td><td>33%</td><td>33%</td><td>33%</td></tr>
<tr><td>Value of fold_tr_newp</td><td>0.3</td><td>0.5</td><td>0.7</td></tr>
</table></td></tr>
<tr><td>Desire for more children</td><td>It is assumed that women aged 25-54 years stop desiring more children at a rate of 0.5% per 3 months (termed rate_want_no_more_children), regardless of parity. These women have an 80% reduction in pregnancy risk at each time step.</td></tr>
</table>

Risk of mother to child transmission is dependent on the viral load of the mother at birth: viral load > 100,000: 40% risk, 10,000 – 100,00: 20%, 1000 – 10,000: 10%, < 1000: 0.02%. Risk of a neural tube defect (NTD) due to dolutegravir applies to women on dolutegravir in the relevant period of conception.

3 Sexual behaviour and risk of HIV acquisition

Here we describe the approach to modelling sexual behaviour and HIV acquisition. The basic approach is summarized in Figure S1. The distributions for parameter values related to sexual behaviour were chosen to reflect uncertainty and variability between settings. Sexual behaviour is characterized by two variables representing, respectively, the number of short term condomless sex partners and whether the person has a current long term condomless sex partner in the 3 month period. The status of long term partners is tracked over time (i.e. if they are infected, diagnosed, on ART, have viral load suppression). Short term partners are not tracked over time, in that if a person has a short term partner in time period *t* who is infected with HIV, this is independent of the probability that any short term partner in time *t*+1 is infected with HIV.

Figure S1 Summary of modelling of sexual behaviour and HIV acquisition

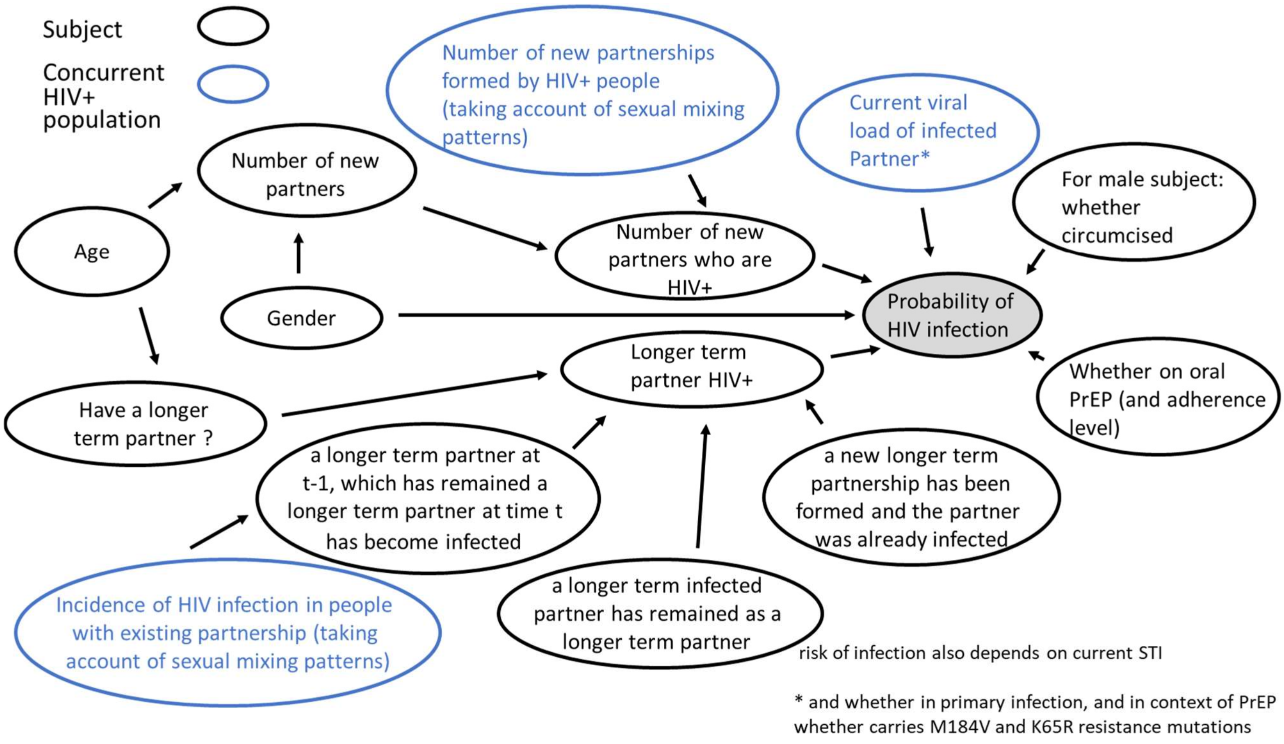

3.1 Determination of number of short term (condomless sex) partners at period t

The number of short term partners for an individual in a given period (termed *newp*) is generated at random, according to which sexual behaviour group the person is in for the period. Changes in the sexual behaviour group from time *t*-1 to time *t* are determined by transition probabilities between the groups. These differ for men, women (who are not sex workers) and female sex workers, and can be affected by multiple other factors described below.

For men, there are four sexual behaviour groups: (i) no short term condomless partners in 3 month period, (ii) low number of short term partners (*n*=1-3), (iii) medium number of short term partners (*n*=4-9), and (iv) high number of short term partners (*n*=10-35; Table S4).

For women (who are not sex workers), there are two sexual behaviour groups: (i) no short term condomless partners in 3 month period, (ii) one or more short term condomless partners in 3 month period (*n*=1-9; Table S5). Younger women (age 15-24) can have up to nine short-term condomless partners in a three month period and women aged 25 and older can have up to three.

For female sex workers, there are five sexual behaviour groups: (i) no short term condomless partners in 3 month period, (ii) low number of short term partners (*n*=1-6), (iii) medium number of short term partners (*n*=7-20), (iv) high number of short term partners (*n*=21-51) and (v) very high number of short term partners (*n*=51-150; Table S6). Sex workers aged more than 30 years were limited to a maximum of 30 short-term condomless partners.

Table S4. Distribution of number of short-term condomless partners within each risk category for men

Risk category							
Zero (*n* = 0)	Number of short-term condomless partners	0					
	Probability	100%					
Low (*n* = 1-3)	Number of short-term condomless partners	1	2	3			
	Probability	50%	30%	25%			
Medium (*n* = 4-9)	Number of short-term condomless partners	4	5	6	7	8	9
	Probability	35%	21%	17%	13%	9%	5%
High (*n* = 10-35)	Number of short-term condomless partners	10	15	20	25	30	35
	Probability	60%	20%	10%	5%	4%	1%

Table S5. Distribution of number of short-term condomless partners within each risk category for women

Risk category										
Zero	Number of short-term condomless partners	0								
	Probability	100%								
Any (*n* = 1-9)	Number of short-term condomless partners	1	2	3	4	5	6	7	8	9
	Age 15-24									
	Probability	30%	20%	15%	12%	9%	6%	4%	2%	2%
	Age 25+									
	Probability	70%	15%	15%	0%	0%	0%	0%	0%	0%

Table S6. Distribution of number of short-term condomless partners within each risk category for female sex workers

Risk category							
Zero (*n* = 0)	Number of short-term condomless partners	0					
	Probability	100%					
Low (*n* = 1-6)	Number of short-term condomless partners	1	2	3	4	5	6
	Probability	70%	10%	10%	5%	3%	2%
Medium (*n* = 7-20)	Number of short-term condomless partners	7-20					
	Probability	Uniform distribution across range					
High (*n* = 21-50)	Number of short-term condomless partners	21-50					
	Probability	Uniform distribution across range					
Very High (*n* = 51-150)	Number of short-term condomless partners	51-150					
	Probability	Uniform distribution across range					

All sex workers aged more than 30 years are limited to a maximum of 30 condomless partners in a three month period.

The transition probabilities p_{gija} of an individual moving from partner group *i* at *t*-1 to partner group *j* at *t* are given by

$$p_{gija} = \frac{f_{gij}}{\left(f_{gi1} + \sum_{j=2}^{k}(f_{gij} r_{ga})\right)} \text{ for j=1}$$

$$p_{gija} = \frac{f_{gij} r_{ga}}{\left(f_{gi} + \sum_{j=2}^{k}(f_{gij} r_{ga})\right)} \text{ otherwise}$$

(equation 1)

where *g* = 1,2,3 for males, females, and female sex workers, respectively, *a* = 1-10 for age groups 15-, 20-, 25-, 30-, 35-, 40-, 45-, 50-, 55-, 60-, respectively, and *k* is the number of short-term partner categories defined for that population group.

We considered 15 sets of values of f_{gij} each for males and females (who are not sex workers) as shown in Tables S7 and S8, and 5 sets of values for female sex workers (Table S9). These are characterized by substantially different intra-person variability over time in sexual behaviour subgroups. We randomly sampled a matrix independently for each gender for each model run.

Values of r_{ga} are shown in Table S10. These can be modified at time *t* by multiple factors, described in Table S11.

For each individual, actual transitions between groups were determined by random sampling at each time step *t*. The initial allocation of the population across the different risk groups is also sampled from a distribution at the start of each model run. The number of short-term condomless partners of

each individual for that time step, *newp*, is then sampled from the distribution for the appropriate risk group (Tables S4-S6).

For sex workers, the number of short-term condomless partners may then by modified further. Firstly, if there is a reduction in the overall behaviour of the population, leading to a reduced propensity for forming condomless partnerships (*rred_rc*<1), there is a possibility of an additional reduction (newp/3) in sex worker condomless partner numbers.

Probability	33%	33%	33%
Value of `p_rred_sw_newp`	0.01	0.03	0.10

Secondly, if a sex worker is engaged in a sex worker program there is a further chance that their short-term condomless partners are reduced by two-thirds for that time period, based on values of *effect_sw_prog_newp* i.e. the potential effect of a sex-worker program on the number of condomless partners .

Probability	33%	33%	33%
Value of `effect_sw_prog_newp`	0.10	0.20	0.30

Table S7. Values of $\boldsymbol{f_{1ij}}$ (values determining probability of transitioning between short term partner risk behaviour groups) for men.

	Short term partners group in period *t* (*j*)			
Short term partners group in period *t*-1 (*i*)	Zero (*n* = 0)	Low (*n* = 1-3)	Medium (*n* = 4-9)	High (*n* = 10-35)
Sexual behaviour transition matrix 1				
Zero	0.995	0.005	0.005	0.00005
Low	0.95	0.03	0.02	0.00005
Medium	0.03	0.07	0.90	0.00025
High	0	0	0.05	0.95
Sexual behaviour transition matrix 2				
Zero	0.98	0.01	0.01	0.00025
Low	0.98	0.01	0.01	0.00025
Medium	0.05	0.15	0.80	0.00125
High	0	0	0.20	0.80
Sexual behaviour transition matrix 3				
Zero	0.95	0.03	0.02	0.0005
Low	0.93	0.05	0.02	0.0005
Medium	0.20	0.20	0.60	0.0025
High	0	0	0.40	0.60
Sexual behaviour transition matrix 4				
Zero	0.995	0.005	0.005	0.0001

Low	0.95	0.03	0.02	0.0001
Medium	0.03	0.07	0.90	0.0005
High	0.04	0.04	0.09	0.83
Sexual behaviour transition matrix 5				
Zero	0.98	0.01	0.01	0.005
Low	0.98	0.01	0.01	0.0005
Medium	0.05	0.15	0.8	0.0025
High	0.025	0.06	0.17	0.75
Sexual behaviour transition matrix 6				
Zero	0.95	0.03	0.02	0.001
Low	0.93	0.05	0.02	0.001
Medium	0.20	0.20	0.60	0.005
High	0.04	0.08	0.21	0.67
Sexual behaviour transition matrix 7				
Zero	0.995	0.005	0.005	0.000025
Low	0.95	0.03	0.02	0.000025
Medium	0.03	0.07	0.90	0.000125
High	0	0	0.05	0.95
Sexual behaviour transition matrix 8				
Zero	0.98	0.01	0.01	0.000125
Low	0.98	0.01	0.01	0.000125
Medium	0.05	0.15	0.80	0.000625
High	0	0	0.20	0.80
Sexual behaviour transition matrix 9				
Zero	0.95	0.03	0.02	0.00025
Low	0.93	0.05	0.02	0.00025
Medium	0.20	0.20	0.60	0.00125
High	0	0	0.40	0.60
Sexual behaviour transition matrix 10				
Zero	0.90	0.06	0.04	0.0005
Low	0.99	0.005	0.005	0.0005
Medium	0.20	0.20	0.60	0.0025
High	0	0	0.40	0.60
Sexual behaviour transition matrix 11				
Zero	0.90	0.06	0.04	0.001
Low	0.99	0.005	0.005	0.001
Medium	0.20	0.20	0.60	0.005
High	0.04	0.08	0.21	0.67
Sexual behaviour transition matrix 12				

Zero	0.90	0.06	0.04	0.00025
Low	0.99	0.005	0.005	0.00025
Medium	0.20	0.20	0.60	0.00125
High	0	0	0	1.00
Sexual behaviour transition matrix 13				
Zero	0.75	0.15	0.10	0.0005
Low	0.99	0.005	0.005	0.0005
Medium	0.90	0.05	0.03	0.02
High	0.90	0.05	0.03	0.02
Sexual behaviour transition matrix 14				
Zero	0.75	0.15	0.10	0.001
Low	0.99	0.05	0.02	0.001
Medium	0.95	0.03	0.01	0.01
High	0.95	0.03	0.01	0.01
Sexual behaviour transition matrix 15				
Zero	0.75	0.15	0.10	0.00025
Low	0.93	0.05	0.02	0.00025
Medium	0.80	0.10	0.05	0.05
High	0.80	0.10	0.05	0.05

Table S8. Values of f_{2ij} (values determining probability of transitioning between short term partner risk behaviour groups) for women.

	Short term partners group in period *t* (*j*)	
Short term partners group in period *t*-1 (*i*)	Zero (n = 0)	Any (n = 1-9)
Sexual behaviour transition matrix 1		
Zero (n = 0)	0.995	0.005
Any (n = 1-9)	0.99	0.01
Sexual behaviour transition matrix 2		
Zero (n = 0)	0.995	0.005
Any (n = 1-9)	0.98	0.02
Sexual behaviour transition matrix 3		
Zero (n = 0)	0.995	0.005
Any (n = 1-9)	0.95	0.05
Sexual behaviour transition matrix 4		
Zero (n = 0)	0.995	0.005

Any (n = 1-9)	0.85	0.15
**Sexual behaviour transition matrix 5**		
Zero (n = 0)	0.995	0.005
Any (n = 1-9)	0.75	0.25
**Sexual behaviour transition matrix 6**		
Zero (n = 0)	0.99	0.01
Any (n = 1-9)	0.99	0.01
**Sexual behaviour transition matrix 7**		
Zero (n = 0)	0.99	0.01
Any (n = 1-9)	0.98	0.02
**Sexual behaviour transition matrix 8**		
Zero (n = 0)	0.99	0.01
Any (n = 1-9)	0.95	0.05
**Sexual behaviour transition matrix 9**		
Zero (n = 0)	0.99	0.01
Any (n = 1-9)	0.85	0.15
**Sexual behaviour transition matrix 10**		
Zero	0.99	0.01
Any (n = 1-9)	0.75	0.25
**Sexual behaviour transition matrix 11**		
Zero (n = 0)	0.98	0.02
Any (n = 1-9)	0.99	0.01
**Sexual behaviour transition matrix 12**		
Zero (n = 0)	0.98	0.02
Any (n = 1-9)	0.98	0.02
**Sexual behaviour transition matrix 13**		
Zero (n = 0)	0.98	0.02
Any (n = 1-9)	0.95	0.05
**Sexual behaviour transition matrix 14**		
Zero (n = 0)	0.98	0.02
Any (n = 1-9)	0.95	0.05
**Sexual behaviour transition matrix 15**		
Zero (n = 0)	0.98	0.02

Any (*n* = 1-9)	0.75	0.25

Table S9. Values of f_{3ij} (values determining probability of transitioning between short term partner risk behaviour groups) for female sex workers.

	Short term partners group in period t (*j*)				
Short term partners group in period *t*-1 (*i*)	Zero (*n* = 0)	Low (*n* = 1-3)	Medium (*n* = 4-9)	High (*n* = 10-35)	Very High (*n* = 51-150)
Sexual behaviour transition matrix 1					
Zero	0.80	0.17	0.015	0.010	0.005
Low	0.15	0.80	0.030	0.015	0.005
Medium	0.05	0.10	0.80	0.045	0.005
High	0.025	0.025	0.10	0.80	0.05
Very High	0.025	0.025	0.05	0.101	0.80
Sexual behaviour transition matrix 2					
Zero	0.90	0.10	0.00	0.00	0.00
Low	0.10	0.80	0.10	0.00	0.00
Medium	0.00	0.10	0.80	0.10	0.00
High	0.00	0.00	0.10	0.80	0.10
Very High	0.00	0.00	0.00	0.10	0.90
Sexual behaviour transition matrix 3					
Zero	0.99	0.01	0.00	0.00	0.00
Low	0.01	0.98	0.01	0.00	0.00
Medium	0.00	0.01	0.98	0.01	0.00
High	0.00	0.00	0.01	0.98	0.01
Very High	0.00	0.00	0.00	0.01	0.99
Sexual behaviour transition matrix 4					
Zero	0.96	0.01	0.01	0.01	0.01
Low	0.01	0.96	0.01	0.01	0.01
Medium	0.01	0.01	0.96	0.01	0.01
High	0.01	0.01	0.01	0.96	0.01
Very High	0.01	0.01	0.01	0.01	0.96
Sexual behaviour transition matrix 5					
Zero	1.00	0.00	0.00	0.00	0.00
Low	0.00	1.00	0.00	0.00	0.00
Medium	0.00	0.00	1.00	0.00	0.00
High	0.00	0.00	0.00	1.00	0.00
Very High	0.00	0.00	0.00	0.00	1.00

Table S10. Baseline values of r_{ga} (factor determining basic* relative level of sexual risk activity by age and gender)

Age group (a=1,10)	Pattern 1 Probability = 15%		Pattern 2 Probability = 15%		Pattern 3 Probability = 30%		Pattern 4 Probability = 30%	
	Males (g=1)	Females (g=2)	Males (g=1)	Females (g=2)	Males (g=1)	Females (g=2)	Males (g=1)	Females (g=2)
15-	0.30	1.80	0.65	1.10	0.05	2.50	0.05	3.00
20-	0.40	1.80	0.65	1.10	0.20	2.50	0.30	3.00
25-	0.85	1.00	1.20	1.00	1.00	1.00	0.80	1.00
30-	1.00	0.80	1.20	0.85	1.00	0.85	0.70	0.85
35-	0.85	0.50	0.65	0.55	0.65	0.55	0.65	0.40
40-	0.50	0.35	0.50	0.45	0.50	0.45	0.50	0.30
45-	0.40	0.30	0.45	0.35	0.45	0.35	0.45	0.15
50-	0.35	0.10	0.40	0.25	0.35	0.03	0.35	0.03
55-	0.20	0.03	0.35	0.20	0.25	0.01	0.25	0.01
60-	0.15	0.02	0.30	0.20	0.15	0.01	0.15	0.01

* Before factors defined below in Table S11 are considered

Table S11. Factors modifying transition probabilities between categories of short-term condomless partners. See also Table S27 below.

<table>
<tr><th>Factor</th><th>Description</th><th>Value</th></tr>
<tr><td>newp_factor</td><td>Underlying propensity of whole population to form short-term partnership with condomless sex.</td><td>Sampled at start of simulation from distribution:
<table>
<tr><td>Probability</td><td>33%</td><td>33%</td><td>33%</td></tr>
<tr><td>Value of newp_factor</td><td>0.5</td><td>1</td><td>2</td></tr>
</table></td></tr>
<tr><td>rred_a</td><td>Age-related factor describing the relative propensity of each five-year age group to form short-term condomless partnerships. These are selected at the start of the model run and can be modified at each time step to balance the number of short-term partnerships with condomless sex between different age groups within the population.</td><td>The initial age-specific factors are sampled from the distribution shown in Table S10.
The balancing modifier is calculated every time step within model. Equals 1 if partnerships balance across the sexes, <1 if the number of age-specific partnerships formed by one sex outweighs that reported by the other and vice versa.</td></tr>
<tr><td>rred_p</td><td>Person-specific factor reflecting a person's propensity to be in a higher or lower risk category for short-term condomless partners</td><td>The population-level propensity for fewer short-term condomless partners (termed p_rred_p) is sampled at the start of the simulation from the distribution shown below. Individual-level values are then sampled with p_rred_p defining the probability that rred_p=0.00001; otherwise rred_p=1.
<table>
<tr><td>Probability</td><td>33%</td><td>33%</td><td>33%</td></tr>
<tr><td>Value of p_rred_p</td><td>0.3</td><td>0.5</td><td>0.7</td></tr>
</table></td></tr>
<tr><td>rred_adc</td><td>Factor reducing the chance of transitioning to higher risk category for short-term partnerships with condomless sex for people with a AIDS-defining condition</td><td>0.2</td></tr>
<tr><td>rred_adhav</td><td>Whether there is a tendency for people with lower ART adherence to be people who tend to also have higher numbers of short-term condomless partners.</td><td>In 20% of runs, people with an adherence score of less than 0.8 will have rred_adhav=2.0.</td></tr>
</table>

<table>
<tr><td>rred_d</td><td>Possible reduction in condomless sex following a positive HIV test</td><td>Takes value ch_risk_diag_newp within six months of diagnosis and the square root of ch_risk_diag_newp thereafter. ch_risk_diag_newp is sampled at the start of the simulation for the whole population from the distribution shown below. Informed by Fonner et al (5)
<table>
<tr><td>Probability</td><td>25%</td><td>25%</td><td>25%</td><td>25%</td></tr>
<tr><td>ch_risk_diag_newp</td><td>0.7</td><td>0.8</td><td>0.9</td><td>1.0</td></tr>
</table></td></tr>
<tr><td>rred_balance</td><td>Factor to balance the absolute number of short-term condomless partners between men and women across the population.</td><td>Calculated every time step within model. Equals 1 if total partnerships balance across the sexes, <1 if the number of partnerships formed by one sex outweighs that reported by the other and vice versa.</td></tr>
<tr><td>rred_rc</td><td>Factor representing population-level behaviour change through time with respect to the number of condomless sex partners. In addition, for sex workers, the existence and effectiveness of a sex worker program can affect the number of condomless sex partners.</td><td>From 1995-2000, there is a general reduction in condomless sex among the whole population, determined by ych_risk_beh_newp according to the distribution, informed by [6, 7]:
<table>
<tr><td>Probability</td><td>20%</td><td>60%</td><td>20%</td></tr>
<tr><td>Value of ych_risk_beh_newp</td><td>0.6</td><td>0.7</td><td>0.8</td></tr>
</table>
From 2010-2015, there is the possibility of a further behavioural change, determined by ych2_risk_beh_newp according to the distribution:
<table>
<tr><td>Probability</td><td>5%</td><td>5%</td><td>80%</td><td>5%</td><td>5%</td></tr>
<tr><td>Value of ych2_risk_beh_newp</td><td>0.95</td><td>0.99</td><td>1</td><td>1 / 0.99</td><td>1 / 0.95</td></tr>
</table>
For sex workers, the impact of any sex worker program (starting in 2015 in 20% of all model runs) can also modify risk.</td></tr>
<tr><td>rred_ep</td><td>Population-level factor modifying chance of transitioning to higher risk category for short-term condomless partnership for those in a long-term partnership.</td><td>Takes value conc_ep, sampled at the start of the simulation for the whole population from the distribution shown below.
<table>
<tr><td>Probability</td><td>33%</td><td>33%</td><td>33%</td></tr>
<tr><td>Value of conc_ep</td><td>0.33</td><td>1.0</td><td>3.0</td></tr>
</table></td></tr>
</table>

3.2 Determination of having a long term (condomless sex) partner at period t

Note that only condomless sex partnerships are modelled. Thus if a person has a long term partner but condoms are used on all occasions of sexual intercourse then this is not counted as having a long term condomless sex partner, and rates of starting and stopping condomless partnerships can represent changing condom use within an existing long-term partnership as well as the initiation or termination of a new partnership itself.

3.2.1 Starting a new long term condomless partnership at period t

At each period, people with no current long term partner have an age-dependent probability of forming a new long term partnership, termed *eprate* (equation 2 and Table S12).

$$eprate = \frac{0.1e^{0.25*N(0,1)}}{a}$$

(equation 2)

Table S12. Values of *a*, the age modifier for *eprate*

Age group	15-34	35-44	45-54	55-64
A	1	2	3	5

The probability of starting a new long-term condomless partnership at time *t* is modified by several other factors, described in Table S12.

Table S13. Factors modifying the probability of starting a new long-term condomless partnership.

<table>
<tr><th>Factor</th><th>Description</th><th>Value</th></tr>
<tr><td>ch_risk_beh_ep</td><td>Factor representing possibility of population-level behaviour change through time with respect to condom use in long-term partnerships.</td><td>Calculation of ch_risk_beh_ep over time:
<table>
<tr><td>Time period</td><td>≤1995</td><td>1995-2000</td><td>>2000</td></tr>
<tr><td>Value of ch_risk_beh_ep</td><td>1</td><td>ych_risk_beh_ep ^ (t-1995)</td><td>ych_risk_beh_ep ^ (2000-1995)</td></tr>
</table>
Distribution of ych_risk_beh_ep:
<table>
<tr><td>Probability</td><td>25%</td><td>25%</td><td>25%</td><td>25%</td></tr>
<tr><td>Value of ych_risk_beh_ep</td><td>0.80</td><td>0.90</td><td>0.95</td><td>1.00</td></tr>
</table></td></tr>
<tr><td>ch_risk_diag</td><td>Possible adjustment in condomless sex in long-term partnerships following a positive HIV test.</td><td>Distribution of ch_risk_diag:
<table>
<tr><td>Probability</td><td>25%</td><td>25%</td><td>25%</td><td>25%</td></tr>
<tr><td>Value of ch_risk_diag</td><td>0.7</td><td>0.8</td><td>0.9</td><td>1.0</td></tr>
</table></td></tr>
</table>

<table>
<tr>
<td>Balancing partnerships</td>
<td>Adjustment to the probability of forming a new long-term condomless partnership in order to balance the total number reported by men and women within the simulated population, updated each time step.</td>
<td>Uses the ratio of long-term condomless partnerships reported by men compared to women, termed r_ep_mw, to adjust the probability of starting a new partnership by either sex.
<table>
<tr><td>Value of r_ep_mw</td><td><0.8</td><td>0.8-</td><td>0.9-</td><td>1.1-</td><td>>1.2</td></tr>
<tr><td>Adjustment to probability of partnership formation</td><td>x4 for men</td><td>x2 for men</td><td>No change</td><td>x2 for women</td><td>x4 for women</td></tr>
</table>
</td>
</tr>
</table>

At the time a long term partnership is started, it is classified into 3 duration groups, each with a different tendency to endure. The percent of people in each group is dependent on age and is shown in Table S14.

Table S14. Percent of newly formed long term partnerships classified into each of three duration groups, each of which has a different tendency to endure (higher class, more durable).

Age group	Partnership duration group		
	1	2	3
15-44	30%	30%	40%
45-54	30%	50%	20%
55-64	30%	70%	0%

3.2.2 Stopping a long term condomless partnership at period t

At time period *t*, for people with a long term partner, the probability of the condomless sex partnership continuing with respect to their partnership duration group is shown in Table S15. It is also modified by *ch_risk_beh_ep*, the parameter conveying the population level change in sexual behaviour with long term partners that occurs from 1995-2000, defined in Table S13. Further, the probability of the partnership ending is reduced by a factor *ch_risk_diag* in the 3 month period after an HIV diagnosis for either the individual or partner.

Table S15. Probability of long-term partnership continuing in time period *t.*

Duration category	1	2	3
Partnership continuation probability	$1-\frac{0.25}{ch\_risk\_beh\_ep}$	$1-\frac{0.05}{ch\_risk\_beh\_ep}$	$1-\frac{0.02}{ch\_risk\_beh\_ep}$

The probability that a partnership continues is also modified by an age- and sex-specific factor that is updated each time step to ensure that the numbers of long-term condomless partners reported by each age group approximately mirror the reciprocal number reported by the opposite sex.

Note also that levels of sexual behaviour, in terms of numbers of short term partners and the probability of a long term partner are essentially determined by the levels of such sexual behaviour required in order to produce an epidemic as described, given rates of transmission with condomless sex partners. Sexual behaviour tends to be under-reported particularly in women and higher levels of behaviour have to be assumed both to be consistent with levels of risk behaviour reported in men, and to generate an epidemic of the proportions observed (e.g. 6-10).

3.3 Determination of number of short term (condomless sex) partners who are HIV infected at time t

For each short term partner that a subject has at time *t*, the probability that the partner is infected is calculated. This is dependent on the prevalence of HIV in those of the opposite gender themselves having short term partners, taking consideration of age mixing. If the subject is of gender *g* and age group *a*, then for each short term partner the first step is to determine by sampling at random, the age group of the short term partner, a^{newp} (in fact, for simplicity, all short term partners at time *t* are assumed to be in this same age group). The gender and age mixing probabilities used are sampled independently for each gender in each run from the matrices shown in Table S16.

Table S16. Sexual mixing matrices by age and gender. The proportion of short term partnerships formed by men in age group a_m which are with females of age group a_f and the proportion of short term partnerships formed by females in age group a_f which are with men of age group a_m.

A. Men					
	Female age groups (a_f)				
Male age groups (a_m)	15-24	25-34	35-44	45-54	55-65
Sex age mixing matrix 1					
15-24	0.865	0.11	0.025	0.00	0.00
25-34	0.47	0.43	0.10	0.00	0.00
35-44	0.30	0.50	0.20	0.00	0.00
45-54	0.43	0.30	0.23	0.03	0.01
55-64	0.18	0.18	0.27	0.27	0.10
Sex age mixing matrix 2					
15-24	0.865	0.11	0.025	0.00	0.00
25-34	0.47	0.43	0.10	0.00	0.00
35-44	0.20	0.35	0.40	0.05	0.00
45-54	0.15	0.23	0.25	0.30	0.07
55-64	0.05	0.08	0.25	0.30	0.32
Sex age mixing matrix 3					
15-24	0.90	0.05	0.02	0.02	0.01
25-34	0.44	0.43	0.10	0.02	0.01

35-44	0.20	0.34	0.40	0.05	0.01
45-54	0.15	0.23	0.25	0.30	0.07
55-64	0.05	0.08	0.25	0.30	0.32
Sex age mixing matrix 4					
15-24	0.93	0.05	0.01	0.01	0.00
25-34	0.50	0.40	0.08	0.01	0.01
35-44	0.20	0.34	0.41	0.05	0.00
45-54	0.15	0.20	0.25	0.37	0.03
55-64	0.05	0.08	0.20	0.40	0.27
Sex age mixing matrix 5					
15-24	0.94	0.05	0.01	0.00	0.00
25-34	0.50	0.40	0.08	0.01	0.01
35-44	0.40	0.40	0.15	0.04	0.01
45-54	0.30	0.30	0.25	0.10	0.05
55-64	0.30	0.30	0.30	0.05	0.05
Sex age mixing matrix 6					
15-24	0.94	0.05	0.01	0.00	0.00
25-34	0.50	0.40	0.08	0.02	0.00
35-44	0.50	0.35	0.10	0.05	0.00
45-54	0.50	0.35	0.10	0.05	0.00
55-64	0.50	0.35	0.10	0.05	0.00

B. Women					
	Male age groups (a_m)				
Female age groups (a_f)	15-24	25-34	35-44	45-54	55-65
Sex age mixing matrix 1					
15-24	0.43	0.34	0.12	0.10	0.01
25-34	0.09	0.49	0.30	0.10	0.02
35-44	0.03	0.25	0.34	0.25	0.13
45-54	0.00	0.00	0.05	0.70	0.25
55-64	0.00	0.00	0.00	0.10	0.90
Sex age mixing matrix 2					
15-24	0.43	0.415	0.12	0.03	0.005
25-34	0.09	0.50	0.35	0.05	0.01
35-44	0.03	0.25	0.34	0.25	0.13
45-54	0.00	0.00	0.05	0.70	0.25
55-64	0.00	0.00	0.00	0.10	0.90
Sex age mixing matrix 3					
15-24	0.25	0.55	0.15	0.03	0.02
25-34	0.09	0.50	0.35	0.05	0.01
35-44	0.03	0.25	0.34	0.25	0.13
45-54	0.00	0.00	0.05	0.70	0.25
55-64	0.00	0.00	0.00	0.10	0.90

Sex age mixing matrix 4					
15-24	0.05	0.55	0.35	0.03	0.02
25-34	0.03	0.52	0.40	0.03	0.02
35-44	0.03	0.05	0.57	0.30	0.05
45-54	0.00	0.00	0.05	0.70	0.25
55-64	0.00	0.00	0.00	0.10	0.90

Sex age mixing matrix 5					
15-24	0.05	0.45	0.30	0.15	0.05
25-34	0.01	0.40	0.39	0.15	0.05
35-44	0.01	0.07	0.47	0.30	0.15
45-54	0.00	0.00	0.05	0.70	0.25
55-64	0.00	0.00	0.00	0.10	0.90

Sex age mixing matrix 6					
15-24	0.20	0.20	0.20	0.20	0.20
25-34	0.00	0.25	0.25	0.25	0.25
35-44	0.01	0.01	0.32	0.33	0.33
45-54	0.00	0.00	0.05	0.70	0.25
55-64	0.00	0.00	0.00	0.10	0.90

Then, for the given partner (of gender 1-*g* and age group a^{newp}), the risk that the partner is infected is then given by

$$h_{gat} = \frac{\sum_{a^{\text{newp}},(g-1)} L_{(t-1)}^{\text{inf}}}{\sum_{a^{\text{newp}},(g-1)} L_{(t-1)}}$$

(equation 3)

where $L_{(t-1)}^{\text{inf}}$ is the total number of infected short-term partners at time (*t*-1), and $L_{(t-1)}$ is the total number of short term partners at time *t*-1. The numerator is therefore the total number of infected short term partnerships of the opposite gender in age group a^{newp}..

Since we assume that all short term partners at time *t* are in this same age group, the total number of infected short term partners that the subject has at time *t*, L_t^{inf}, is then given by

$$L_t^{\text{inf}} = \text{Min}(\text{Poisson}(h_t \cdot L_t), L_t)$$

(equation 4)

3.4 Determination of probability that a long term partner is HIV infected at time t

E_t^{inf} indicates whether the subject has a long term (condomless sex) partner who is infected ($E_t^{\text{inf}} = 1$ if infected, else $E_t^{\text{inf}} = 0$). A long term partner at time *t* can be infected either because (i) a new long term partnership has been formed and the partner was already infected, (ii) because a long term partner at *t*-1, which has remained a long term partner at time *t*, has become infected, or (iii) because an infected long term partner has remained as a long term partner.

For (i):

It is assumed that 50% of new long-term condomless partners were previously a long-term condomless partner of the individual (for example, if condom use has started and then stopped within an ongoing partnership); for these individuals, if the partner was previously recorded as being HIV-infected then they remain HIV-infected. For new partners who are not known to be already infected, the probability of infection is based on the HIV prevalence of infection in the previous time step among subjects of age group *a* and gender 1-*g* (equation 5).

$$\begin{cases} E_t^{\text{inf}} = 1, \ U < p_{a(1-g)(t-1)} & \text{where } U \text{ randomly sampled from } Uniform(0,1) \\ E_t^{\text{inf}} = 0, \ \text{otherwise} & \end{cases}$$

(equation 5)

where $p_{a(1-g)(t-1)}$ is the HIV prevalence.

For (ii):

The probability that a long term partner of a subject of age group *a* and gender *g* becomes infected from a different partner is derived from the HIV incidence at *t*-1 for age group *a* (i.e. the same age group) and gender 1-*g*, $i_{a(1-g)(t-1)}$ among the sexually active population who have both a long term partner and at least one short term partner (equation 6).

$$\begin{cases} E_t^{\text{inf}} = 1, & U < i_{a(1-g)(t-1)} \ \text{where } U \text{ randomly sampled from } Uniform(0,1) \\ E_t^{\text{inf}} = 0, & \text{otherwise} \end{cases}$$

(equation 6)

In order to maintain balance, for each gender, between the number of uninfected people with a long term partner who is infected, and the number of infected people with a long term partner who is uninfected, this incidence $i_{a(1-g)(t-1)}$ is modified at time *t* dependent on the degree of balance at time *t*-1.

For (iii):

If $E_{(t-1)}^{\text{inf}} = 1$ and $E_t \geq 1$ then assign $E_t^{\text{inf}} = 1$

(equation 7)

3.5 Determination of the risk of infection from a short term partner to the subject

For each HIV infected short term partner of a subject of gender *g* and age group *a* the viral load group, *v*, of the partner is obtained by sampling from the viral load distribution of those of the opposite gender. Thus we sample from Uniform(0,1), where the probability of the partner having viral load in group *v* is given by

$$\frac{\sum_v L_{(t-1)}^{\text{inf}}}{\sum L_{(t-1)}^{\text{inf}}}$$

(equation 8)

where the numerator is the total number of short-term partnerships had by infected people in viral load group *v* and the denominator is the total number of short-term partnerships had by infected people (in any viral load group).

Viral load groups are:

(1) < 2.7 log cps/mL
(2) 2.7-3.7 log cps/mL
(3) 3.7-4.7 log cps/mL
(4) 4.7-5.7 log cps/mL
(5) $\geq$ 5.7 log cps/mL
(6) primary infection.

Once the viral load group, *v*, of the infected partner is determined, the probability, t_v, of the subject being infected by the partner is then given according to:

(1) t_1 = Normal (0.001*fold_tr_newp, 0.000025^2)
(2) t_2 = Normal (0.01*fold_tr_newp*fold_tr, 0.0025^2)
(3) t_3 = Normal (0.03*fold_tr_newp*fold_tr, 0.0075^2)
(4) t_4 = Normal (0.06*fold_tr_newp*fold_tr, 0.015^2)
(5) t_5 = Normal (0.10*fold_tr_newp*fold_tr, 0.025^2)
(6) t_6 = Normal (0.16*fold_tr_newp, 0.075^2)

These are based on Hollingsworth et al (2008) (11) and Bellan (2015) (12), which are estimated for a longer term partner.

The transmission risk from a short term partner is multipled by *fold_tr_newp* due to the assumed lower number of sex acts in short-term partnerships and *fold_tr* for viral load groups 2-5 only to represent underlying variability in transmissibility across the whole population. These probabilities are increased by *fold_change_w*-fold for female subjects aged $\geq$ 20, by *fold_change_yw*-fold for female subjects aged < 20, by *fold_change_sti*-fold if the person has an existing STI (risk of a new STI in any one three month period is given by the number of short term condomless partners / 20, and risk of an STI persisting to each subsequent time step is given by the number of short term condomless partners / 5) (13), and decreased by 60% if a male partner is circumcised (14-16). The risk is decreased by 90% or 95% (determined by random sampling) if the subject is on PrEP with > 80% adherence (details of PrEP use are given below) (17) Full details of the effects of PrEP are described in Section 6. Each of these probabilities is sampled once for the whole simulation from the distributions shown in Table S27 below.

In 20% of model runs, it is assumed that short-term partners can have a lower level of viral load suppression (*exp_setting_lower_p_vl1000*) due to short-term migration; in these runs, for individuals aged 20-49 years, the probability of a short-term condomless partner being virally suppressed <1000 cells/mL is adjusted by 1/(1+uniform(0,1)) (termed '*external_exp_factor*') for 1% of men and 0.05% of women (*rate_exp_set_lower_p_vl1000*).

We assume that super-infection can occur (i.e. a person can be re-infected with HIV with consequent risk of acquiring new mutations).

Realization of whether the subject is infected by each short term partner is determined by sampling from Uniform(0,1).

3.6 Determination of the risk of infection from a long term partner to the subject

Infected long term partners at time t are classified by whether they are in primary infection (if infection occurred at *t*-1), whether they are diagnosed with HIV, whether they are on ART, and whether their current viral load is < 2.7 cps/mL or not.

The probability of a long term partner with HIV being diagnosed at time *t*, $p_t^{e,diag}$, is determined by the proportion of all HIV-infected long-term condomless partners who are diagnosed at time *t*-1, $p_{t-1}^{e,diag}$, adjusted according to the difference between this value and the proportion of subjects with HIV who are diagnosed, $\frac{T_{(t-1)}^{diag}}{T_{(t-1)}^{inf}}$ (equation 9).

$$\begin{cases} \text{if } d_{(t-1)}^{e,diag} < 0 & \text{then } p_t^{e,diag} = 0 \\ \text{if } 0 < d_{(t-1)}^{e,diag} \leq 0.05 & \text{then } p_t^{e,diag} = \frac{p_{t-1}^{e,diag}}{5} \\ \text{if } 0.05 < d_{(t-1)}^{e,diag} \leq 0.10 & \text{then } p_t^{e,diag} = \frac{p_{t-1}^{e,diag}}{2} \\ \text{if } 0.10 < d_{(t-1)}^{e,diag} & \text{then } p_t^{e,diag} = p_{t-1}^{e,diag} \end{cases}$$

$$\text{where } d_{(t-1)}^{e,diag} = \frac{T_{(t-1)}^{diag}}{T_{(t-1)}^{inf}} - p_{(t-1)}^{e,diag}$$

(equation 9)

$T_{(t-1)}^{diag}$ is the total number of subjects diagnosed with HIV at time *t*-1 and $T_{(t-1)}^{inf}$ is the total number of subjects with HIV (diagnosed and undiagnosed) at time *t*-1.

The proportion of those diagnosed who are on ART, and the proportion of those on ART who have viral load < 2.7 log cps/mL are determined in a similar manner. In this way the proportions diagnosed with HIV, on ART, and with current viral load is < 2.7 log cps/mL are kept similar for the long term partners as in the simulated subjects themselves.

Risk of infection from a long term infected partner is determined by Normal (0.001, 0.075^2) if the existing partner is in primary infection (ie. infected at *t*-1), Normal (0.16, 0.000025^2) if the existing partner has viral load < 2.7 log cps/mL, and Normal (0.05**fold_tr*, 0.0125^2) otherwise.

Similar to short-term partners, the transmission risk from a long-term partner is multipled by *fold_tr* for partners who are not in primary infection or virally suppressed to represent underlying variability in transmissibility across the whole population; by *fold_change_w*-fold for female subjects aged $\geq$ 20 and by *fold_change_yw*-fold for female subjects aged < 20; by *fold_change_sti*-fold if the person has an existing STI; and is decreased, exactly as for short term partners above, if a male partner is circumcised or the subject is on PrEP.

3.7 Determination of the risk of infection from the subject to a long term partner

If the subject is infected and a long-term condomless partner is not, there is the possibility of HIV transmission from the subject to the partner. First, the probability that the subject is the long-term partner's only sexual partner is estimated from the proportion of subjects of that age group who only

have a long-term partner (compared to those who have a long-term and short-term condomless partners) in that time step.

For long-term partners in a monogamous partnership with the subject, the risk of HIV infection is based on the subject's viral load group, modified by *fold_tr*, *fold_change_w*, *fold_change_yw* and *fold_change_sti* (based on the subject rather than the partner's STI status) as described above.

For long-term partners who are assumed to also have other short-term condomless partners (who not explicitly modelled), risk of infection each time step is based on the HIV incidence for all subjects of that gender and age group who have a long-term partner and at least one short-term partners.

Balancing of infection rates from subjects to long-term partners is achieved by comparing the number discordant partnerships from the perspective of infected men with an uninfected female partner compared to uninfected women with an infected male partner (and vice versa) and adjusting the number of transmissions accordingly.

A further balancing adjustment is made to equalise the number of concordant positive partnerships reported by men and women by dissolving some partnerships if the ratio differs from one.

4 Transmitted resistance

The modelling of transmission of drug resistance is summarized in Figure S2. The presence or not of resistance mutations does not influence the risk of transmission (i.e. virus with resistance mutations present is assumed equally transmissible as virus without such mutations, for a given viral load). The probability that resistance mutations present in majority virus of the source partner are transmitted to the newly infected person is dependent on the specific mutation. Once a resistance mutation is transmitted to the new host it is assumed to have a certain probability of being lost from majority virus over time (18). Even after being lost from majority virus, it is assumed to remain in minority virus and is selected back as majority virus if an antiretroviral drug selecting for that mutation is initiated. We also consider the possibility of a person who is already infected become super-infected, including with drug resistant HIV (19), although there is assumed to be at most a 20% chance that a person super-infected by a person with HIV resistance then has virus with those resistance mutations as a result.

Figure S2. Overview of modelling of transmission of drug resistance

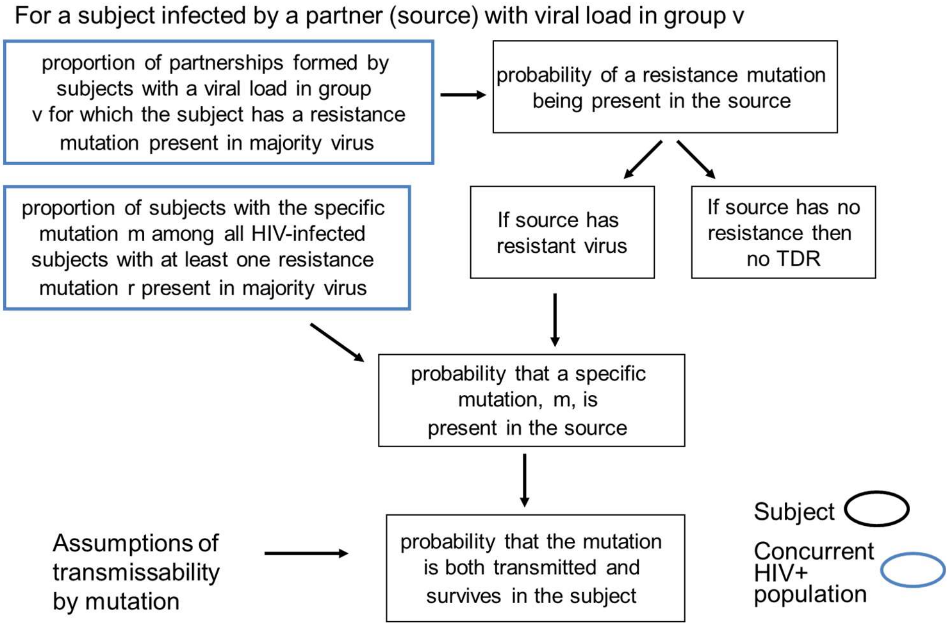

4.1 Transmitted resistance: details

The viral load group of the person who infected the subject is known, as indicated above. For a subject infected by a person in viral load group v the probability of a resistance mutation being present in the infected person is given by

$$\frac{\sum_{v,\ \text{and mutation present}} L_{(t-1)}^{\text{inf}}}{\sum_{v} L_{(t-1)}^{\text{inf}}}$$

where $\sum_{v,\text{ and mutation present}}$ is the sum over all partnerships had by HIV-infected people in viral load group v for whom a resistance mutation is present in majority virus and $\sum_{v}$ is the sum over all HIV-infected subjects in viral load group v. Again, realization of whether the subject is infected by a person with at least one resistance mutation in majority virus is determined by sampling from Uniform(0,1).

For subjects infected from a source partner with a resistance mutation, the probability that a specific mutation, m, is present in the source is given by

$$\frac{\sum_{\text{mutation } m \text{ present}} L_{(t-1)}^{\text{inf}}}{\sum_{\text{mutation present } v} L_{(t-1)}^{\text{inf}}}$$

Where $\sum_{\text{mutation } m \text{ present}}$ is the sum over all HIV-infected subjects with mutation m present in majority virus and $\sum_{\text{mutation present}}$ is the sum over all HIV-infected subjects with at least one resistance mutation in majority virus.

If a given resistance mutation, m, is present in the source partner, the probability that the mutation is both transmitted and survives in the subject (i.e. that its presence will affect future response to drugs for which the mutation confers reduced sensitivity) is mutation specific (Table S17). We consider

uncertainty in the extent to which NNRTI and INSTI transmitted resistance mutations are effectively immediately lost (even from minority virus) by sampling from a distribution for parameters *res_trans_factor_nnrti and res_trans_factor_ii*, which are sampled at the start of each run.

Table S17. Table of probabilities that for a given mutation present in the source partner the mutation is both transmitted and survives in the subject (based on evidence from studies comparing distribution of resistance mutations between treated and antiretroviral naïve populations; (e.g. 20, 21) and modelling of HIV in MSM in the UK (22)

M184V	0.2
K65R	0.2
Q151M	0.5
Thymidine analogue mutations (TAMS)	0.5
NNRTI mutations (K103N, G190A, Y181C)	1 - (0.20**res_trans_factor_nn*)
PI mutations	0.5
Dolutegravir mutations	1 - (0.20**res_trans_factor_ii*)

4.2 Loss from majority virus of transmitted mutations

There is a probability per 3 months of loss of persistence of transmitted mutations from majority virus to minority virus (same for each mutation) *rate_loss_persistence,* which is one of the parameters sampled at the start of each model run (see Table S27 below). This is again informed by fitting of a model of HIV in MSM to UK data UK (22)

5 People being hard to reach for services

A proportion of people have a long term propensity not to take up HIV services (including testing, PrEP, VMMC), for various possible reasons including stigma, physical barriers, etc. (23) ("hard to reach"). The proportion of women that have a propensity to be hard to reach with prevention and testing servcices is given by p_hard_reach_w (sampled from Uniform(0.05, 0.15) for each run / setting scenario; see Table S27). The extent to which this is higher in men is given by a parameter hard_reach_higher_in_men (sampled from Uniform(0,0.1).

Limited data are available to inform this parameter (proxy variables are the proportion who reported never being tested for HIV and, more precisely, the proportion who refuse HIV testing), nevertheless we considered it important to take this into account, given the evidence that not everyone accepts HIV testing for various reasons. The level of acceptability of provider initiated HIV testing and counselling (PITC) in resource limited settings is extremely variable from levels of 99%, observed in inpatients in Uganda (24) to 31% among outpatients in South Africa (25). Among pregnant women the level of acceptability of PITC seems to be higher, varying from 76 to 99.9% (26), while the estimated acceptability of home-based counselling and testing has been estimated in a meta-analysis to be 83% (27). This variability seems to be related mainly to the quality of the intervention delivered and calendar time.

6 Oral PrEP

6.1 Overview of modelling of oral PrEP

We consider various possible policies by which oral PrEP (tenofovir and lamividine (which is not distinguished from emtricitabine in the model)) is provided / available. By default we assume that, in the context of community-led PrEP with education and unrestricted availability, women and men will only use PrEP during 3 month time periods in which they have condomless sex with at least one short-term partner (*newp*), when they have a long term partner (ep) who is known to have HIV but is not on ART, or when a woman feels there is a high risk her long term partner is in this position (which is implemented as women aged under 50 without HIV and with a long term condomless sex partner who is not on ART having a 5% chance that she will be considered as fulfilling the criteria for risk_informed PrEP, which becomes 50% if her partner has HIV, based on the assumption that for women who suspect they are at risk are indeed at substantially higher risk that thir partner has HIV). We assume in this default approach that PrEP will not be used at other times (although this possibility is explored in sensitivity analyses). It is assumed that the aim is for daily PrEP use during such 3 month periods, although we account for < 100% adherence. We refer to this as *risk_informed PrEP*.

While in our main analyses we assume that all PrEP use will be risk_informed as descibed above, we do not assume that everyone fulfilling the above criteria for risk_informed PrEP will take it. Only a proportion of people are considered willing to take PrEP even when they fulfill the above risk_informed PrEP criteria (variable name: prep_willing_pop). This proportion is determined by the parameter *prepuptake_pop* which is sampled from a distribution for each run / setting scenario (see Table S27 below). Similarly *prepuptake_sw,* which indicates the additional chance of being willing to take PrEP if a woman is a sex worker, is also sampled. Further, the subset of people considered “hard to reach”, as described above, also will not start PrEP. A parameter *rate_test_startprep* represents the additional rate of being tested for HIV because of interest in PrEP in people who have never been on PrEP but who fulfill the criteria for risk_informed PrEP. If a person is willing to take PrEP and has tested negative and meets the criteria for risk_informed PrEP and is not hard to reach then there is a 0.75 probability of starting PrEP in the period.

PrEP continuation requires 3 monthly confirmation of HIV negative status in addition to continuing to fulfill the criteria for risk_informed PrEP. We also consider that people may stop PrEP despite continuing to fulfill the criteria for risk_informed PrEP. There is assumed to be a probability (*rate_choose_stop_prep*) of discontinuation per 3 months. If this is the case there is assumed to be a *prob_prep_restart_choice* chance of HIV testing and resumption per 3 month period in which the criteria for risk_informed PrEP are met thereafter. If a person stops PrEP due to no longer fulfilling the criteria for risk_informed PrEP then PrEP can be restarted (with probability *rate_test_restartprep*) if the person tests HIV negative and again fulfills the criteria.

We assume that PrEP has efficacy of 90% or 95% (each in 50% of setting scenarios) if adherence is > 80% (17) and the partner with HIV does not carry virus resistant to both emtricitabine/ lamivudine and TDF. PrEP adherence level for an individual, quantified on a scale of 0-100% is the proportion of the drug target level that is attained with condomless sex partners in a given 3-month period. We assume that 50% of adolescents and young adults (age 15-24 years) will be half as likely to adhere to PrEP compared to the rest of the population. The assumptions described result on average in 90% of people on PrEP having > 80% adherence. PrEP effectiveness, as opposed to efficacy, is what is measured in real life conditions and is assumed to be proportional (i.e .0.90 or 0.95-fold) to the PrEP

adherence level. In a person with current PrEP adherence of 80% when assuming efficacy of 0.95 the current effectiveness would be 0.95 x 80% = 76%.

PrEP is assumed to be only 50% effective when the partner has virus with both K65R and M184V resistance mutations that affect tenofovir and emtricitabine or lamivudine. (28)

PrEP can inadvertantly be used in people with HIV due to starting PrEP when already infected with HIV (caused by <100% HIV test sensitivity - we assume 98% in 80% of setting scenarios and 95% in 20% - or due to being in the primary infection window period), or becoming infected while taking PrEP (due to sub-optimal adherence, less than 100% PrEP efficacy, or infection with PrEP drug resistant virus). The risk of resistance emergence for persons who inadvertently take PrEP while having (drug sensitive) HIV is on average 7% and 38% with K65R and M184V respectively by 3 months of infection (29, 30).

7 Male Circumcision

Amongst those testing HIV negative, the probability of VMMC is influenced by the underlying VMMC rates (see Table S27 for values from which we sample), age and calendar year. The range of probabilities of VMMC according to age and calendar year reflects the range of VMMC prevalence across the region. VMMC is assumed to lead to a 60% lower risk of HIV acquisition from a given partner with HIV (14-16).

8 HIV progression in absence of treatment

Figure S3 gives an overview of the modelling of HIV natural history. The model of the natural history of HIV and the effect of antiretroviral therapy has been derived previously and compared with a range of observed data (see Phillips et al Lancet 2008 (31), AIDS 2011 (32), Nakagawa et al 2012 (33), 2015 (34) and associated supplementary material). Below we set out the structure of the model and explain what parameters represent.

Figure S3. Overview of modelling of natural history of HIV infection

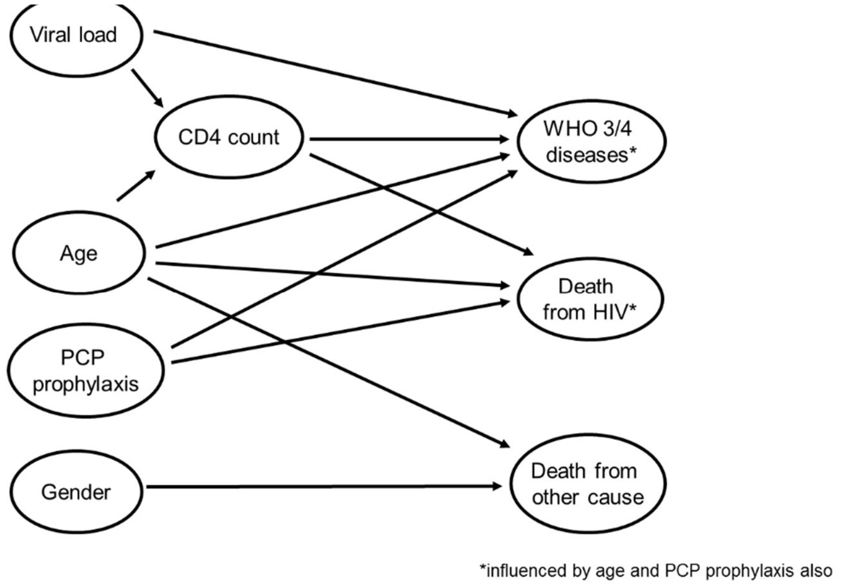

8.1 Determination of changes in viral load and CD4 count

Initial log_{10} viral load (V_{set}) is dependent on age and sex and is sampled from Normal(4.075,0.5^2) + ((age(t)-35)*0.005) for men and Normal(3.875,0.5^2) + ((age(t)-35)*0.005) for men

This viral load (V_{set}) is assumed to be that reached after primary infection. It is not used to determine the risk of transmission in primary infection itself.

Initial CD4 count, modelled on the square root scale, is partially dependent on initial viral load and given by

Square root CD4 count = *mean_sqrtcd4_inf* (= 27.5) - (1.5 x V_{set}) + Normal(0,2^2) – ((age – 35) x 0.05)

Initial virus is assumed to be R5-tropic. Shift to presence of X4 virus is assumed to depend on viral load. Probability of a shift per 3 months is given by 10^v x 0.0000004, where v is the current log_{10} viral load.

Viral load change (vc) from period t-1 to period t (i.e. in 3 months) is given by

vc(t-1(= (gx x 0.02275 + Normal(0, 0.05^2) + ((age(t-1) - 35) x 0.00075)

gx=1 viral load at t (v(t)) = v(t-1)+ vc(t-1)

CD4 count changes from period t-1 to t are dependent on the current viral load (i.e. viral load at time t-1) and are given by sampling from a Normal distribution with standard deviation *sd_cd4*=1.2 and mean *fx* (sampled from lognormal(1,0.2^2) as shown in Table S27) times the values as follows:

Viral load at t-1	Change in square root CD4 count (per 3 mths)
<3.0	+0.000
3.0-	+0.022
3.5-	+0.085
4.0-	-0.400
4.5-	-0.400
5.0-	-0.850
5.5-	-1.300
6.0-	-1.750

People with X4 virus present experience an additional change in square root CD4 count of -0.25.

These estimates were derived based on consideration of evidence from natural history studies (35-43) and were selected in conjunction with other relevant parameter values to provide a good fit to the incubation period distribution. Differences that have been found in initial viral load by sex, age and risk group are not currently incorporated in the model.

Table S18. Example model outputs of incubation period by age. Kaplan-Meier percent with WHO 4 Event. Compare with (44). This varies by model run due to the sampling of the value of the parameter *fx* (sampled from lognormal(1,0.2^2) as shown in Table S27).

Age at infection	1	3	5	10	15	20
15-	0.6%	4%	14%	50%	75%	89%
25-	1.1%	2%	23%	67%	88%	97%
35-	2.1%	13%	34%	82%	97%	100%
45-	3.7%	21%	54%	93%	100%	100%
55-	1.4%	24%	59%	96%	100%	100%

9 HIV testing and diagnosis of HIV infection

HIV testing was assumed introduced in 2003. People who are hard to reach have no possibility of getting tested for HIV unless symptomatic. For the remainder of the population (not hard to reach), increasing gender and age-specific rates of HIV testing (for the 1st time and for repeat testing) since 1996 were assumed (parameter *an_lin_incr_test* sampled at the start of each model run), to reflect the range of levels of testing observed in countries in SSA. This increase in testing is assumed to stop so that testing rates reach a plateau (*date_test_rate_plateau*). We assume some targeting of testing such that those having a condomless sex partner since last test are more likely to test – the degree of such targeting is conveyed by the parameter *test_targeting*. Pregnant women experience an additional probability of being tested in the ANC, which increases over calendar time (*rate_testanc_inc*).

People with acute symptoms (WHO stage 4, 3 or active TB) are assumed to have a higher chance of testing for HIV in that 3 month period and a higher chance of being linked to care once diagnosed and the increase over time in this testing probability *incr_test_rate_sympt*. Like all other parameters mentioned in this section this is sampled at the start of each model run (see Table S27 below).

10 Modelling the effect of ART

Here we describe details of the modelling in relation to drug resistance and the effect of ART as well as pregnancy. Before giving full details we show (Figure S4) outputs of the model relating to outcomes by 1, 3 and 10 years from initiation of first line ART with either an efavirenz, atazanavir or dolutegravir based regimen (each with tenofovir and 3TC) in the absence of any switching in drug regimen. This illustrates the combined effects of the model assumptions which are described below. This is in the context of adherence profile B (see below for different adherence profiles considered), and it is for a situation with which there is no pre-ART NNRTI resistance.

Figure S4. Illustration of assumptions on effectiveness of efaviren and dolutegravir-containing 1st line regimens. Outcomes at 1, 3 and 10 years in absence of any switching to second line

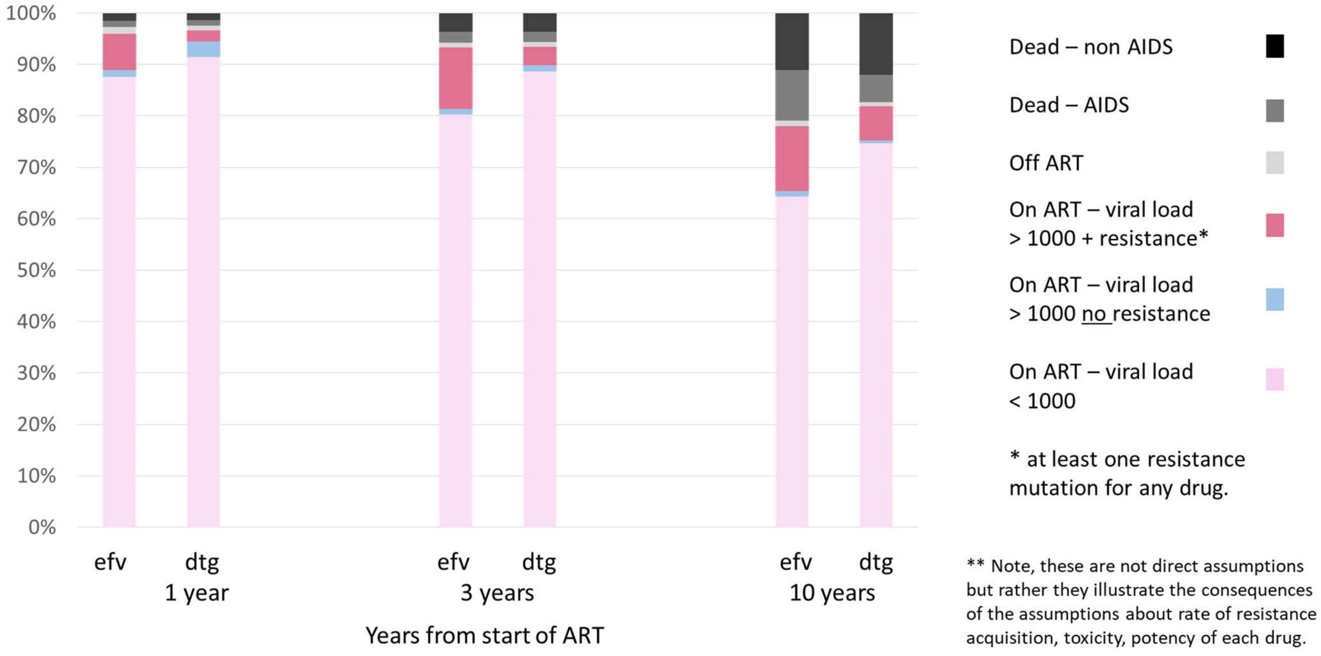

Throughout the sections below we introduce parameters which are indicated in italics. For those parameters for which a value is sampled the distribution is indicated at the end of this document.

10.1 Modelling the effect of ART

The structure of how the relationship between ART adherence, viral load, development of resistance, CD4 count and risk of death is modelled is illustrated in Figure S5 below. The adherence level - the determination of which is described in detail below - influences the risk of acquisition of new mutations as well as having a direct effect on the viral load and CD4 count. Acquisition of resistance mutations impacts on the total activity level of the regimen, calculated as the sum of the activity level of the drugs, akin to what is sometimes referred as a "genotypic sensitivity score". This, in turn, is a further determinant of the risk of new mutations arising. Distinction is made for each resistance mutation as to whether it is only present in minority virus (which can occur if the patient has a mutation present but is not taking a drug that selects for that mutation), so the mutation is assumed not transmissible, or if it is present in majority virus. Failure of the current line of ART is determined by CD4 count or viral load or clinical disease, depending on the monitoring strategy being implemented (in the current paper we assumed viral load monitoring from 2016, but with various levels of implementation), and this triggers a switch to the next line of ART (if assumed available, and often with a delay). The following sections provide further details, including how adherence levels are determined and how they influence the viral load, risk of resistance and the CD4 count. We also explain the modelling of ART interruption and loss to follow-up. We provide references to papers that have been used to inform the approach. It should be noted though that parameter values used in the model are rarely extracted directly from any one paper, they are values that are arrived at based on their ability to generally reproduce outputs that are consistent with observed estimates, as illustrated below.

Figure S5. Overview of the modelling of the effect of ART, highlighting the role of adherence.

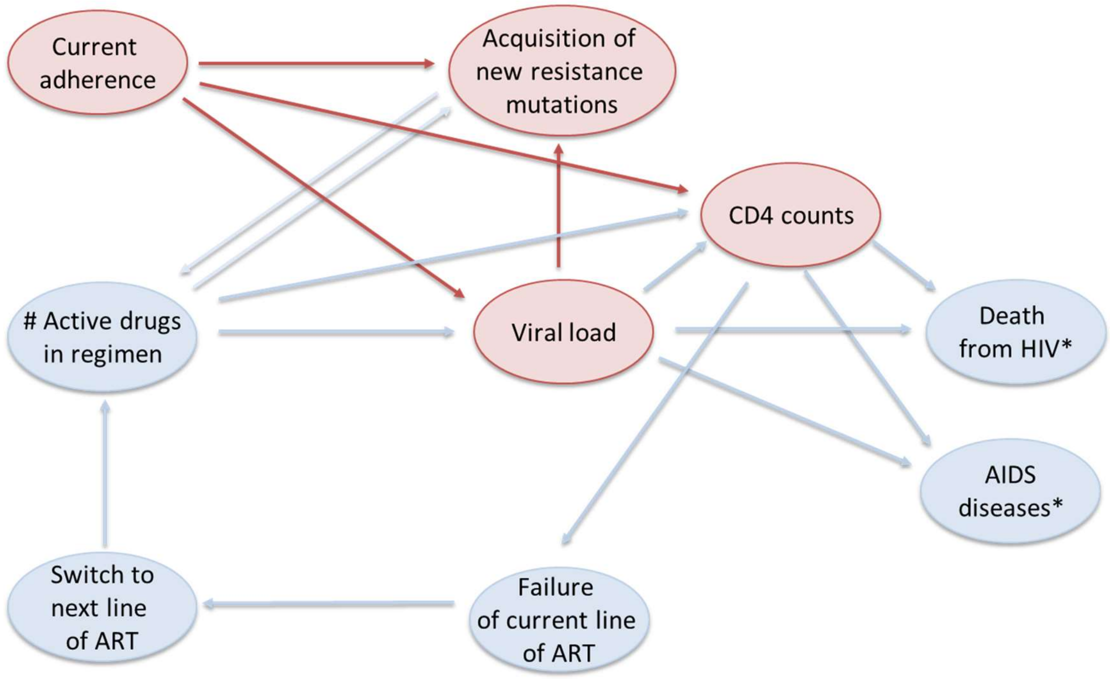

*influenced by age and PCP prophylaxis also

10.2 Initiation of ART

It is assumed ART became available in 2004. Eligibility for ART initiation in people diagnosed with HIV before 2003 is determined by the development of a WHO 4 or TB event. From 2004 to 2010, eligibility for ART initiation is determined by a measured CD4 count < 200 (in the last year) or the development of a WHO 4 event or TB and from 2011 to 2014 by a CD4 count <350 or a WHO 4 event. From 2011 onwards, pregnancy (option B+) is also an indicator for ART initiation. From 2014, ART initiation was indicated also based on a CD4 count < 500. From 2017 onwards, all people diagnosed with HIV are eligible for treatment. For people that are eligible to be initiated on treatment the probability that ART initiation occurs is determined by sampling from a Uniform (0,1) distribution and determining whether this is below the value for *pr_art_init*.

10.3 Switch to second line after failure of first line ART

The probability of switching per 3 month period after the criterion for failure of first line ART is met is *pr_switch_line*. The switch rate is likely to vary substantially by setting. (45-46)

10.4 Adherence pattern

The model specifies a current adherence level (i.e. for the current 3 month period) for people on ART, a value between 0%-100%. We first give a brief description of the approach and then give further detail. Since the model updates in 3 month periods, short term interruptions of days or a few weeks are treated as sub-optimal average adherence during the 3 month period. Interruption of ART over periods of 3 months or greater are referred to as ART interruption/discontinuation and modelled explicitly. ART interruption/discontinuation is usually concomitant with disengagement from clinic attendance. Average adherence in each 3 month period for an individual is determined from the underlying tendency to adhere (which is a lifelong value for the individual, unless changed as a result of an adherence intervention) with within-person period-to-period variability. Each patient thus has a certain higher or lower tendency to adhere but their actual adherence varies over time, both at random and according to factors such as age, gender, presence of symptoms and experiencing an enhanced adherence intervention as a result of a viral load measured > 1000 copies/mL, as detailed below. Effects of adherence on viral load and resistance acquisition risk are modelled by classifying levels into < 50%, 50-79%, $\geq$ 80%, with effects of ART on viral load suppression being greater the higher the adherence level and the resistance acquisition risk being highest in the 50%-79% category. We do not distinguish between patterns of adherence at a level more granular than the 3 monthly average level and hence cannot explicitly take into account the specific pattern within the 3 month period, which could be important (e.g. whether 80% adherence consists of missing drug one day in every five or a 1 week interruption in every 5 weeks). Thus the adherence level in each period should be conceived of as conveying the degree to which the pattern of adherence means that drug levels are maintained at intended therapeutic levels, rather than simply the average adherence over the period. The distribution of adherence levels was primarily determined by the adherence levels required for the model outputs to mimic observed data. This includes data on rates of resistance development and virologic failure and also data on the proportion of patients at first virologic failure who have no resistance mutations present (47-62)

Consistent with evidence that people tend to have different tendencies to adhere, adherence is modelled using two components. Each patient has a certain greater or lesser tendency to adhere (*adhav,* measured on a scale of 0-100%) but, as described above, their actual adherence in a given period varies over time. Adherence in a given 3 month period is referred to as *adh{t}*. *adhvar* is the standard deviation representing the within-person period-to-period variability over time. Thus, adherence at any one period is initially determined as follows (although with modifications explained below):- adh(t) = *adhav* + Normal(0,$adhvar^2$). An example of how the the distribution of the values of *adhav* and *adhvar* are specified as follows and as illustrated in Figure S6. We consider a range of such patterns and sample at random from the distribution of *adh_pattern*. The different adherence profiles from which we sample are as follows:

A 100

1% probability	*adhav* = 10% *adhvar* = 20%
1% probability	*adhav* = 79% *adhvar* = 20%
18% probability	*adhav* = 95% *adhvar* = 5%
80% probability	*adhav* = 95% *adhvar* = 2%

B 99

3% probability	*adhav* = 10% *adhvar* = 20%
2% probability	*adhav* = 79% *adhvar* = 20%
15% probability	*adhav* = 95% *adhvar* = 5%
80% probability	*adhav* = 95% *adhvar* = 2%

C 1

3% probability	*adhav* = 10% *adhvar* = 20%
3% probability	*adhav* = 79% *adhvar* = 20%
14% probability	*adhav* = 90% *adhvar* = 6%
80% probability	*adhav* = 95% *adhvar* = 5%

D 105

5% probability	*adhav* = 10% *adhvar* = 20%
7% probability	*adhav* = 79% *adhvar* = 20%
8% probability	*adhav* = 90% *adhvar* = 6%
80% probability	*adhav* = 95% *adhvar* = 5%

E 98

5% probability	*adhav* = 10% *adhvar* = 20%
10% probability	*adhav* = 79% *adhvar* = 20%
85% probability	*adhav* = 95% *adhvar* = 2%

F 2

5% probability	*adhav* = 10% *adhvar* = 20%
10% probability	*adhav* = 79% *adhvar* = 20%
27% probability	*adhav* = 90% *adhvar* = 6%
38% probability	*adhav* = 90% *adhvar* = 5%
20% probability	*adhav* = 95% *adhvar* = 5%

G 3

15% probability	*adhav* = 10% *adhvar* = 20%
15% probability	*adhav* = 70% *adhvar* = 20%
50% probability	*adhav* = 90% *adhvar* = 6%
20% probability	*adhav* = 95% *adhvar* = 5%

H 4

20% probability	*adhav* = 10% *adhvar* = 20%
20% probability	*adhav* = 79% *adhvar* = 20%
40% probability	*adhav* = 90% *adhvar* = 6%
20% probability	*adhav* = 95% *adhvar* = 5%

I 5

30% probability	*adhav* = 10%	*adhvar* = 20%
30% probability	*adhav* = 60%	*adhvar* = 20%
10% probability	*adhav* = 70%	*adhvar* = 6%
30% probability	*adhav* = 90%	*adhvar* = 5%

Figure S6. Illustration of adherence pattern assumptions. This is for adherence pattern F. 5% of the population have the adherence as shown in the top left, 10% as shown in the top right, etc. While adherence is generally high in the majority of people on ART (hence the high proportion of people on ART with viral suppression), most probably experience at least some periods of poorer adherence (e.g 63)

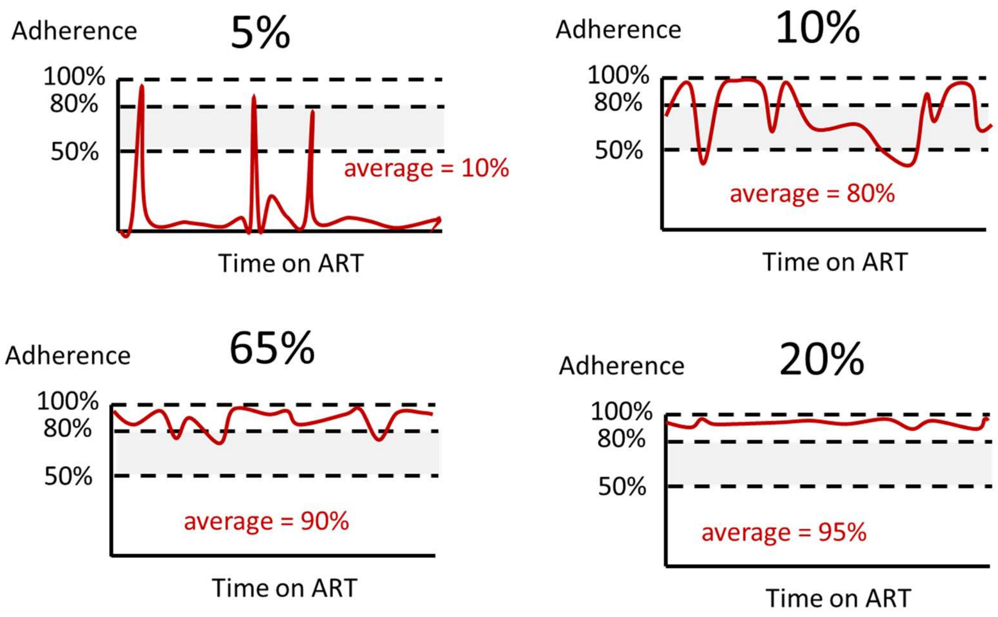

The above describes how we determine the person-specific underlying adherence (*adhav*) and variation in adherence (*advar*). *Adhav* remains constant over a person's lifetime, with the exception that it can increase as a result of an **adherence intervention due to viral load measurement above 1000 cps/mL** (see below), or **when starting 2nd line ART** (due to the fact that there is the emphasis that this is likely the last line of ART available – a last chance to be adherent, albeit that this effect can be counteracted by the lower adherence described below due to toxicity of atazanavir). The increase in *adhav* when starting second line is person-specific and given by 5% x exp(Normal(0, 1)).

Given *adhav* and *adhvar*, the adherence in any one period is determined as follows. First adh(t) = *adhav* + Normal(0,$adhvar^2$). Then adh{t} can be modified according to presence of a drug toxicity, a current WHO stage 4 condition or TB, or gender and age.

10.5 Effect of current drug toxicity and current TB or WHO stage 4 condition on adherence

The effect of drug toxicity on adherence is person-specific and given by 5% x exp(Normal(0, 0.3)). In any one 3 month period in which a toxicity is present there is 30% chance of this effect operating. The effect of drug toxicity on adherence is most clearly seen in randomized double-blind trials in which, for example, discontinuation rates are higher for efavirenz-based 1st line compared with a dolutegravir-based regimen (64, 65). During the time of a WHO stage 4 or TB disease, adherence is assume to be reduced by 10%..

10.6 Effect of age and gender on adherence

There is an effect of age on adherence, partly evidenced by differences in viral suppression levels, most notably with lower adherence in the 15-20 year age group compared with older ages (66-71). Gender is also an influence, as suggested by the higher proportion of women on ART with viral suppression in the PHIA surveys (72).

For men age 15-19 / 20-24 / 25-29, if initially there is adh{t} in a period above 80% there is a 30% / 20% / 10% chance of the adherence being reduced: 65% in two thirds of men and 10% in one third.

For women age 15-19, if initially there is adh{t} in a period above 80% there is a 20% chance of the adherence being reduced: 65% in two thirds of men and 10% in one third.
For women age 20-24 / 25-29 / 30-34 / 35--49 / 50+, if initially there is adh{t} in a period below 80% there is a 10% / 30% / 50% / 80% / 90% chance of the adherence being increased to 90%.

Comparisons between model outputs and data from the literature in Figure S7-S13 illustrate the extent to which the model captures various aspects of virologic responses to ART (efavirenz based regimens).

Figure S7. Risk of virologic failure while on ART according to adherence level

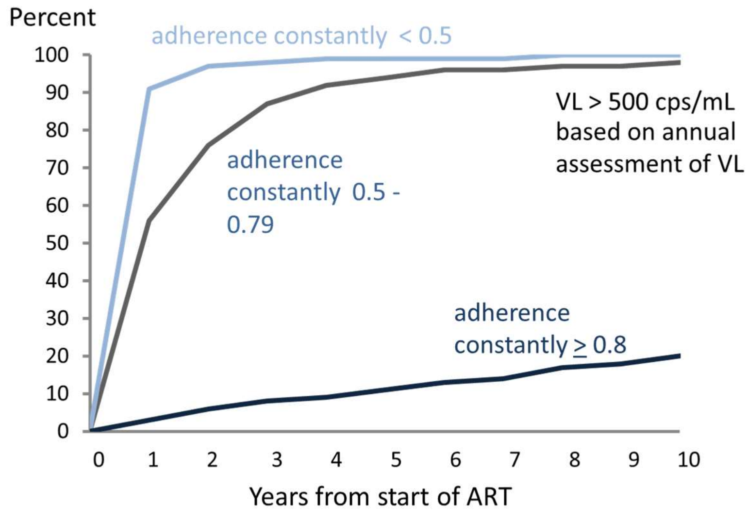

Figure S8. Risk of NNRTI resistance with virologic failure while on ART, according to adherence level

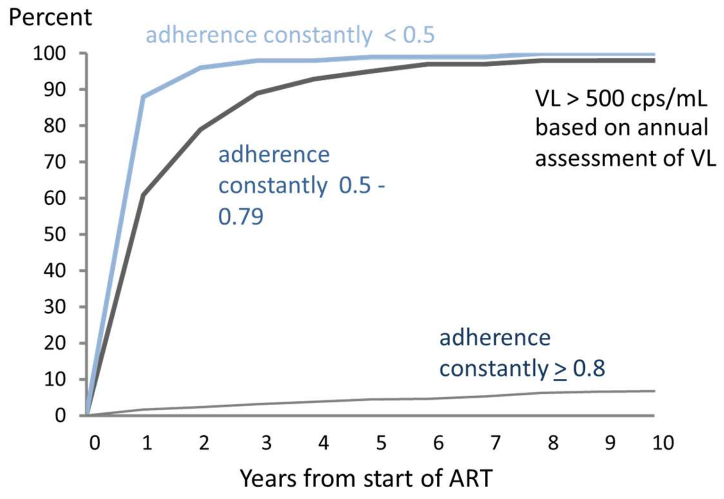

The distribution of adherence over the first year of ART has been compared with data from a large programme in Zambia (see Figure S9; (73)). Viral load suppression at one year from start of ART is shown in Figure S10. These are reconstructed outcomes for all people who have initiated ART in Zimbabwe (the overall mean CD4 count at initiation is 145 /mm^3). Figure S11 and Figure S12 compare Kaplan-Meier estimates of time to virologic failure and resistance, respectively, between the model and observed data, in the latter case from the UK due to the lack of data from sub-Saharan Africa (although noting that a substantial minority of people in the UK database originate from sub-Saharan Africa). Figure S13 illustrates the proportion of people with resistance (amongst those on ART with non-suppressed viral load) and corresponds to estimates from the large WHO resistance surveillance.

Figure S9. Distribution of average adherence level over first year of ART (for those on ART at 1 year) (73)**.**

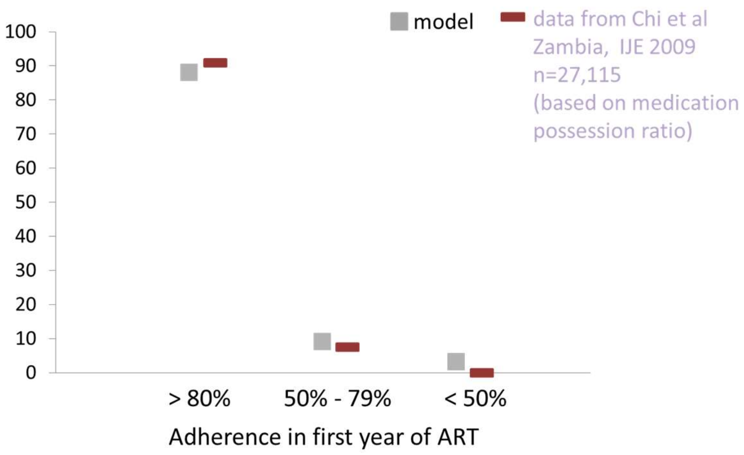

Figure S10. (a) Percent of people alive at given time points from start of ART who have viral load suppression and (b) percent of people alive and on ART at given time points from start of ART who have viral load suppression (74).

(a)

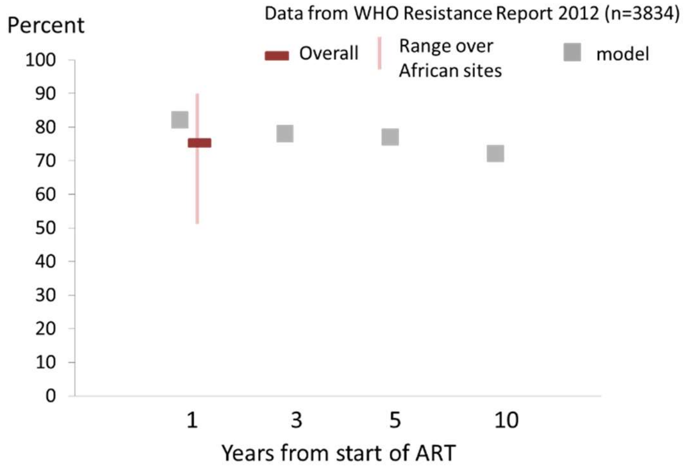

(b)

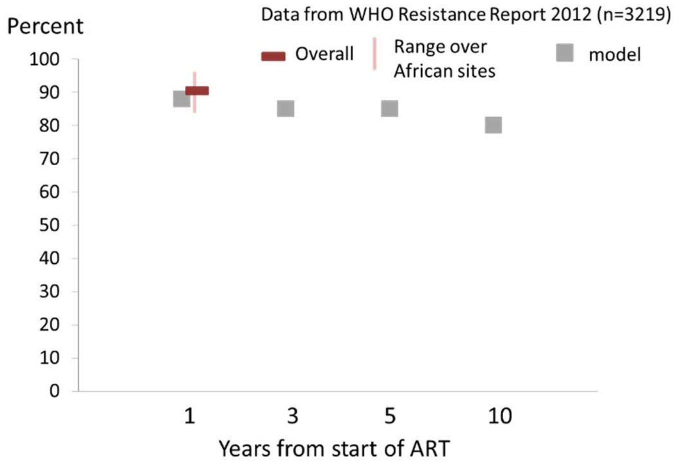

Figure S11 Kaplan Meier estimates of risk of virologic failure while on ART, by time from start of ART (45).

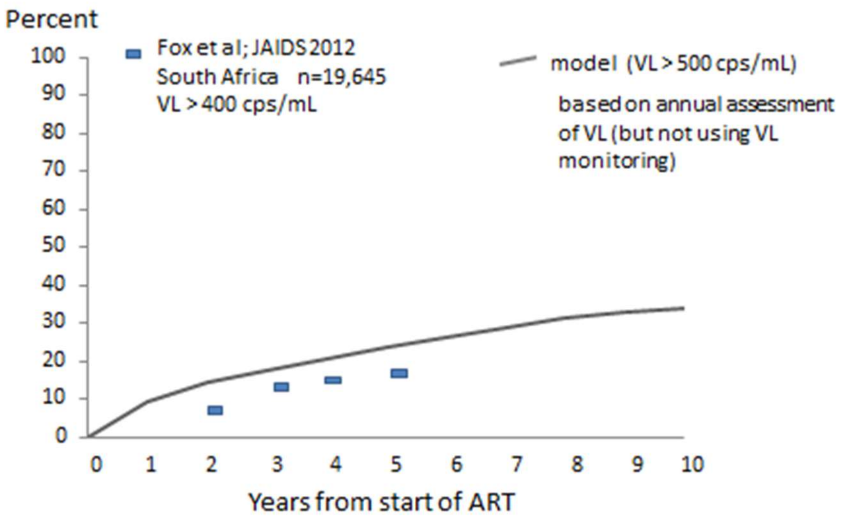

Figure S12. Kaplan Meier estimates of risk of NNRTI resistance with virologic failure while on ART, by time from start of ART (75).

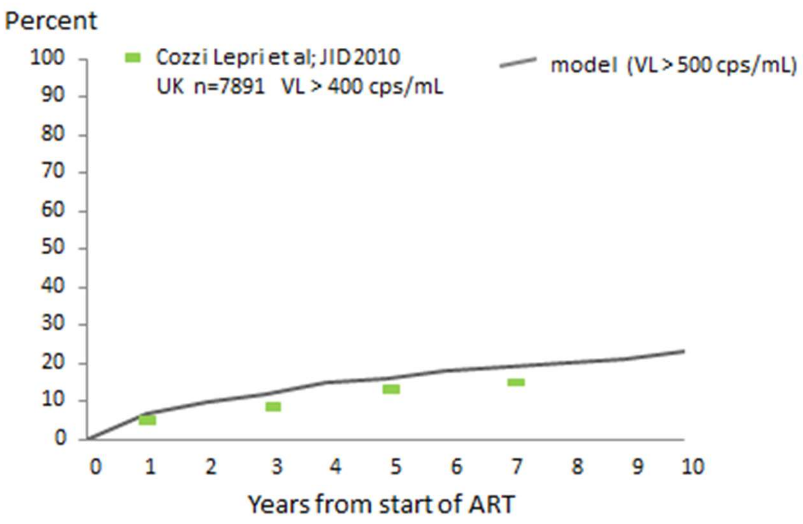

Figure S13. Of people with viral load > 500 at 1 year from start of ART, percent who have NNRTI drug resistance (74).

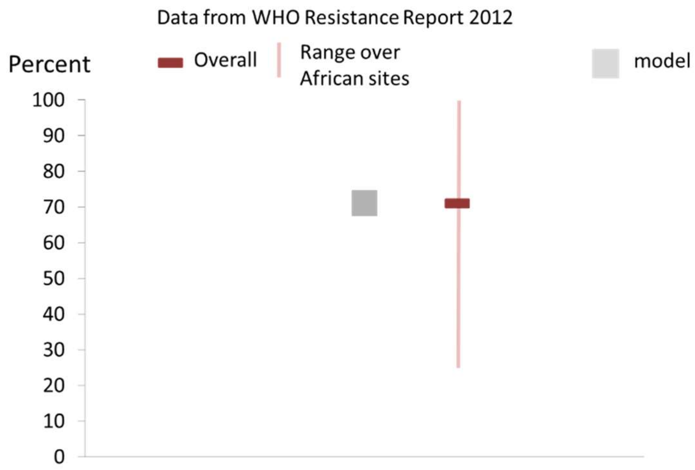

10.7 Effective adherence

We also considered the concept of *effective* adherence, which reflects predicted adequacy of drug levels, whereby for those on regimens that do not include an NNRTI the effective adherence is as the adherence itself, but for those on NNRTI-containing regimens the effective adherence is the adherence + *add_eff_adh_nnrti* (base value Log normal(ln 0.10, 0.30)), reflecting the long half life of NNRTI drugs (76) which is an advantage as it means such regimens are more forgiving of periods of poor adherence (48-50, 55, 77-80). Additionally,-it is assumed that patients on ART are susceptible to occasional (rate 0.02 per 3-months severe temporary drops in drug level (i.e. effective adherence level), leaving them susceptible to viral rebound (but with low risk of resistance as the effective adherence drop is so profound). This phenomenon is assumed to be 100 times more frequent among those on protease inhibitor regimens than in those on other regimens. This latter assumption is the only plausible means (at least within our model framework) to explain why virologic failure occurring on boosted protease inhibitor regimens often occurs in the absence of resistance (81).

10.8 Effect of viral load measurement above 1000 cps/mL on adherence

As mentioned, adherence can be affected by experience of an enhanced adherence intervention after initial measurement of viral load > 1000 copies/mL which is assumed to lead to an increase in adherence in 70% of people, consistent with data showing that a significant proportion of people with measured viral load > 1000 copies/mL who undergo an adherence intervention subsequently achieve viral suppression without a change in ART (53, 54, 82, 83) and broadly consistent with a meta-analysis (84). Although the appropriate duration to assume for this effect is uncertain (54), the impact of adherence interventions has often been shown to diminish with time (85). Based on this overall body of data, we assume that the adherence intervention is effective only the first time it is performed and that for 40% the effect is permanent (i.e. 70% x 40%= 28% of those with a viral load >1000; in this case the value of *adhav* is reduced from this point), but that in the remaining 60% (i.e. 70% x 60% = 42% of those with viral load>1000) it lasts only 6 months.

10.9 ART interruption / discontinuation

People can interrupt ART, and this may be due to not continuing with clinic visits (disengagement, modelled as simultaneous interruption and loss to clinic follow up) but ART can be interrupted also in those still attending clinical visits. The basic rate of interruption due to patient factors (refered to as *rate_int_choice*, although recognising that this is often not a free choice) is greater in people with current toxicity (2-fold) and those with a greater tendency to be non-adherent (1.5-fold if adherence average *adhav* 50 – 79% and 2-fold if adherence average *adhav* < 50%). In a systematic review, drug toxicity, adverse events and side effects have been found to be the most commonly given reasons for drug discontinuation (86). The rate of interruption also reduces with time on ART, decreasing after 1 years (87-89). If adherence average (*adhav*) $\geq$ 80% then the chance that interruption coincides with interrupting/stopping visits to the clinic is equal to *prob_lost_art*; if 50 <= *adhav* < 80% then *prob_lost_art* is multiplied by 1.5, if *adhav* < 50% then *prob_lost_art* is multiplied by 2. This is due to an assumption that factors leading to poor adherence are also likely to be associated with interruption. The rate of interruption and disengagement with care is likely to vary by setting. Interruption of ART is assumed close to zero during pregnancy. In addition, in 20% of setting scenarios there is an effect such that those with recent non-primary condomless sex partners have a 1.5 fold higher risk of interruption. Figure S14 shows a comparison between modelled and observed (from a study by Kranzer et al (87). Kaplan Meier estimates of the percent of people having interrupted or discontinued ART by time from ART initiation.

Figure S14. Percent who have interrupted or discontinued ART by time from initiation (87).

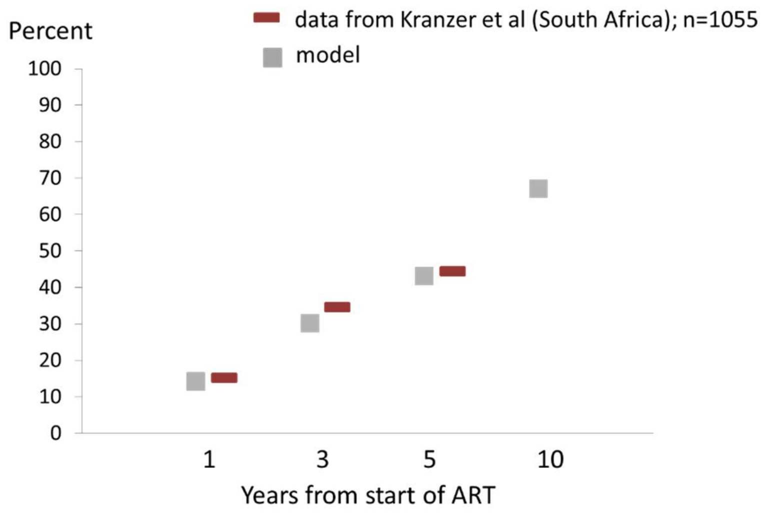

10.10 Interruption of ART without clinic/clinician being aware

It is known that in some instances people on ART have such poor adherence that they have in fact interrupted or stopped ART entirely but, in the same way that the clinic is not always aware of the true adherence level, they are also not always aware when the person has completely interrupted ART. This means that the clinic may think a patient is virologically failing, because viral load is high, when in fact this is due to interruption rather than resistance. This can be seen from studies on people with virologic failure in which a proportion have no identified resistance mutations (51, 53, 90). Thus, when a person interrupts ART (but remains under care) we introduce a variable that indicates whether the clinic is unaware. *clinic_not_aw_int_frac* (base value Beta (6,4), median=0.61). This distribution was chosen to produce realistic model outputs for the proportion of people with virological failure who have resistance. If a patient has interrupted ART with the clinic unaware then not only is the patient (wrongly) classified (by the clinic) as virologically failing (if viral load has been measured), but a switch to second line can occur. Figure S15 compares the proportion of people with resistance between our model and WHO survey data.

10.11 Re-initiation of ART after interrupting in patients still under clinic follow-up

For patients who have interrupted ART due to choice but are still under clinic follow-up, the probability of restarting ART per 3 months in the base model is *rate_restart*. This probability is increased 3-fold if a new WHO 3 condition has occurred at t-1, and 5-fold if a new WHO 4 condition has occurred at t-1 since occurrence of clinical disease in a person seen at clinic is likely to prompt ART re-initiation. This will vary by setting but is informed by studies showing that of people who have initiated ART who are still seen at clinic a very high proportion are on ART at 12 months from start of ART (91). Kranzer et al found a rate of restarting ART amongst those that interrupted or discontinued of 21 per 100 person-years but this figure is an overall figure which includes in the denominator those who are not attending the clinic (loss to follow-up and return to care are described below) (87). The equivalent figure, produced as an output from the model is 19 per 100 person-years.

10.12 Interruption due to drug stock-outs

The basic rate of interruption due to interruption of the drug supply is *prob_supply_interrupted* per 3 months. This will vary over time and by setting but we assume low rates in current and future years (0.003 per 3 months per person). For patients who have interrupted ART due to interruption of supply the probability of restarting ART per 3 months is *prob_supply_resumed* (91).

10.13 Loss to follow-up while off ART (for reasons apart from drug stock-outs)

The probability per 3 months of interrupting/stopping clinic visits (i.e. being lost to follow-up) is *rate_lost* if adherence average *adhav* ≥ 80%. This is increased by 1.5 fold if 50% ≤ *adhav* < 80% and by 2-fold if *adhav* < 50%. This high rate is informed by the fact that low numbers of people attending clinics after having been initiated on ART are not still on ART (e.g. 74). Interruption of ART and loss to follow-up are assumed correlated with the underlying tendency to adhere when on ART because we assume that the same underlying social, practical and economic factors will be an underlying cause of these behaviours.

For people lost to follow-up who are asymptomatic, the probability of returning to clinic per 3 months is *rate_return* if adherence average *adhav* $\geq$ 80%. This is decreased by 2-fold if 50% $\leq$ *adhav* < 80% and by 3-fold if *adhav* < 50%. If a person develops a new WHO 3 or 4 event then they are assumed to return to the clinic with probability 1. As mentioned above, this leads to an overall rate of restarting of ART after interruption (including having been loss to follow-up in many cases) consistent with the estimates from South Africa from Kranzer et al, although these will vary by setting (45, 78, 92).

Figure S15. Status at 1 year from start of ART. Data is from WHO Drug Resistance Surveillance Report (2012) (74).

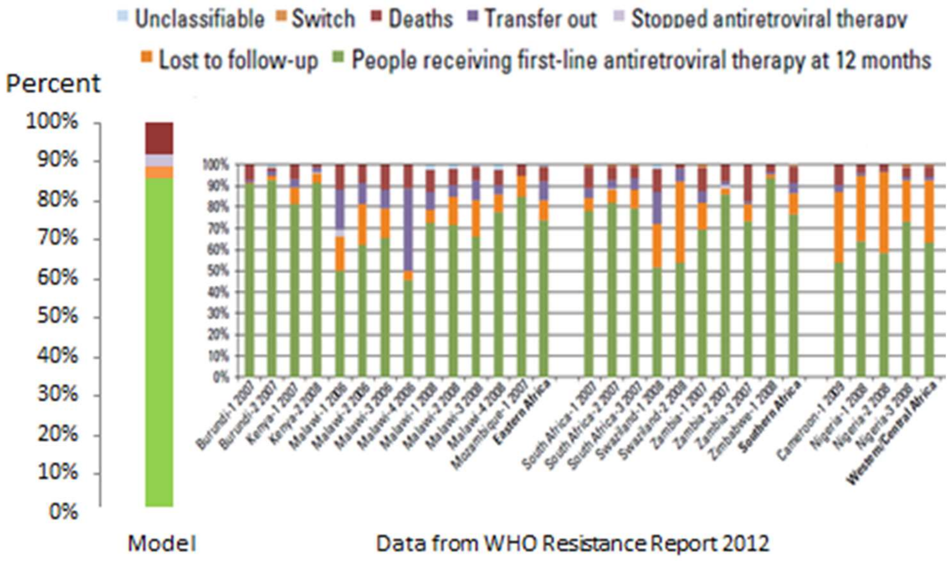

10.14 Effect of ART on viral load, CD4 count, resistance development and drug toxicity

This section describes the determination of updated viral load, CD4 count, and acquisition of new resistance mutations in a given time period for people on ART. The updated viral load, CD4 count and risk of new resistance mutations appearing all depend on the effective adherence in the previous and current period, the number of active drugs (*nactive(t-1)*) and the current viral load, as well as the time period from the last time ART was started or restarted. The values of viral load, CD4 count, and resistance mutation risk for any combination of these factors are given in Table S19-S21 below. The rationale behind this approach and how the specific values in the table were chosen is explained below. The choice of values is directly informed by studies in this area and by comparison of model outputs with data. For the new resistance mutation risk, the number in the table is multiplied by the viral load (mean of values at t-1 and t) to give a value for the variable *newmut*, which is used when assessing whether a new mutation or mutations have arisen (see below).

10.15 Number of active drugs

We use the concept of the number of drugs that are active, based on presence of resistance mutations to the drugs being used. The level of resistance is determined by the presence of drug resistance mutations, with a given set of mutations being translated into a level of resistance to a given drug on a scale of 0 to 1 in the same way as is done for common resistance interpretation systems. The activity level of a drug is then calculated as 1 minus the level of resistance to the drug. The ability of the number of active drugs, or the genotypic sensitivity score, to predict the viral load outcome is well established (93), and the concept of using a genotypic score to define "optimised background therapy" has been common to the design of several trials in treatment experienced patients (e.g. 94). This is the basic concept but note that below we explain consider that drugs, such as boosted PIs, can have higher potency (since they can virtually sustain viral suppression alone) and thus contribute a value greater than 1.

10.16 Classification of adherence levels

While we model the adherence level for each individual at each three month time period as a value between 0 and 100%, to determine the viral load, CD4 count and resistance risk, as noted above, we classify adherence into three levels. This is the simplest approach that allows inclusion of the fact that the relationship between adherence and resistance risk is not linear, since the risk of resistance tends to be lower when the adherence is either low or high, and the risk of resistance is highest when adherence is moderate, allowing enough replication for mutations to be selected for and enough drug present to allow selection of virus with resistance mutations (48, 77, 95).

As mentioned, the cut-offs used to define the three adherence levels are 50% and 80%. Adherence-resistance and adherence-viral load relationships differ by regimen type and even specific regimen within a class and any overall breakdown into groups is necessarily a simplification. A cut off of 80% is chosen as the upper level as (unlike for unboosted PI regimens) at adherence levels of at least 80%, NNRTI and boosted PI regimens are likely to have maximal or close to maximal effects on viral load and minimal risk of resistance selection (80). Actual risk of resistance probably depends on the pattern of adherence, not just the average over a three month period, so that a treatment interruption of over 1 week during the three month period, while maintaining an overall average adherence of 80%, could lead to a higher level of risk of resistance emergence than a situation in which the adherence was more uniform over the period (96), although in people who have ongoing viral suppression NNRTI regimens seem to be generally robust to even relatively low levels of adherence (78-80, 97). A level below 50% is one that that has been associated with raised risk of detectable viral load (96, 98)

10.17 Determination of viral load, CD4 count and risk of resistance in people on ART

Viral load, CD4 count and risk of resistance in the first 3 months after (re-)starting ART

Table S19 shows how the viral load, CD4 count and risk of resistance is determined for people in the first 3 months after starting ART or re-starting ART after an interruption of at least 3 months. Since in this early period on ART, the viral load will depend on the initial value the updated viral load is given as a reduction from the pre-ART maximum viral load. If the number of active drugs is three or more then at a high adherence level (above 0.8) the mean viral load change from the pre-ART maximum is

3 log copies/mL. To reflect the fact that there is variability in the response (99), the value for a given person is sampled from a Normal distribution with standard deviation 0.5. This viral load response diminishes both with decreasing number of active drugs in the regimen being started (which is informed by data from studies relating GSS to virologic outcome, as well as by studies of mono and dual therapy regimens (93, 100-106). The viral load response also diminishes with decreasing level of adherence (see Figure 16 and for example Genberg et al (106). As is well established, the CD4 count response generally mirrors the viral load response, although with very low numbers of active drugs and low adherence there is a mean decrease in CD4 count and still a small decrease in viral load from the maximum. Note that we do not incorporate the known more rapid decline in viral load seen with integrase inhibitors.

Figure S16. Model output: of people on ART, percent with current VL >500 according to current adherence. Comparison with data from Genberg at el on electronic monitoring-based adherence measures (106).

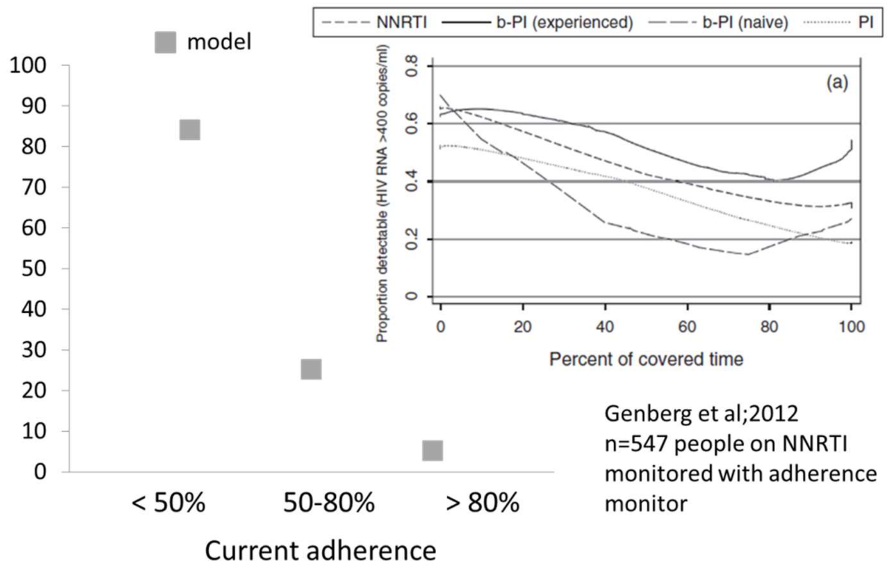

Regarding the risk of new drug resistant mutations arising, Tables S19-S21 provide a number for “new mutation risk” that is multiplied by the viral load (mean of values at t-1 and t) to give a probability used when assessing whether a new mutation(s) has/have arisen. Values of the new mutations risk have been chosen in conjunction with the translation of presence of mutations into reduced drug activity to provide estimates of resistance accumulation consistent with those observed in clinical practice (62, 107-113).

Risk of new resistance mutations arising increases with decreasing number of active drugs, reflecting the known greater risk of resistance with regimens less able to suppress viral replication, most clearly seen in the fact that early mono and dual therapy regimens were highly susceptible to resistance development (92-94). At low adherence levels, the risk of resistance development is generally low regardless of the number of active drugs, as drug selection pressure is low. However, for those on NNRTI regimens the new resistance mutation risk is assumed to be that for the effective adherence category of 50 – 80% (i.e. maximal) even if the effective adherence is below 50%, reflecting the fact that NNRTI resistance develops easily, even when drug exposure is very low (48, 49).

Viral load, CD4 count and risk of resistance between 3-6 months from (re-)starting ART

For the period 3-6 months from (re-)start of ART (Table S20; to reduce the table content we do not provide the matrices of values for the resistance risk or CD4 count, only for the viral load (the full table is available in Cambiano et al 2014 (114). We consider the adherence in both the current and previous 3 month period, since the likelihood of reaching viral suppression by 6 months will depend on adherence throughout the whole 6 month period from start of ART, although the adherence in the current period is assumed to be the stronger factor. By 6 months after starting ART, those on 3 or more active drugs with consistently high adherence generally reach a relatively high level of viral suppression, regardless of pre-ART maximal viral load, so a person's viral load is no longer given by the change from baseline but the absolute level of viral load which it is likely they have reached. In these optimal conditions of high adherence and maximal active drugs we assume the viral load has a mean value of 0.5 log, again with variability between individuals. Since most viral load assays have a lower limit of quantification of 40 or 50 copies per mL, it is not actually known what the actual viral load level is, although highly sensitive assays suggest that a proportion of patients reach below 11 copies/mL (115). At lower numbers of active drugs and lower adherence, the viral load is still related to the maximal pre-ART viral load rather than being an absolute value, as the person's viral load has not become so low that the initial value loses relevance. The viral load response decreases with a lower number of active drugs, lower current adherence, and lower adherence in the previous 3 month period. Values for the viral load response between those known from studies (high level of suppression for 3 active drugs and maximal adherence, and only around 0.5 log viral suppression when adherence is < 0.5 even with three active drugs (104, 116) are imputed assuming a monotonic relationship. CD4 count responses again mirror the viral load response, as has been extensively studied in patients with ongoing viraemia on ART (117). Risk of new resistance mutations again increases with decreasing number of active drugs, if current adherence is in the middle or highest group. The only situation in which risk of new mutations is extremely low is when the number of active drugs is 3 or close to 3 and the current adherence is in the high category.

Viral load, CD4 count and risk of resistance after 6 months of (re-)starting ART

Table S21 shows how the viral load, CD4 count and risk of resistance is determined for the situation where a person has been on ART for more than 6 months and the viral load is suppressed or partially suppressed (< 4 log copies/mL). These values are similar to those used for the period 3-6 months from start of ART except that there is assumed to dependence on the adherence in the current 3 month period only.

The situation where the viral load is above 4 log copies /mL, 10,000 copies/mL is treated the same as that in the period 3-6 months from start of ART (described above), with adherence in the current and previous period having some influence.

10.18 Variable patient-specific tendency for CD4 count rise on ART

There is variability in the tendency for the CD4 count to rise on ART, for a given level of viral load suppression. For scenarios in the above Tables (S19 – S21) in which the CD4 count change is positive

the CD4 count change is multiplied by this patient-specific factor (i.e. it is fixed for each patient), which is given by sampling for each patient from Exp (N(0, (*sd_patient_cd4_rise_art*)^2) where sd_patient_cd4_rise_art = 0.2. To reflect the fact that the rate of CD4 count increase on ART tends to diminish with time, for those with patient-specific factor determining the CD4 count rise on ART > 1, this factor is divided by 1.25 after 1 year of continuous treatment, by 3 after 3 years of continuous treatment and by 10 after 5 years. In addition there is a dependence of CD4 increase on current CD4 level with a multiplying factor of 0.85-fold if the CD4 count is 100-200 and 0.7-fold if > 200. Comparisons of resultant outputs with observed data are described below.

10.19 Accelerated rate of CD4 count loss if PI not present in regimen

The rate of change in CD4 count in people on failing regimens is largely based on data from the PLATO collaboration, for which patients were mainly on regimens containing a PI (117). If the regimen does not contain a PI the change in CD4 count per 3 months is modified (in the base model) by *poorer_cd4_rise_on_failing_nnrti* (= -6 /mm^3). This applies regardless of viral load level, so PIs are assumed to lead to a more beneficial CD4 count change than NNRTIs (117). We assume in 50% of setting scenarios that this applies also for dolutegravir and in 50% that it does not (determined by *poorer_cd4_rise_on_fail_nn_ii*).

10.20 Variability in individual (underlying) CD4 counts for people on ART

Once the mean of the underlying CD4 count is obtained as described above for people on ART, to obtain the CD4 count, variability (*sd_cd4* = 1.2) is added on the square root scale. The estimate was based on unpublished analyses
The ongoing CD4 count increases in people with viral suppression are informed by Mocroft et al 2007 (118). CD4 count As a result of these assumptions, model outputs for mean changes in CD4 count from start of (efavirenz-based) ART in people with ongoing viral suppression at years 1, 3, 5, 10 are 200, 338, 442 and 633. Amongst people starting ART with CD4 < 100 at start of ART, the proportion of people with a CD4 count above 500/mm3 by 5 years from start of ART is 62%. Similar figures for people with baseline CD4 count 100-200, and 200-300 are 75% and 78% respectively. In comparison, Bishop et al report CD4 count changes of 206, 278, 419, 509 at 1, 3, 5 and 10 years respectively (119). Geng et al report a CD4 count increase of 365 /mm3 in people in southern Africa starting with a CD4 count of 150/mm3 (120). At 5 years from start of ART O'Connor et al report a proportion with CD4 count > 500 of 60% for people starting ART with CD4 count < 100 (121). In the Single trial the CD4 count rise at 1 year was 208/mm^3 and at 3 years was 332/mm^3 (64, 65).

Each person has a maximum attainable CD4 count given by sampling from exp(Normal(7.3, 0.25^2)).

Table S19. Viral load (mean change from viral load max), CD4 count change (mean change between t-1 and t), and new mutation risk in first 3 months. For 0 active drugs, these are the changes regardless of time from start of ART. For viral load this is the mean of a Normal distribution with standard deviation 0.2, from which the patient's value/change is sampled. For the CD4 count patients vary in their underlying propensity for CD4 rise on ART (given by sampling from lognormal(1,0.5^2) and the CD4 count change given here is multiplied by this factor. For the new mutation risk, this is a number that is multiplied by the viral load (mean of values at t-1 and t). The resulting probability is used when assessing whether a new mutation or mutations have arisen.

	Effective adherence between t-1 & t	Number of active drugs												
		3	2.75	2.5	2.25	2.0	1.75	1.5	1.25	1	0.75	0.5	0.25	0
Viral load	≥ 80%	-3.0	-2.6	-2.2	-1.8	-1.5	-1.25	-0.9	-0.8	-0.7	-0.55	-0.4	-0.3	-0.3
(log change	≥ 50%, <80%	-2.0	-1.6	-1.2	-1.1	-0.9	-0.8	-0.6	-0.5	-0.4	-0.25	-0.1	-0.05	-0.1
from vmax)	< 50%	-0.5	-0.4	-0.3	-0.25	-0.2	-0.15	0.0	+0.05	+0.1	+0.1	+0.1	+0.1	0.0
CD4 count	≥ 80%	+50	+45	+40	+35	+30	+25	+20	+17	+13	+10	+5	-2	-15
change	≥ 50%, <80%	+30	+30	+23	+20	+15	+13	+10	+8	+5	+3	0	-7	-17
(t-1 to t)	< 50%	+5	+4	+3	+2	+1	-1	-3	-6	-10	-11	-12	-13	-18
New mutation	≥ 80%	0.002	0.01	0.03	0.05	0.1	0.15	0.2	0.3	0.4	0.45	0.5	0.5	0.5
Risk	≥ 50%, <80%	0.15	0.15	0.2	0.25	0.3	0.3	0.3	0.35	0.4	0.45	0.5	0.5	0.5
(x log viral load)	< 50%*	0.15	0.15	0.2	0.25	0.3	0.3	0.3	0.35	0.4	0.45	0.5	0.5	0.5
	< 50%**	0.05	0.05	0.05	0.05	0.05	0.05	0.05	0.05	0.05	0.05	0.05	0.05	0.05

* for NNRTI containing regimen, ** for boosted PI containing regimen.

Table S20. Summary of viral load (mean absolute value or mean change from viral load max) between 3-6 months, and after 6 months if viral load at t-1 > 4 logs. This is the mean of a Normal distribution with standard deviation 0.2, from which the patient's value/change is sampled.

Effective adherence between t-2 & t-1	Effective adherence between t-1 & t	Number of active drugs											
		3	2.75	2.5	2.25	2.0	1.75	1.5	1.25	1	0.75	0.5	0.25
≥ 80%	≥ 80%	0.5	0.8	1.2	1.4	2.0	2.7	-1.7	-1.15	-0.9	-0.75	-0.6	-0.4
≥ 50%, <80%	≥ 80%	1.2	1.2	1.2	1.4	-2.0	-1.6	-1.2	-1.05	-0.9	-0.7	-0.5	-0.35
< 50%	≥ 80%	1.2	1.2	1.2	1.4	-2.0	-1.6	-1.2	-1.0	-0.9	-0.7	-0.5	-0.2
≥ 80%	≥ 50%, <80%	1.2	1.6	1.8	2.2	2.4	-2.4	-1.5	-0.9	-0.7	-0.55	-0.4	-0.3
≥ 50%, <80%	≥ 50%, <80%	2.5	2.5	2.5	2.5	-1.2	-1.1	-0.8	-0.65	-0.5	-0.35	-0.2	-0.05
< 50%	≥ 50%, <80%	-2.0	-1.8	-1.5	-1.35	-1.2	-1.1	-0.8	-0.65	-0.5	-0.2	-0.2	-0.05
≥ 80%	< 50%	-0.5	-0.4	-0.3	-0.25	-0.2	-0.15	-0.10	-0.05	+0.0	+0.0	+0.0	+0.0
≥ 50%, <80%	< 50%	-0.5	-0.4	-0.3	-0.25	-0.2	-0.15	-0.10	-0.05	+0.0	+0.0	+0.0	+0.0
< 50%	< 50%	-0.5	-0.4	-0.3	-0.25	-0.2	-0.15	-0.10	-0.05	+0.0	+0.0	+0.0	+0.0

Table S21. Summary of viral load (mean change from viral load max), CD4 count change (mean change between t-1 and t), and new mutation risk after 6 months, where viral load at t-1 < 4 logs. For viral load this is the mean of a Normal distribution with standard deviation 0.2, from which the patient's value/change is sampled. For the CD4 count patients vary in their underlying propensity for CD4 rise on ART (given by sampling from lognormal($1,0.5^2$) and the CD4 count change given here is multiplied by this factor. For the new mutation number, this is a number that is multiplied by the viral load (mean of values at t-1 and t). The resulting probability is used when assessing whether a new mutation or mutations have arisen.

	Effective adherence between t-1 & t	Number of active drugs											
		3	2.75	2.5	2.25	2.0	1.75	1.5	1.25	1	0.75	0.5	0.25
Viral load (absolute value or log change from vmax)	≥ 80%	0.5	0.0	1.2	1.6	-2.5	-2.0	-1.4	-1.15	-0.9	-0.75	-0.6	-0.3
	≥ 50%, <80%	1.2	1.2	1.2	1.4	-1.2	-1.0	-0.7	-0.6	-0.5	-0.4	-0.3	-0.1
	< 50%	-0.5	-0.4	-0.3	-0.25	-0.2	-0.2	-0.1	-0.1	-0.1	-0.1	-0.1	-0.0
CD4 count Change (t-1 to t)	≥ 80%	+30	+28	+25	+23	+21	+19	+3	-5	-9	-10.5	-12	-12
	≥ 50%, <80%	+15	+13	+10	+8	-4.5	-7.5	-10	-12	-13	-14	-15	-15
	< 50%	-13	-14	-15	-15.5	-16	-16.5	-17	-17	-18	-17	-17	-17
New mutation risk (x log viral load)	≥ 80%	0.002	0.01	0.03	0.08	0.10	0.15	0.2	0.3	0.4	0.45	0.5	0.5
	≥ 50%, <80%	0.15	0.18	0.2	0.25	0.3	0.3	0.3	0.35	0.4	0.45	0.5	0.5
	< 50%*	0.15	0.18	0.2	0.25	0.3	0.3	0.3	0.35	0.4	0.45	0.5	0.5
	< 50%**	0.05	0.05	0.05	0.05	0.05	0.05	0.05	0.05	0.05	0.05	0.05	0.05

* for NNRTI containing regimen, ** for boosted PI and dolutegravir containing regimen.

10.21 Viral load and CD4 count changes during ART interruption

Viral load returns to previous maximum viral load (vmax) in 3 months and adopts natural history changes thereafter.

CD4 rate of decline returns to natural history changes (ie those in ART naïve patients) after 9 months, unless the count remains > 200 above the CD4 nadir

Rate of CD4 count decline depends on current viral load. c(t) is the CD4 count at time t, cmin(t) is the CD4 count nadir measured by time t and cc(t-1) is the change in CD4 count from t-1 to t.

if time off ART = 3 months or if time off ART > 3 months and CD4 in previous period is > 300 above the minimum CD4 count to date

v(t) = vmax(t-1)
if v(t) $\geq$ 5 then cc(t-1) = Normal $(-200,10^2)$
if 4.5 <= v(t) < 5 then cc(t-1) = Normal $(-160,10^2)$
if v(t) < 4.5 then cc(t-1) = Normal $(-120,10^2)$

If this leads to c(t) < cmin(t) (CD4 nadir) then c(t) is set to cmin(t)
if time off ART = 6 months:-

if v(t) $\geq$ 5 then cc(t-1) = Normal $(-100,10^2)$
if 4.5 <= v(t) < 5 then cc(t-1) = Normal $(-90,10^2)$
if v(t) < 4.5 then cc(t-1) = Normal $(-80,10^2)$

if time off ART = 9 months:-

if v(t) $\geq$ 5 then cc(t-1) = Normal $(-80,10^2)$
if 4.5 <= v(t) < 5 then cc(t-1) = Normal $(-70,10^2)$
if v(t) < 4.5 then cc(t-1) = Normal $(-60,10^2)$

This is broadly based on evidence from a number of analyses of the effects of ART interruption (e.g. (122-125))

10.22 Incidence of new current toxicity and continuation of existing toxicity

Toxicities including gastrointestinal symptoms, rash, hepatoxicity, CNS toxicity, lipodystrophy, hypersensitvity reaction, peripheral neuropathy and nephrolithiasis can occur with certain probability on certain specific drugs (Table S22). These probabilities are based broadly on evidence from trials and cohort studies, although there are no common definitions for some conditions which complicates this.

Table S22. Risk of development of specific drug toxicities.

Toxicity	Drug	Risk of development per 3 months	Probability of continuation if pre-existing
Nausea	atazanavir	1% (5-fold higher in 1st year)	50%
	zidovudine	3% (5-fold higher in 1st year)	50%
Diarrhoea	atazanavir	1% (2.5-fold higher in 1st year)	50%
Rash	efavirenz	3% (in first 6 months on efavirenz)	
CNS toxicity	efavirenz	10% (if been on efavirenz <1 year)	80% if been on efavirenz <1 year. 90% if been on efavirenz ≥1 year
	dolutegravir	5% (if been on dolutegravir <1 year)	40% if been on dolutegravir <1 year. 90% if been on dolutegravir ≥1 year
Lipodystrophy	zidovudine	1.5%	100%
Anaemia	zidovudine	3% (1.5-fold higher in 1st year)	20%
Headache	zidovudine	10% (1.5-fold higher in 1st year)	40%
Lactic acidosis	zidovudine	0.02%	
Renal dysfunction	tenofovir	0.35%	100%

10.23 Switching of drugs due to toxicity

If toxicity is present then we consider in some scenarios that drugs may be switched due to toxicity.

10.24 Emergence of specific resistance mutations and their effect on drug activity

newmut (see Table S19 – S21 above) is a probability used to indicate the level of risk of new mutations arising in a given 3 month period. If this chance comes up in a given 3 month period (determined by sampling from the binomial distribution) then the following criteria operate.

Table S23. Risk of acquiring new resistance mutations.

Resistance mutation	Probability of arising	Conditions
M184	80%	if on 3TC or FTC
# TAMS increases by 1	20%	if on zidovudine and (not on 3TC nor FTC)
	12%	if on zidovudine and (on 3TC or FTC)
# TAMS increases by 2	1%	if on zidovudine and (not on 3TC nor FTC)
	1%	if on zidovudine and (on 3TC or FTC)
K65	10%	if on tenofovir

Q151	2%	if on zidovudine
K103	60%	If on efavirenz
Y181	10%	If on efavirenz
G190	10%	If on efavirenz
I50L	3%	If on atazanavir
I84V	3%	If on atazanavir
N88	3%	If on atazanavir
primary dolutegravir mutation	3%	if on dolutegravir
secondary dolutegravir mutation	3%	if on dolutegravir

These values are chosen, in conjunction with values of *newmut{t}*, to provide estimates of accumulation of specific classes of mutation consistent with those observed in clinical practice (75, 110, 126) They reflect a greater propensity for some mutations to arise than others. This probably relates to the ability of the virus to replicate without the mutations (e.g. probably very low in the presence of 3TC for virus without M184V) as well as the replicative capacity of virus with the mutations.

10.25 New resistance to NNRTI arising as a result of ART interruption

It is assumed that due to the long half life of NNRTIs nevirapine and efavirenz, stopping of a regimen containing one of these drugs is associated with a specific probability of an NNRTI resistance mutation arising (see, for example, Fox et al, 2008 (47). The respective probabilities for K103, Y181 and G190 are 1.8%, 0.06% and 0.6%.

10.26 Loss of acquired mutations from majority virus

It is assumed that mutations tend to be lost from majority virus with a certain probability from 3 months after stopping to take a drug that selects for that mutation. The probability of losing mutations per 3 months (from 3 months after stopping) is as follows (127-133).

Table S24. Probability of loss of acquired mutations from majority virus per 3 months after stopping drugs selecting for mutation.

M184V	0.8
L74V	0.6
Q151M	0.6
K65R	0.6
TAMS*	0.4
NNRTI mutations	0.05
Protease mutations	0.2
Dolutegravir mutations	0.2

* to lose all TAMS

Mutations are regained in majority virus if a drug selecting for the mutation is again started.

10.27 Determination of level of resistance to each drug

Table S25. shows the level of resistance to each drug according to presence of specific resistance mutations.

Table S25. Level of resistance to each drug according to presence of specific resistance mutations.

**Resistance mutation**	**Drug**	**Level of resistance (1=full resistance)**	**Condition**
M184	3TC or FTC	0.75	
1-2 TAMS	zidovudine	0.5	No 3TC or FTC in regimen
	zidovudine	0.25	3TC or FTC in regimen and ever had M184V
	zidovudine	0.5	3TC or FTC in regimen and never had M184V
2-3 TAMS	tenofovir	0.5	
3-4 TAMS	zidovudine	0.75	No 3TC or FTC in regimen
	zidovudine	0.5	3TC or FTC in regimen and ever had M184V
	zidovudine	0.75	3TC or FTC in regimen and never had M184V
4 or more TAMS	tenofovir	0.75	No 3TC or FTC in regimen, or 3TC in the regimen and never had M184V
	tenofovir	0.5	3TC or FTC in regimen and ever had M184V
5 or more TAMS	zidovudine	1.0	No 3TC or FTC in regimen
	zidovudine	0.75	3TC or FTC in regimen and ever had M184V
	zidovudine	0.75	3TC or FTC in regimen and never had M184V
Q151	3TC or FTC	0.25	
	zidovudine	0.75	
K65	3TC or FTC	0.25	
	tenofovir	0.75	
K103	efavirenz	1.0	
Y181	efavirenz	0.75	
G190	efavirenz	0.75	
I501	atazanavir	1.0	
N88	atazanavir	1.0	
I84	atazanavir	1.0	
1 - 3 of (V32, M46, I54, V82, L90)	atazanavir	0.5	
At least 4 of (V32, M46, I54, V82, L90)	atazanavir	1.0	
primary dolutegravir mutation only	dolutegravir	0.75	
secondary dolutegravir mutation only	dolutegravir	0.25	
primary and secondary dolutegravir mutation	dolutegravir	1.00	

These rules approximately follow the interpretation systems for conversion of mutations present on genotypic resistance test into a predicted level of drug activity (or, equivalently, of resistance; http://hivdb.stanford.edu, http://www.hivfrenchresistance.org/

10.28 Calculation of activity level of each drug

For drugs with a potency of 1 the activity level is 1-level of resistance. For ritonavir boosted PIs, which are asigned a potency of 2 it is given by 2 – (2 x level of resistance). Potency is assumed higher due to the ability to induce sustained viral suppression alone. Activity levels of each drug in the regimen are summed to give the total number of active drugs. For dolutegravir the potency is assumed to be 1.5 (the modal value of the distribution) so the activity is 1.5 – (1.5 x level of resistance). We also consider a range of values for the potency of dolutegravir, as described below.

10.29 Risk of clinical disease and death in HIV infected people

Occurrence of WHO 4 diseases

The rate of WHO 4 diseases according to CD4 count per 3 months is given below.

Table S26. Rate of WHO stage 4 disease according to CD4 count and viral load.

If cd4 ≥ 650	rate=0.002	if 500 ≤ cd4 < 650	rate=0.010
if 450 ≤ cd4 < 500	rate=0.013	if 400 ≤ cd4 < 450	rate=0.016
if 375 ≤ cd4 < 400	rate=0.020	if 350 ≤ cd4 < 375	rate=0.022
if 325 ≤ cd4 < 350	rate=0.025	if 300 ≤ cd4 < 325	rate=0.030
if 275 ≤ cd4 < 300	rate=0.037	if 250 ≤ cd4 < 275	rate=0.045
if 225 ≤ cd4 < 250	rate=0.055	if 200 ≤ cd4 < 225	rate=0.065
if 175 ≤ cd4 < 200	rate=0.080	if 150 ≤ cd4 < 175	rate=0.10
if 125 ≤ cd4 < 150	rate=0.13	if 100 ≤ cd4 < 125	rate=0.17
if 90 ≤ cd4 < 100	rate=0.20	if 80 ≤ cd4 < 90	rate=0.23
if 70 ≤ cd4 < 80	rate=0.28	if 60 ≤ cd4 < 70	rate=0.32
if 50 ≤ cd4 < 60	rate=0.40	if 40 ≤ cd4 < 50	rate=0.50
if 30 ≤ cd4 < 40	rate=0.80	if 20 ≤ cd4 < 30	rate=1.10
if 10 ≤ cd4 < 20	rate=1.80	if 0 ≤ cd4 < 10	rate=2.50

Independent effect of viral load

if v < 3	rate = rate x 0.2
if 3 <= v < 4	rate = rate x 0.3
if 4 <= v < 4.5	rate = rate x 0.6
if 4.5 <= v < 5	rate = rate x 0.9
if 5 <= v < 5.5	rate = rate x 1.2
if 5.5 <= v	rate = rate x 1.6

This is informed by Phillips et al (134)

Independent effect of age

rate = rate x (age / 38)$^{1.2}$

Independent effect of PJP prophylaxis

If patient on PJP prophylaxis then this rate is multiplied by 0.8. If CD4 count is measured and current value < 350 /mm3 then patient assumed to have 80% chance of starting PJP prophylaxis after 1996. If patient has current WHO stage 3 or 4 condition they are assumed to have an 80% chance of starting PJP prophylaxis. If the CD4 count is measured then PJP prophylaxis assumed to stop if current value > 350/mm3. If the patient has been continuously on ART for 2 years with no WHO 3 or 4 condition in previous 6 months then it is assumed that PJP prophylaxis is stopped.

Independent effect of being on ART

For patients on a single drug regimen this risk is multiplied by 0.9, for patients on a two drug regimen it is multiplied by 0.85 and for patients on a 3 drug regimen it is multiplied by 0.6, to reflect that being on ART has a positive effect on risk of AIDS and death independent of latest CD4 count and viral load.

Occurrence of WHO 3 diseases

As for WHO 4 except risk is fold_incr_who3 (= 5) higher.

Risk of HIV-related death

As for WHO 4 except risk *fold_decr_hivdeath* - fold lower (= 0.25).

CD4-, viral load- age-specific death rate raised *incr_death_rate_tb*-fold (= 10) if current TB and *incr_death_rate_adc*-fold (= 10) if current WHO 4 disease. We assume 15% of HIV-related deaths (ie not including deaths that arise due to background mortality rates) are classified as non-HIV-related.

11 Distributions for parameters

In Table S27 below, we describe all the parameters from which we sample for each model run to create the 3000 seting scenarios.

Table S27. Parameter distributions sampled for each model run together with the distribution for the 3000 setting-scenarios.^ &

**Parameter name**	**Description**	**Distribution sampled (value; % with value)**	**Motivation for distribution** (see also Table 1 of main paper which compares epidemic characteristics with observed data)
Population demographics			
inc_cat	Three future demographic structures with differing levels of population growth.	1: 33% 2: 33% 3: 33%	Different countries in sub-Saharan Africa have different population growth rates so we consider a range from around 1% to 3% per year (135)
Parameters relating to sexual behaviour			
sex_beh_trans_matrix_m and sex_beh_trans_matrix_m	Matrix determining rate of transition between four levels of sexual behaviour. There are 15 versions for each of men and women.	1/15 probability for each transition matrix for men, the same for women	Due to the fact that data on sexual behaviour are from self-report, which is known to be highly unreliable, there is uncertainty over longitudinal patterns of sexual behaviour and the degree of skewness in the distribution of number of new partners we consider a range of possible matrices (15 for each gender = 225 possible combinations).<br><br>Jointly with other parameters in this section these help to determine the extent to which risk behaviour is concentrated both between individuals and within individuals over time (140)
sex_age_mixing_matrix_m and sex_age_mixing_matrix_w	Matrix determining the age gender sexual mixing from male and female perspectives	6 different matrices for each gender persective, sampled at random	Uncertainty about mixing patterns by age and gender so we consider a range of these.
p_rred_p	Indicates the proportion of the population in whom the sexual risk behaviour is very low	0.3: 33% 0.5: 33% 0.7: 33%	In order to include a person-level effect on sexual behaviour this and the parameter below allow the population to be divided into three according to the lifelong tendency to have short term condomless sex partners.
p_hsb_p	Indicates the proportion of the population in whom the sexual risk behaviour has a tendency to be higher than average	0.05: 33% 0.08: 33% 0.15: 33%	As above
rred_a_p	Relative condomless sex levels by gender and age; four different patterns.	1: 15% 2: 15% 3: 35% 4: 35%	Uncertainty over levels of condomless sex by age so we consider a range of possibilities
eprate	Base rate (youngest age group) of starting to have a long term condomless sex partner.	Lognormal(0.1, 0.25)	Informed by outputs that give the proportion of people with a long term condomless sex partner by age.

Parameter name	Description	Distribution sampled (value; % with value)	Motivation for distribution (see also Table 1 of main paper which compares epidemic characteristics with observed data)
conc_ep	Parameter indicating the degree to which those with a primary condomless sex partner have a lower of higher probability of short term (non-primary) condomless sex partners than those without a primary condomless sex partner.	0.333: 33% 1: 33% 3: 33%	This is likely to vary across setting scenarios and we wished to consider across the range. Again, this distribution of values was found, in certain (randomly selected) combination with other sexual behaviour parameter values to re-produce epidemics within the observed prevalence range.
ych_risk_beh_newp	Degree of reduction in condomless sex with short term partners per year from 1995 – 2000	0.50: 5% 0.60: 45% 0.70: 30% 0.80: 15% 0.90: 5%	In order to explain the decrease in incidence and prevalence of HIV in southern Africa in the late 1990s it is necessary to assume there was a reduction in condomless sex, which is supported by data in Zimbabwe (10, 138, 139)
ych_risk_beh_ep	Degree of reduction in condomless sex per year with long term partners from 1995-2000	0.8: 25% 0.9: 25% 0.95: 25% 1.0: 25%	As above
ch_risk_diag_newp	Degree of reduction (fold change) in condomless sex with short term partners in a person diagnosed with HIV	0.7: 25% 0.8: 25% 0.9: 25% 1.0: 25%	Informed by (5)
ch_risk_diag	Degree of reduction in condomless sex with long term partner in a person diagnosed with HIV	0.7: 25% 0.8: 25% 0.9: 25% 1.00: 25%	As above
ych2_risk_beh_newp	Degree of change in condomless sex with short term partners per year from 2010 – 2015	0.90: 5% 0.95: 5% 1.0: 90%	It is uncertain whether there have been recent changes in condomless sex, hence a neutral distribution was used.
exp_setting_lower_p_vl1000<br><br>*external_exp_factor*<br>*rate_exp_set_lower_p_vl1000*	Whether there is exposure of individuals to settings with lower population viral suppression levels, due to migration (and return)<br>Measure of level of effect of the above exposure<br>Rate of exposure	In 20% of runs<br><br>Uniform(1,2)<br>Uniform(0, 0.01)	A subset of a population may have sexual exposure to others outside the population and the degree of this will be setting dependent so this is varied across model runs.
higher_newp_with_lower_adhav	In people with lower adherence to ART there is a tendency for lower number of condomless partners	In 20% of runs	There could be correlation between ART adherence and sexual risk behaviour, in either direction
base_rate_sw	Base rate per 3 months of a woman becoming a sex worker (also influenced by age and lifetime propensity)	0.0015: 20% 0.0020: 60% 0.0025: 20%	Informed by data on the proportion of women who are sex workers (136)
base_rate_stop_sexwork	Base rate per 3 months of a sex worker stopping sex work (also influenced by age)	0.010: 33% 0.015: 33% 0.030: 33%	Informed by data on the proportion of women who are sex workers and duration of sex work (136)
sw_trans_matrix	Transition matrices determining probabilities of transition between categories of number of condomless partners had in the 3 month period by sex workers. (See Table S9 above)	1: 20% 2: 20% 3: 20% 4: 20% 5: 20%	We consider a range of matrices to reflect uncertainty. Reports of condomless partner numbers are associated with inaccuracy of recall and potential bias in over-estimating consistency of condom use. (137)

Parameter name	Description	Distribution sampled (value; % with value)	Motivation for distribution<br>(see also Table 1 of main paper which compares epidemic characteristics with observed data)
sw_init_newp	Distribution of categories of number of condomless partners had in the 3 month period in first 3 month period of sex work	1: 67% (5% 0 newp) 2: 33% (20% 0 newp);	See above
p_rred_sw_newp	Effect of population level changes in sexual behaviour on the probability of starting and stopping sex work and/or moving to a lower category of number of condonless sex partners (see section 3 above)	0.01: 33% 0.03: 33% 0.10: 33%	Population level changes in sexual behaviour are believed to partially explain changes in HIV incidence in early phases of the HIV epidemic. (138, 139)
**Parameters relating to male circumcision**			
circ_inc_rate<br><br>*rel_incr_circ_post_2013*<br><br>*circ_inc_15_19*<br>*circ_red_20_30*<br>*circ_red_30_50*<br><br>*prob_birth_circ*	Determines the rate with which male circumcision increases over time<br><br>Relative increase in VMMC after 2013<br><br>Relative increases in age groups<br><br>Probability of circumcision at birth	0.0001: 10% 0001: 30% 0.003: 40% 0.01: 10% 0.10: 10%<br><br>0.8: 10% 1: 25% 3: 25% 7: 40%<br><br>1.5: 33% 2.0:33% 3.0: 33%<br>0.3: 33% 0.4:33% 0.5: 33%<br>0.15: 33% 0.25:33% 0.35: 33%<br><br>0.05: 30% 0.1: 40% 0.4: 20% 0.9: 10%	This varies by country in the region (8).
**Parameters relating to oral PrEP**			
rate_test_startprep	Additional rate of being tested for HIV (because of interest in PrEP) in people who have never been on PrEP but are eligible for it. Represents the probability he or she can access it easily enough to mean that they do indeed start.	0.25: 33% 0.5: 33% 0.75: 33%<br>The value changes to 0.9 in mid-2021 under the PrEP scale up policy option.	Distribution is chosen to reflect the range of settings and to encompass potential future higher levels of PrEP uptake and use. See results in main paper for distribution of proportion of people fullfilling criteria for risk_informed PrEP who are actually on PrEP.
rate_test_restartprep	Rate of being tested for HIV for people who are currently interrupting PrEP	0.50: 50% 0.80: 50%<br>The value changes to 0.9 in mid-2021 under the PrEP scale up policy option.	As above
rate_choose_stop_prep	Rate of discontinuing PrEP per 3 months (person's choice to stop despite risky condomless sex)	0.05: 33% 0.10: 33% 0.20: 33%<br>The value changes to 0.05 in mid-2021 under the PrEP scale up policy option.	As above
prob_prep_restart_choice	Probability of restarting PrEP after previous discontinuation when still having risky condomless sex	0.05: 33% 0.10: 33% 0.20: 33%<br>The value changes to 0.7 in mid-2021 under the PrEP scale up policy option.	As above

Parameter name	Description	Distribution sampled (value; % with value)	Motivation for distribution<br>(see also Table 1 of main paper which compares epidemic characteristics with observed data)
prepuptake_pop	Probability of willingness to take PrEP for non-sex workers. This determines the value of the individual level variable prep_willing_pop.	0.1: 20% 0.2: 60% 0.5:20%<br>Under PrEP scale-up in mid 2021 there is a 50% (75%, 95%) chance that a person who has prep_willing_pop = 0 becomes prep_willing_pop = 1. The values of 50%, 75% are 95% are sampled with equal probability for each setting scenario.	As above
prepuptake_sw	Extra probability of willingness to take PrEP for female sex workers compared with non-sex workers. This determines the value of the individual level variable prep_willing_sw.	0.1: 20% 0.5: 80%<br>Under PrEP scale-up in mid 2021 there is a 95% chance that a sex worker who has prep_willing_sw = 0 becomes prep_willing_sw = 1.	As above
prep_efficacy	PrEP efficacy (with 100% adherence)	0.90: 50% 0.95: 50%	(17)
sens_test_prep	Effective test sensitivity (also accounting for the possibility of the test not being performed)	0.5: 5% 0.7: 5% 0.9: 75% 0.95: 10% 1.0: 5%,	To reflect the possible range of effective test sensitivity, with the low levels intended to cover the possibility (as with self-tests) that the test is not done.
**Parameters relating to being hard to reach for services**			
*p_hard_reach_w_*<br><br>*hard_reach_higher_in_men_ p*	Proportion of women that have a propensity to be hard to reach with prevention and testing servcices<br><br>The extent to which this is higher in men (in men this also includes propensity to be medically circumcised)	Uniform(0.05, 0.15)<br><br>Uniform(0,0.1)	A small proportion of people have a long term propensity not to take up HIV services, for various possible reasons including stigma, physical barriers, etc. (23)
**Parameters relating to transmission**			
fold_change_w<br><br>*fold_change_yw*	The fold difference in female to male transmission rate compared with male to female, for a given viral load.<br><br>Rate is higher in younger women by *fold_change_yw*.	1: 5% 1.5: 25% 1.5: 60% 2: 70%<br><br>1: 33% 3: 33% 5: 33%	Informed by the higher incidence and prevalence in women in younger age groups and some direct evidence. (141, 142)

Parameter name	Description	Distribution sampled (value; % with value)	Motivation for distribution<br>(see also Table 1 of main paper which compares epidemic characteristics with observed data)
fold_change_sti	The fold difference in HIV acquisition risk for a person with a current STI.	2: 33% 3: 33% 5:33%	Multiple studies show a raised risk of acquisition but uncertainty over the effect size. (13)
fold_tr	A higher or lower risk of acquiring HIV for a given viral load in the partner	1: 33% 0.67: 33% 1.5: 33%	The convey uncertainty in the estimate of transmission rates
fold_tr_newp	Fold transmission rate per 3 months for short-term partners compared with long-term partners	0.3: 9% 0.5: 9% 0.7: 9% 0.8: 9% 0.9: 9% 1.0: 9% 1/0.8: 9% 1/0.6: 9% 1/0.4: 9%	Due to assumed lower number of sex acts in the 3 month period with short-term partners than long term
res_trans_factor_nn	Parameter affecting the probability that if NNRTI resistance mutation present in source partner that this is not present/detectable in virus new host	0.5: 20% 0.7: 20% 0.8: 20% 0.90: 20% 1.00: 20%	Informed by the values needed to lead to the range of transmitted NNRTI resistance observed (see Table 1 in paper)
res_trans_factor_ii	Parameter affecting the probability that if integrase inhibitor resistance mutation present in source partner that this is not present/detectable in virus new host	1: 80% 2: 20%	Little data available to inform this yet – we make the assumption in 80% of runs that transmission occurs.
super_infection	Whether we consider super-infection (which means a person with HIV can acquire HIV drug resistant HIV through a subsequent infection with a new viral strain).	Occurs in 50% of runs.	Super-infection can occur but its significance is uncertain but unlikely to be substantial. (19)
**Parameters relating to HIV testing**			
an_lin_incr_test	Parameter determining the rate of increase in HIV testing (any testing outside ANC)	0.0001: 10% 0.0005: 20% 0.003: 40% 0.01: 20% 0.02: 5% 0.04: 5%	Range and pattern required to re-produce the observed range in proportion of HIV positive people diagnosed (see Table 1 of main paper).
*date_test_rate_plateau_*	Year in which the rate of HIV testing plateaus.	2011: 10% 2013: 10% 2015: 20% 2017: 30% 2019: 30%	Some countries have increased testing rates markedly and these have plateaued at different levels in different settings (e.g 71)
rate_testanc_inc	Rate of increase in testing in ANC clinics	0.03: 33% 0.05: 33% 0.1: 33%	Distribution is intended to reflect variation across setting scenarios. (71)
*incr_test_rate_sympt_*	The rate of increase per 3 months in the probability of a person with a WHO stage 3 or 4 disease is tested for HIV.	1.05: 20% 1.10: 20% 1.15: 20% 1.20: 20% 1.25: 20%	Little direct data on this parameter and wide range taken to reflect uncertainty and variation across settings.
max_freq_testing	A parameter defining the maximum frequency with which a person (non sex-worker) without AIDS or WHO stage 3 disease can test for HIV	Annually: 80% 6-monthly: 20%	Policy on frequency of testing varies by setting.
test_targeting	Parameter conveying the degree to which HIV testing is targetted towards people having condomless sex since last test.	1.25: 80% 1.5: 20%	Data on condomless sex since last test not collected but likely to be a higher tendency to test if had sexual risks. We vary the degree of such "targeting". Partially informed by outputs on testing yield.

Parameter name	Description	Distribution sampled (value; % with value)	Motivation for distribution (see also Table 1 of main paper which compares epidemic characteristics with observed data)
sens_vct_test_type_3	Sensitivity of rapid HIV tests – important especially for when a person is starting PrEP, to inform the probability a person with recent HIV infection starts PrEP due to a negatiev HIV test.	95%: 20% 98%: 80%	(143)
**Parameters relating to pre-ART care and progression of HIV**			
fx	Multiplicative factor to alter the average rate of CD4 count decline in natural HIV progression (which thus alters the incubation period distribution).	0.7: 20% 0.85: 20% 1.0: 20% 1/0.85: 20% 1/0.7: 20%	Derived based on consideration of evidence from natural history studies (35-39)
gx	Multiplicative factor allowing expression of uncertainty in rates of viral load increase over time in people untreated	1.0: 33% 1.5: 33% 2.0: 33%	There is uncertainty over rates of viral load increase (35)
prob_loss_at_diag<br><br>*prob_lossdiag_adctb*<br>*prob_lossdiag_who3e*	Probability that a person is immediately lost after initial HIV diagnosis.<br><br>…if has an AIDS disease or TB at time of diagnosis<br>…if has an AIDS disease or TB at time of diagnosis	0.02: 20% 0.05: 20% 0.20: 20% 0.35: 20% 0.50: 10% 0.80: 10%<br><br>Beta(5,95)<br>Beta(15,85)	e.g. (144)
rate_lost	For people under care yet to start ART or previously have taken ART, the rate of being lost to care per 3 mths.	0.2: 33% 0.35: 33% 0.5: 33%	Uncertain and will vary by setting. Distribution chosen to reflect this. This is one of the parameters influencing the proportion of diagnosed people on ART.
rate_return	Probability of return to care for a person who has been diagnosed with HIV (and may have started ART) but is now lost and not on ART, without current WHO stage 3 or 4 disease, per 3 months.	0.01: 10% 0.10: 60% 0.30: 15% 0.6: 15%	As above
prob_return_adc	Probability of return to care for a person who has been diagnosed with HIV (and may have started ART) but is now lost and not on ART and has a WHO stage 4 condition. This is a probability that operates just for the 3-month period that the events occurs.	0.7: 20% 0.8: 30% 0.9: 50%	As above
rate_loss_persistence	Rate of loss from majority virus of transmitted resistance mutations (per 3 months)	0.00: 10% 0.005: 10% 0.01: 10% 0.015: 40% 0.02: 30%	(e.g. 145, 146)

Parameter name	Description	Distribution sampled (value; % with value)	Motivation for distribution<br>(see also Table 1 of main paper which compares epidemic characteristics with observed data)
**Parameters relating to people on ART**			
adh_pattern	Population adherence profile; described in terms of the proportion having a given average adherence and period-to-period variability in adherence. Note that adherence is additionally affected by age and gender.	1: 5% 2: 55% 3: 10% 4: 10% 5: 10% 6: 5% 7: 5%	Reflection of wide range of adherence profiles in different settings, informed by differences in proportions of people on ART with viral load suppression. This range leads to a range of levels of viral suppression and of resistance.
red_adh_tb_adc<br><br>*red_adh_tox_pop*<br><br>*add_eff_adh_nnrti*<br><br>*red_adh_multi_pill_pop_*<br><br>*altered_adh_sec_line_pop*	Reduction in adherence to ART associated with currently having an AIDS defining condition / TB<br><br>The extent to which drug toxicity influences adherence to ART negatively.<br><br>NNRTI drugs tend to have a longer half-life than PIs – this indicates the gain in effective adherence from NNRTIs due to this effect.<br><br>The extent to which taking multiple ARVs separately tends to lead to lower adherence than a single once daily pill.<br><br>The extent of any increase in adherence in people switched to second line ART – this is in addition to any effects of enhanced adherence counselling after a measured viral load > 1000 copies/mL.	logNormal(0.1, 0.5)<br><br>logNormal(red_adh_tox_pop_v, 0.5) where red_adh_tox_pop_v = 0.05 50% 0.10 50%<br>logNormal(0.10, 0.30)<br><br>logNormal(red_adh_multi_pill_pop_v, 0.5) where red_adh_multi_pill_pop_v = 0.05 33% 0.10 33% 0.15 33%<br><br>logNormal(0.05, 0.05)	(147)
pr_art_init	Probability of ART initiation per 3 months in a person in care who is eligible according to current criteria.	0.4: 25% 0.5: 25% 0.6: 25% 0.7: 25%	These parameters contribute to determine the proportion of HIV diagnosed people who are on ART. The distributions are chosen such that combinations of these parameters lead to observed proportions of HIV diagnosed people on ART (e.g. 72)
prob_lost_art	For a person who interrupts / stops ART the probability that they are simultaneously lost from care.	0.5: 20% 0.6: 20% 0.7: 20% 0.8: 20% 0.9: 20%	(e.g. 148)
rate_restart	Rate of restart of ART for people who previously have been on ART and have returned to care, per 3 months.	0.8: 25% 0.85: 25% 0.9: 25% 0.95: 25%	Assumed to be high, given the person has returned to care. Most people who are regularly seen in clinics who have previously started ART are on ART.

Parameter name	Description	Distribution sampled (value; % with value)	Motivation for distribution<br>(see also Table 1 of main paper which compares epidemic characteristics with observed data)
rate_int_choice	Rate of interruption / stopping of ART per 3 months. Also influenced by current drug toxicity and underlying tendency to adhere.	0.002: 33% 0.004: 33% 0.008: 33%	(148)
incr_rate_int_low_adh	Parameter indicating the extent to which people with a long-term average adherence in the lowest group have a multiplicatively increased risk of ART interruption.<br>Effect of current low adherence on risk of treatment interruption / discontinuation.	1: 50% 2: 25% 5: 25%	(149)
pr_switch_line	Probability of switch to second line per 3 months in a person who has fulfilled the failure criteria for first line failure.	0.20: 75% 0.50: 25%	In several settings, including Zimbabwe, the proportion of people who have started second line ART is consistent with a value for *pr_switch*_line of below 0.1 (e.g. Lesotho, Malawi)<br>(71, 45, 46)
clinic_not_aw_int_frac	If a person interrupts ART, the probability that this is not disclosed to the clinic and they are classified as being on ART	0.1: 20% 0.3: 20% 0.5: 20% 0.7: 20% 0.9: 20%	Uncertain and will vary by setting, hence a broad distribution.
fold_change_mut_risk	Fold difference in rate of accumulation of mutations (for all drugs) compared with base case.	1: 80% 2: 10% 0.5: 10%	To consider that the rate of resistance mutation acquisition is higher or lower than the rate assumed, reflecting some uncertainty. This relates to all resistance mutations.
*rate_res_ten_*	Parameter reflecting the rate of acquisition of tenofovir resistance. The value of 0.1 was derived based on European cohort data and the value of 0.3 reflects the potentially higher value for subtype C in southern Africa.	0.1: 50% 0.2: 50%	(150)
poorer_cd4_rise_on_fail_nn<br><br>*poorer_cd4_rise_on_fail_ii*	This indicates the extent of poorer CD4 rise per 3 months on failing NNRTI based regimens (compared with PI)<br>This indicates whether the poorer CD4 rise also applied to failing INSTI based regimens	Normal(-6,3)<br><br>no: 50% yes: 50%	(118)
adh_effect_of_meas_alert	The effect of having a viral load measured > 1000 copies/mL on adherence, due to the enhanced adherence intervention.	0.35:15% 0.70: 70% 0.90: 15%	Uncertainty over the effect size.
prob_vl_meas_done	Probability of a viral loead measure being done. This probability operates for each time a viral load is due to be tested.	0.0: 5% 0.1: 30% 0.7: 50% 1.00: 15%	Variation in viral load implementation in different settings. Note that in half of settings with value 0 there is CD4 count monitoring of people on ART in place.
cd4_monitoring	If viral load monitoring is not being implemented (*prob_vl_meas_done = 0*), if CD4 count monitoring being done ?	if prob_vl_meas_done = 0 then<br>no: 50% yes 50%	This will vary by setting.

**Parameter name**	**Description**	**Distribution sampled (value; % with value)**	**Motivation for distribution**<br>(see also Table 1 of main paper which compares epidemic characteristics with observed data)
switch_for_tox	Whether the ART program manages to implement drug substitutions in response to specific toxicities experienced by patients.	No: 80% Yes: 20%	This will vary by program but generally not widespread.
zero_3tc_activity_m184	activity of 3TC in presence of M184V mutation	No: 80% Yes: 20%	To consider alternative assumptions; distribution broadly reflects the uncertainty.
zero_ten_activity_k65	activity of 3TC in presence of K65R mutation	No: 80% Yes: 20%	To consider alternative assumptions; distribution broadly reflects the uncertainty.
higher_rate_res_dol	Whether there is a higher rate of resistance to dolutegravir than the base assumption (i.e. 4 times lower than efavirenz compared with 13 times lower in base case).	No: 80% Yes: 20%	To consider alternative assumptions; distribution broadly reflects the uncertainty.
*prop_bmi_ge23_*	Proportion of people initiating dolutegravir who have BMI $\geq$ 23	0.5: 50% 0.75: 50%	Uncertainty over the proportion of the population starting dolutegravir who have BMI > 23 and hence a possible negative effect of weight gain on dolutegravir.<br>(151, 152)
*incr_mort_risk_dol_weightg_*	Rate ratio for mortality in people on dolutegravir who had BMI $\geq$ 23 at start, due to dolutegravir induced weight gain.	1: 1% 1.1: 16% 2: 17% 2.1: 17% 2.2: 17% 3.0: 17% 4.0: 15%	Wide distribution within plausible bounds reflecting uncertainty (153-156)
nnrti_res_no_effect	Effect of NNRTI resistance mutations on activity of efavirenz (base case: K103N 0 activity, G190A 0.25 activity, Y181C 0.25 activity)	0.25: 25% 0.5: 5% 0: 75%	Some uncertainty over this. With this distribution the average odds ratio for VL > 1000 at 1 year from start of ART associated with pre-treatment NNRTI drug resistance = 3.3 (compared with 3.9 in a recent meta-analysis (157)
tox_weightg_dol	Whether weight gain is treated as a toxicity that has an associated increased risk of ART interruption	no: 50% yes: 50%	Weight gain does not seem to be mentioned as troublesome to people on dolutegravir, but this could change with time.
*rel_dol_tox_*	Relative rate of neurologic toxicity (sleep disturbance for dolutegravir and dizziness and vivid dreams for efavirenz)	0.5 fold that of efavirenz 80 / 81<br>Equal to efavirenz 20 / 19	While evidence suggests neurologic toxicity is higher with efavirenz, there is uncertainty over size of effect of insomnia with dolutegravir so we consider the possibility that the overall neurologic toxicity of the two drugs could be equal.
double_rate_gas_tox_taz	Parameter related to the rate of gastrointestinal toxity relating to atazanavir. Whether base rate is doubled or not.	Yes: 50% No: 50%	Uncertainty over gastrointestinal toxity relating to atazanavir – consider possibility that this has been underestimated.
lower_future_art_cov	Whether future coverage of ART is below that predicted by continuation in current trend in rates.	No:93% Yes: 7%	To reflect uncertainty.
rr_int_tox	Increased rate of ART interruption according to presence of a drug toxicity.	2-fold: 33% 10-fold: 33% 30-fold: 33%	Consider possibiity that ART interruption is substantially more highly related to drug toxicity than base case

Parameter name	Description	Distribution sampled (value; % with value)	Motivation for distribution<br>(see also Table 1 of main paper which compares epidemic characteristics with observed data)
greater_disability_tox	Parameter to allow consideration of a greater disability weight associated with drug toxicity (0.25) compared with the base assumption (of 0.05)	No: 50% Yes: 50%	To reflect the uncertainty and perceived relatively low likelihood that value is as high as 0.25.
*greater_tox_zdv_*	Whether the toxicity associated with zidovudine is greater than the base assumption.	No: 33% Yes, 2-fold: 33% Yes, 4-fold: 33%	(158)
zdv_potency_p75	Whether potency of zdv is 0.75 of an active drug rather than 1.	No: 50% Yes: 50%	To reflect uncertainty
sw_art_disadv	Whether sex workers have lower engagement in HIV care, specifically in relation to treatment interruptions, adherence and being lost at diagnosis	Yes: 50% No: 50%	Likely to vary by setting
sw_higher_int	If SW have lower engagement in HIV care, degree to which treatment interruptions may be higher	Probability of interrupting treatment is doubled	
prob_sw_lower_adh	If SW have lower engagement in HIV care, degree to which adherence may be lower	30% chance of lower adherence if originally in the highest adherence category	
sw_higher_prob_loss_at_diag	If SW have lower engagement in HIV care, degree to which adherence may be lower	50% increased risk of being lost at diagnosis	
higher_newp_less_engagement	Whether there is a tendency for people with more short term partners to be less likely to be engaged with ART care	No: 80% yes: 20%	Likely to vary by setting
**Parameter relating to pregnancy**			
prob_pregnancy_base	Parameter determining base rate of pregnancy for women having condomless sex (to which there is an effect of age)	Uniform (0.03, 0.11)<br>if inc_cat = 1 then prob_pregnancy_base increased 1.75-fold<br>if inc_cat = 3 then prob_pregnancy_base decreased 1.75 -fold	Variability between settings in fertility rate.
rate_birth_with_infected_child	Parameter determining the risk of mother to child transmission (MTCT), for a given level of mother viral load.	0.3: 5% 0.4: 25% 0.5: 60% 0.6: 10%	To produce plausible variation in the MTCT rate.
*oth_dol_adv_birth_e_risk_*	Risk of dolutegravir-induced adverse birth event, due to dolutegravir-induced weight gain	0.0005: 20% 0.0015: 40% 0.002: 20% 0.003: 20%	Wide distribution within plausible bounds reflecting uncertainty. (159)
**Parameters relating to sex worker programmes**			
sw_program	*sw_program*	*sw_program*	*sw_program*

Parameter name	Description	Distribution sampled (value; % with value)	Motivation for distribution<br>(see also Table 1 of main paper which compares epidemic characteristics with observed data)
effect_sw_prog_newp	*effect_sw_prog_newp*	*effect_sw_prog_newp*	*effect_sw_prog_newp*
effect_sw_prog_6mtest	Parameter determining strength of sex worker program in relation to 6-monthly testing for HIV	0.25: 33% 0.50: 33% 0.75: 33%	
effect_sw_prog_int	Parameter determining strength of sex worker program in reducing any disadvantage that SW may have in relation to number of treatment interruptions	0.4: 33% 0.6: 33% 0.8: 33%	
effect_sw_prog_adh	Parameter determining strength of sex worker program in reducing any disadvantage that SW may have in relation to low adherence	1.0: 33% 1.5: 33% 10: 33%	
effect_sw_prog_lossdiag	Parameter determining strength of sex worker program in reducing any disadvantage that SW may have in relation to being lost at diagnosis	0.4: 33% 0.6: 33% 0.8: 33%	
effect_sw_prog_prep	Parameter determining strength of sex worker program in increasing number of sex workers on PrEP	0.80: 50% 0.95: 50%	
effect_sw_prog_pers_sti	Parameter determining strength of sex worker program in reducing number of sex workers with STIs lasting greater than one 3-month period	0.50: 50% 0.70: 50%	
**Parameter for COVID-19 death risk**			
cov_death_risk_mult	There is a calendar time limited risk of death from COVID-19 which is age dependent. The base risk per 3 months during the epidemic is as follows<br><br>if 15 <= age < 20 then cov_deathrix = 0.0001<br>if 20 <= age < 30 then cov_deathrix = 0.0003<br>if 30 <= age < 40 then cov_deathrix = 0.0008<br>if 40 <= age < 50 then cov_deathrix = 0.0016<br>if 50 <= age < 60 then cov_deathrix = 0.006<br>if 60 <= age < 70 then cov_deathrix = 0.019<br>if 70 <= age < 80 then cov_deathrix = 0.043<br>if 80 <= age then cov_deathrix = 0.078<br><br>This parameter allows us to consider uncertainty in the form of a relative risk to allow consideration of higher risk.	1: 0.4 2: 40% 3: 20%	To reflect uncertainty

^ model program (in which these variable names are used) available on figshare (see main paper)

^^ model run with these parameter input values – only runs with HIV incidence age 15-49 in women 0.1 – 2.25 and in men 0.1 to 1.75 are selected. (18% of model runs / setting scenarios rejected for this reason)

12 Disability weights and costs

Table S28. Disability weights

Values are 1 in each three-month period except for the following:

Condition in current 3-month period	Disability weight for current 3-month period	Source
Any drug toxicity in current 3-month period<br><br>Any WHO stage 3 condition (except TB) in current 3-month period<br><br>TB in current 3-month period<br><br>Any WHO stage 4 condition in current 3-month period	0.05 (0.25 if greater_disability_tox =1)<br><br>0.22<br><br>0.40<br><br>0.54	(162)

Table S29. Unit Costs

Item	Unit Cost	Source / explanation
Drug costs per year:<br><br>TLE<br><br>TLD<br><br>ZL-PI (PI atazanavir)<br><br>ZLD	 <br>$78 ($65 without supply chain costs<br><br>$78 ($65 without supply chain costs)<br><br>$318 ($265 without supply chain costs)<br><br>$126 ($105 without supply chain costs)	(163
Cost of treatment of a WHO stage 4 condition over 3 months (cost is incurred for 3 months)<br><br>Cost of treatment of a WHO stage 3 condition over 3 months (cost is incurred for 3 months)<br><br>Cost of treatment of TB per 3 months (cost is incurred for 6 months)<br><br>Cotrimoxazole annual cost	$200<br><br>$20<br><br>$50<br><br>$5	Specific data not available on average unit costs of treating WHO stage 3 and 4 conditions and per clinic visit costs - costs used are informed by evidence synthesis from studies that cost according to current CD4 count of those in pre-ART care, cost of ART initiation, which also include costs of CD4 tests (164)
CD4 count measurement	$10	(165, 166)
Viral load measurement:	$22	Human resource costs $3, sample collection consumables $2, relaying of results $2 (this costing information was provided by Médecins Sans Frontierès (MSF) (including equipment and other costs such as consumables, maintenance and shipping) $15. Updates are consistent with this cost (167)
Non-ART programme costs per year, $40 per year if on tiered care due to viral load < 1000	$80	Bill and Melinda Gates Foundation tiered care meeting report (the per client cost of running the Khayelitsha adherence clubs was $58 per client per year compared to standard clinic care of $108 per client per year. At the Infectious Disease Institute in Kampala, the annual costs per client for physician, nurse, and pharmacy only visits were $60, $45, and $19, respectively) (168-170)

Item	Unit Cost	Source / explanation
Cost of the targeted adherence counselling intervention triggered by a viral load > 1000 copies/mL	$10	Assumption
HIV test (including personnel costs)	$3.70	Personal communication. CHAI.
Annual cost of treatment for a child born with HIV	$160	This cost was estimated based on a drug cost of $75 per year, a one-off cost of early infant diagnosis of $22, cost of viral load testing of $22 per year, costs of clinic visits of $40 or $80 per year (depending on whether viral load is suppressed), assuming 50% of children will achieve viral suppression. This is likely to be a lower limit cost per year.
VMMC	$106	
PrEP	$116 per year ($29 per 3 months)	$29 per 3 months, consisting of a drug cost of $11 (including supply chain costs, based on the South Africa tender price for PrEP drugs (16)), $4 for an HIV test per 3 months, and $14 per 3 months for additional costs necessary to facilitate education and access (including any costs of HBV or creatinine testing that might be done at first start).

References

1. Phillips, A.N., et al., Effect on transmission of HIV-1 resistance of timing of implementation of viral load monitoring to determine switches from first to second-line antiretroviral regimens in resource-limited settings. AIDS, 2011. 25(6).
2. Cambiano, V., et al., Transmission of Drug Resistant HIV and Its Potential Impact on Mortality and Treatment Outcomes in Resource-Limited Settings. The Journal of Infectious Diseases, 2013. 207(suppl_2): p. S57-S62.
3. Cambiano, V., et al., Predicted levels of HIV drug resistance: potential impact of expanding diagnosis, retention, and eligibility criteria for antiretroviral therapy initiation. AIDS, 2014. 28: p. S15-S23.
4. CIA. The World Factbook. 2021 [cited 2021 21st January]; Available from: https://www.cia.gov/the-world-factbook/.
5. Fonner, V.A., et al., Voluntary counseling and testing (VCT) for changing HIV-related risk behavior in developing countries. Cochrane Database Syst Rev, 2012. 9(9): p. Cd001224.
6. Desmond N, Nagelkerke N, Lora W, Chipeta E, Sambo M, Kumwenda M, et al. Measuring sexual behaviour in Malawi: a triangulation of three data collection instruments. BMC Public Health (2018) 18:807
7. Glynn JR, Kayuni N, Banda E, Parrott F, Floyd S, et al. (2011) Assessing the Validity of Sexual Behaviour Reports in a Whole Population Survey in Rural Malawi. PLoS ONE 6(7): e22840. doi:10.1371/journal.pone.0022840
8. Yeatman S, Trinitapoli J. Best-Friend Reports: A Tool for Measuring the Prevalence of Sensitive Behaviors. Am J Public Health. 2011;101:1666–1667. doi:10.2105/AJPH.2011.300194
9. Gregson, S., et al., Methods to Reduce Social Desirability Bias in Sex Surveys in Low-Development Settings: Experience in Zimbabwe. Sexually Transmitted Diseases, 2002. 29(10): p. 568-575.
10. Johnson, L. and R. Dorrington, The demographic and epidemiological impact of HIV/AIDS treatment and prevention programmes: an evaluation based on the ASSA2000 model. Paper presented at the Demographic Association of Southern Africa Conference, Cape Town, 26-27 September 2002, 2002.
11. Hollingsworth, T.D., Roy M. Anderson, and C. Fraser, HIV-1 Transmission, by Stage of Infection. The Journal of Infectious Diseases, 2008. 198(5): p. 687-693.
12. Bellan, S.E., et al., Reassessment of HIV-1 Acute Phase Infectivity: Accounting for Heterogeneity and Study Design with Simulated Cohorts. PLOS Medicine, 2015. 12(3): p. e1001801.
13. Cohen, M.S., Sexually transmitted diseases enhance HIV transmission: no longer a hypothesis. The Lancet, 1998. 351: p. S5-S7.
14. Bailey RC, et al. Male circumcision for HIV prevention in young men in Kisumu, Kenya: a randomised controlled trial. Lancet. 2007;369:643–56.
15. Auvert B, et al. Randomized, controlled intervention trial of male circumcision for reduction of HIV infection risk: the ANRS 1265 Trial. PLoS Med. 2005;2:e298.
16. Gray R, et al. The effectiveness of male circumcision for HIV prevention and effects on risk behaviors in a posttrial follow-up study. AIDS Lond Engl. 2012;26:609–15.
17. Heffron R, Ngure K, Odoyo J et al. Pre-exposure prophylaxis for HIV-negative persons with partners living with HIV: uptake, use, and effectiveness in an open-label demonstration project in East Africa [version 2; peer review: 2 approved] Gates Open Research 2018, 1:3 https://doi.org/10.12688/gatesopenres.12752.2
18. Castro, H. et al. Persistence of HIV-1 transmitted drug resistance mutations. Journal of Infectious Diseases 208, 1459-1463 (2013).

19. Smith, D.M., et al., HIV drug resistance acquired through superinfection. AIDS, 2005. 19(12): p. 1251-1256.
20. Corvasce et al. Evidence of differential selection of HIV-1 variants carrying drug-resistant mutations in seroconverters. Antiviral Therapy 2006; 11:329 -334.
21. Turner et al. Diminished Representation of HIV-1 Variants Containing Select Drug Resistance–Conferring mutations in Primary HIV-1 Infection. JAIDS 2004; 37: 1627-1631)
22. Phillips AN, Cambiano V, Nakagawa F, Brown AE, Lampe F, et al. (2013) Increased HIV Incidence in Men Who Have Sex with Men Despite High Levels of ART-Induced Viral Suppression: Analysis of an Extensively Documented Epidemic. PLoS ONE 8(2): e55312. doi:10.1371/journal.pone.0055312
23. Grimsrud A, Wilkinson L, Eshun-Wilson I, Holmes C, Sikazwe6 I, Katz IT. Understanding Engagement in HIV Programmes: How Health Services Can Adapt to Ensure No One Is Left Behind. Current HIV/AIDS Reports (2020) 17:458–466
24. Wanyenze, R. K. et al. Linkage to HIV care and survival following inpatient HIV counseling and testing. AIDS and Behavior 15, 751-760 (2011).
25. Bassett, I. V. et al. Routine voluntary HIV testing in Durban, South Africa: the experience from an outpatient department. Journal of acquired immune deficiency syndromes (1999) 46, 181 (2007)
26. Hensen, B. et al. Universal voluntary HIV testing in antenatal care settings: a review of the contribution of provider-initiated testing & counselling. Tropical Medicine & International Health 17, 59-70 (2012).
27. Sabapathy, K., Van den Bergh, R., Fidler, S., Hayes, R. & Ford, N. Uptake of home-based voluntary HIV testing in sub-Saharan Africa: a systematic review and meta-analysis. PLOS Medicine 9(12): e1001351 (2012).
28. Gibas KM, van den Berg P, Powell VE, Krakower DS. Drug Resistance during HIV Preexposure Prophylaxis. Drugs. 2019 April ; 79(6): 609–619. doi:10.1007/s40265-019-01108-x)
29. Lehman DA, Baeten JM, McCoy CO, Weis JF, Peterson D, Mbara G et al. Risk of Drug Resistance Among Persons Acquiring HIV Within a Randomized Clinical Trial of Single- or Dual-Agent Preexposure Prophylaxis. J Infect Dis 2015; 211, 8, 15: 1211–1218. https://doi.org/10.1093/infdis/jiu677
30. Parikh UM, Mellors JW. Should we fear resistance from tenofovir/emtricitabine preexposure prophylaxis ? Curr Opin HIV/AIDS 2016; 11: 49-55. DOI: 10.1097/COH.0000000000000209
31. Phillips, A. N. et al. Outcomes from monitoring of patients on antiretroviral therapy in resource-limited settings with viral load, CD4 cell count, or clinical observation alone: a computer simulation model. The Lancet 371, 1443-1451 (2008).
32. Phillips, A. N. et al. Effect on transmission of HIV-1 resistance of timing of implementation of viral load monitoring to determine switches from first to second-line antiretroviral regimens in resource-limited settings. AIDS 25, 843-850 (2011).
33. Nakagawa, F. et al. Projected life expectancy of people with HIV according to timing of diagnosis. AIDS 26, 335-343 (2012).
34. Nakagawa, F. et al. Projected lifetime healthcare costs associated with HIV infection. PLOS ONE 10(4): e0125018 (2015).
35. Pantazis N, Touloumi G. Bivariate modelling of longitudinal measurements of two human immunodeficiency type 1 disease progression markers in the presence of informative drop-outs. JRSS C 2005; 54: 405-423.
36. Sabin CA, Devereux H, Phillips AN, et al. Course of viral load throughout HIV-1 infection. JAIDS 2000; 23:172-177.

37. Hubert J-B, Burgard M, Dussaix E, et al. Natural history of serum HIV-1 RNA levels in 330 patients with known date of infection. AIDS 2000; 14:123-131.
38. O'Brien TR, Rosenberg PS, Yellin F, et al. Longitudinal HIV-1 RNA levels in a cohort of homosexual men. JAIDS 1998; 18:155-161.
39. Henrard DR, Phillips JF, Muenz LR et al. Natural history of HIV-1 cell-free viraemia. JAMA 1995; 274: 554-558.
40. Lyles, R. H. et al. Natural history of human immunodeficiency virus type 1 viremia after seroconversion and proximal to AIDS in a large cohort of homosexual men. Journal of Infectious Diseases 181, 872-880 (2000).
41. Touloumi, G. et al. Differences in HIV RNA levels before the initiation of antiretroviral therapy among 1864 individuals with known HIV-1 seroconversion dates. AIDS 18, 1697-1705 (2004).
42. Mellors, J. W. et al. Plasma viral load and CD4+ lymphocytes as prognostic markers of HIV-1 infection. Annals of internal medicine 126, 946-954 (1997).
43. Koot, M. et al. Prognostic value of HIV-1 syncytium-inducing phenotype for rate of CD4+ cell depletion and progression to AIDS. Annals of internal medicine 118, 681-688 (1993).
44. Darby SC, Ewart DW, Giangrande PL, Spooner RJ, Rizza CR. Importance of age at infection with HIV-1 for survival and development of AIDS in UK haemophilia population. UK Haemophilia Centre Directors' Organisation. Lancet 1996 Jun 8;347(9015):1573-9. doi: 10.1016/s0140-6736(96)91073-9.
45. Fox, M.P., et al., Rates and Predictors of Failure of First-line Antiretroviral Therapy and Switch to Second-line ART in South Africa. JAIDS Journal of Acquired Immune Deficiency Syndromes, 2012. 60(4): p. 428-437.
46. Johnston, V., et al., Outcomes following virological failure and predictors of switching to second-line antiretroviral therapy in a South African treatment program. Journal of acquired immune deficiency syndromes (1999), 2012. 61(3): p. 370-380.
47. Fox Z, Phillips AN, Cohen C, et al. Viral resuppression and detection of drug resistance following interruption of a suppressive non-nucleoside reverse transcriptase inhibitor-based regimen. AIDS 2008; 22:2279-2289.
48. Bangsberg DR, Moss AR, Deeks SG et al. Paradoxes of adherence and drug resistance to HIV antiretroviral therapy. J Antimicrob Chem 2004; 53 (5): 696-699.
49. Bangsberg, D.R., Acosta, E.P., Gupta, R., Guzman, D., Riley, E.D., Harrigan, P.R., Parkin, N., & Deeks, S.G. 2006. Adherence-resistance relationships for protease and non-nucleoside reverse transcriptase inhibitors explained by virological fitness. AIDS, 20, (2) 223-231 available from: PM:16511415
50. Bangsberg, D.R. 2006. Less than 95% adherence to nonnucleoside reverse-transcriptase inhibitor therapy can lead to viral suppression. Clin.Infect.Dis., 43, (7) 939-941 available from: PM:16941380
51. Hamers, R.L., Wallis, C.L., Kityo, C., Siwale, M., Mandaliya, K., Conradie, F., Botes, M.E., Wellington, M., Osibogun, A., Sigaloff, K.C., Nankya, I., Schuurman, R., Wit, F.W., Stevens, W.S., van, V.M., & de Wit, T.F. 2011. HIV-1 drug resistance in antiretroviral-naive individuals in sub-Saharan Africa after rollout of antiretroviral therapy: a multicentre observational study. Lancet Infect.Dis., 11, (10) 750-759 available from: PM:21802367
52. Hassan, A.S., Nabwera, H.M., Mwaringa, S.M., Obonyo, C.A., Sanders, E.J., Rinke de Wit, T.F., Cane, P.A., & Berkley, J.A. 2014. HIV-1 virologic failure and acquired drug resistance among first-line antiretroviral experienced adults at a rural HIV clinic in coastal Kenya: a cross-sectional study. AIDS Res.Ther., 11, (1) 9 available from: PM:24456757
53. Hoffmann CJ, Charalambous S, Sim J, et al. Viremia, Resuppression, and Time to Resistance in Human Immunodeficiency Virus (HIV) Subtype C during First-Line Antiretroviral Therapy in South Africa. Clin Infect Dis 2009; 49:1928–35.

54. Hoffmann, C.J., Charalambous, S., Grant, A.D., Morris, L., Churchyard, G.J., & Chaisson, R.E. 2014. Durable HIV RNA resuppression after virologic failure while remaining on a first-line regimen: a cohort study. Trop.Med.Int.Health, 19, (2) 236-239 available from: PM:24588012
55. Kobin, A.B. & Sheth, N.U. 2011. Levels of adherence required for virologic suppression among newer antiretroviral medications. Ann.Pharmacother., 45, (3) 372-379 available from: PM:21386024
56. Li, J.Z., Gallien, S., Ribaudo, H., Heisey, A., Bangsberg, D.R., & Kuritzkes, D.R. 2014. Incomplete adherence to antiretroviral therapy is associated with higher levels of residual HIV-1 viremia. AIDS, 28, (2) 181-186 available from: PM:24361679
57. Mackie, N.E., Phillips, A.N., Kaye, S., Booth, C., & Geretti, A.M. 2010. Antiretroviral drug resistance in HIV-1-infected patients with low-level viremia. J.Infect.Dis., 201, (9) 1303-1307 available from: PM:20350161
58. Rosenblum, M., Deeks, S.G., van der Laan, M., & Bangsberg, D.R. 2009. The risk of virologic failure decreases with duration of HIV suppression, at greater than 50% adherence to antiretroviral therapy. PLoS.One., 4, (9) e7196 available from: PM:19787058
59. Tran, D.A., Wilson, D.P., Shakeshaft, A., Ngo, A.D., Doran, C., & Zhang, L. 2014. Determinants of virological failure after 1 year's antiretroviral therapy in Vietnamese people with HIV: findings from a retrospective cohort of 13 outpatient clinics in six provinces. Sex Transm.Infect. available from: PM:24619575
60. Usitalo, A., Leister, E., Tassiopoulos, K., Allison, S., Malee, K., Paul, M.E., Smith, R., Van Dyke, R.B., Seage, G.R., III, & Mellins, C.A. 2014. Relationship between viral load and self-report measures of medication adherence among youth with perinatal HIV infection. AIDS Care, 26, (1) 107-115 available from: PM:23800360
61. von Wyl., V, Klimkait, T., Yerly, S., Nicca, D., Furrer, H., Cavassini, M., Calmy, A., Bernasconi, E., Boni, J., Aubert, V., Gunthard, H.F., Bucher, H.C., & Glass, T.R. 2013. Adherence as a predictor of the development of class-specific resistance mutations: the Swiss HIV Cohort Study. PLoS.One., 8, (10) e77691 available from: PM:24147057
62. Johannessen, A., Naman, E., Kivuyo, S.L., Kasubi, M.J., Holberg-Petersen, M., Matee, M.I., Gundersen, S.G., & Bruun, J.N. 2009. Virological efficacy and emergence of drug resistance in adults on antiretroviral treatment in rural Tanzania. BMC.Infect.Dis., 9, 108 available from: PM:19583845
63. Muyingo, S.K., Walker, A.S., Reid, A., Munderi, P., Gibb, D.M., Ssali, F., Levin, J., Katabira, E., Gilks, C., & Todd, J. 2008. Patterns of individual and population-level adherence to antiretroviral therapy and risk factors for poor adherence in the first year of the DART trial in Uganda and Zimbabwe. J.Acquir.Immune.Defic.Syndr., 48, (4) 468-475 available from: PM:18614918
64. Walmsley SL, Antela A, Clumeck N, Duiculescu D, Eberhard A, Gutierrez F, et al. Dolutegravir plus abacavir-lamivudine for the treatment of HIV-1 infection. N Engl J Med. 2013; 369(19):1807–18. doi: 10.1056/NEJMoa1215541 PMID: 24195548
65. Walmsley S, Baumgarten A, Berenguer J, Felizarta F, Florence E, Khuong-Josses MA, et al. Brief Report: Dolutegravir Plus Abacavir/lamivudine for the Treatment of HIV-1 Infection in Antiretroviral Therapy-Naive Patients: Week 96 and Week 144 Results From the SINGLE Randomized Clinical Trial. J Acquir Immune Defic Syndr. 2015;70(5):515–9. doi: 10.1097/QAI.0000000000000790. pmid:26262777
66. O'Connor JL, Gardner EM, Esser S, Mannheimer SB, Lifson AR, Telzak E, et al. A simple self-reported adherence tool as a predictor of viral rebound in people with viral suppression on antiretroviral therapy. HIV Medicine (2016), 17, 124—132.
67. Filimão DBC, Moon TD, Senise JF, Diaz RS, Sidat M, Castelo A (2019) Individual factors associated with time to non-adherence to ART pick-up within HIV care and treatment services in three health facilities of Zambe´zia Province, Mozambique. PLoS ONE 14(3): e0213804. https://doi.org/10.1371/journal.pone.0213804

68. Haberer JE, Bosco M, Bwana M, Orrell C, Asiimwe S, Amanyire G, Musinguzi N et al. ART adherence and viral suppression are high among most non-pregnant individuals with early-stage, asymptomatic HIV infection: an observational study from Uganda and South Africa JIAS 2019, 22:e25232.
69. Liegeois F, Eymard-Duvernay S, Boyer S, Maradan G, Kouanfack C, Domyeum J, et al. Heterogeneity of virological suppression in the national antiretroviral programme of Cameroon (ANRS 12288 EVOLCAM) HIV Medicine 2019; 20, 38—46
70. Jiamsakul A, Kariminia A, Althoff KN, Cesar C, Cortes CP, Davies M-A, et al. HIV Viral Load Suppression in Adults and Children Receiving Antiretroviral Therapy—Results From the IeDEA Collaboration. J Acquir Immune Defic Syndr 2017;76:319–329
71. Malawi Ministry of Health. Quarterly reports www.health.gov.mw
72. Population Health Impact Surveys. https://phia.icap.columbia.edu/
73. Chi, B.H., Cantrell, R.A., Zulu, I., Mulenga, L.B., Levy, J.W., Tambatamba, B.C., Reid, S., Mwango, A., Mwinga, A., Bulterys, M., Saag, M.S., & Stringer, J.S. 2009. Adherence to first-line antiretroviral therapy affects non-virologic outcomes among patients on treatment for more than 12 months in Lusaka, Zambia. Int.J.Epidemiol., 38, (3) 746-756 available from: PM:19223334
74. WHO HIV Drug Resistance Surveillance Report 2012 http://apps.who.int/iris/bitstream/handle/10665/75183/9789241503938_eng.pdf;jsessionid=B20E426C5A757C5F3DC01FA62A9F4F06?sequence=1
75. Cozzi-Lepri, A., UK HIV Drug Resistance, & UK CHIC 2010. Long-term probability of detecting drug-resistant HIV in treatment-naive patients initiating combination antiretroviral therapy. Clin.Infect.Dis., 50, (9) 1275-1285 available from: PM:20353366
76. Cheeseman, S.H., Hattox, S.E., McLaughlin, M.M., Koup, R.A., Andrews, C., Bova, C.A., Pav, J.W., Roy, T., Sullivan, J.L., & Keirns, J.J. 1993. Pharmacokinetics of nevirapine: initial single-rising-dose study in humans. Antimicrob.Agents Chemother., 37, (2) 178-182 available from: PM:8452345
77. Gardner, E.M., Burman, W.J., Steiner, J.F., Anderson, P.L., & Bangsberg, D.R. 2009. Antiretroviral medication adherence and the development of class-specific antiretroviral resistance. AIDS, 23, (9) 1035-1046 available from: PM:19381075
78. Gross, R., Bilker, W.B., Wang, H., & Chapman, J. 2008. How long is the window of opportunity between adherence failure and virologic failure on efavirenz-based HAART? HIV.Clin.Trials, 9, (3) 202-206 available from: PM:18547907
79. Meresse, M., March, L., Kouanfack, C., Bonono, R.C., Boyer, S., Laborde-Balen, G., Aghokeng, A., Suzan-Monti, M., Delaporte, E., Spire, B., Carrieri, M.P., & Laurent, C. 2014. Patterns of adherence to antiretroviral therapy and HIV drug resistance over time in the Stratall ANRS 12110/ESTHER trial in Cameroon. HIV.Med. available from: PM:24589279
80. Parienti, J.J., Massari, V., Reliquet, V., Chaillot, F., Le, M.G., Arvieux, C., Vabret, A., & Verdon, R. 2007. Effect of twice-daily nevirapine on adherence in HIV-1-infected patients: a randomized controlled study. AIDS, 21, (16) 2217-2222 available from: PM:18090049
81. Hill, A., McBride, A., Sawyer, A.W., Clumeck, N., & Gupta, R.K. 2013. Resistance at virological failure using boosted protease inhibitors versus nonnucleoside reverse transcriptase inhibitors as first-line antiretroviral therapy--implications for sustained efficacy of ART in resource-limited settings. J.Infect.Dis., 207 Suppl 2, S78-S84 available from: PM:23687293
82. Orrell, C., Harling, G., Lawn, S.D., Kaplan, R., McNally, M., Bekker, L.G., & Wood, R. 2007. Conservation of first-line antiretroviral treatment regimen where therapeutic options are limited. Antivir.Ther., 12, (1) 83-88 available from: PM:17503751
83. Rutstein SE, Hosseinipour MC, Kamwendo D, Soko A, Mkandawire M, Biddle AK, et al. (2015) Dried Blood Spots for Viral Load Monitoring in Malawi: Feasible and Effective. PLoS ONE 10(4): e0124748. doi:10.1371/journal.pone.0124748

84. Bonner, K., Mezochow, A., Roberts, T., Ford, N., & Cohn, J. 2013. Viral load monitoring as a tool to reinforce adherence: a systematic review. J.Acquir.Immune.Defic.Syndr., 64, (1) 74-78 available from: PM:23774877
85. Bärnighausen T, Chaiyachati K, Chimbindi N, et al. Interventions to increase antiretroviral adherence in sub-Saharan Africa: a systematic review of evaluation studies. Lancet Infect Dis 2011; 11: 942–51.
86. Kranzer, K. & Ford, N. Unstructured treatment interruption of antiretroviral therapy in clinical practice: a systematic review. Trop.Med.Int.Health, 2011; 16, (10) 1297-1313 available from: PM:21718394
87. Kranzer, K., Lewis, J.J., Ford, N., Zeinecker, J., Orrell, C., Lawn, S.D., Bekker, L.G., & Wood, R. 2010. Treatment interruption in a primary care antiretroviral therapy program in South Africa: cohort analysis of trends and risk factors. J.Acquir.Immune.Defic.Syndr., 55, (3) e17-e23 available from: PM:20827216
88. Tassie, J.M., Baijal, P., Vitoria, M.A., Alisalad, A., Crowley, S.P., & Souteyrand, Y. 2010. Trends in retention on antiretroviral therapy in national programs in low-income and middle-income countries. J.Acquir.Immune.Defic.Syndr., 54, (4) 437-441 available from: PM:20351559
89. Wandeler, G., Keiser, O., Pfeiffer, K., Pestilli, S., Fritz, C., Labhardt, N.D., Mbofana, F., Mudyiradima, R., Emmel, J., Egger, M., & Ehmer, J. 2012. Outcomes of antiretroviral treatment programs in rural Southern Africa. J.Acquir.Immune.Defic.Syndr., 59, (2) e9-16 available from: PM:22067665
90. Wallis, C.L., Mellors, J.W., Venter, W.D., Sanne, I., & Stevens, W. 2010. Varied patterns of HIV-1 drug resistance on failing first-line antiretroviral therapy in South Africa. J.Acquir.Immune.Defic.Syndr., 53, (4) 480-484 available from: PM:19801944
91. McMahon, J.H., Elliott, J.H., Bertagnolio, S., Kubiak, R., & Jordan, M.R. 2013. Viral suppression after 12 months of antiretroviral therapy in low- and middle-income countries: a systematic review. Bull.World Health Organ, 91, (5) 377-385E available from: PM:23678201
92. Charurat, M., Oyegunle, M., Benjamin, R., Habib, A., Eze, E., Ele, P., Ibanga, I., Ajayi, S., Eng, M., Mondal, P., Gebi, U., Iwu, E., Etiebet, M.A., Abimiku, A., Dakum, P., Farley, J., & Blattner, W. 2010. Patient retention and adherence to antiretrovirals in a large antiretroviral therapy program in Nigeria: a longitudinal analysis for risk factors. PLoS.One., 5, (5) e10584 available from: PM:20485670
93. DeGruttola, V., Dix, L., D'Aquila, R., Holder, D., Phillips, A., Ait-Khaled, M., Baxter, J., Clevenbergh, P., Hammer, S., Harrigan, R., Katzenstein, D., Lanier, R., Miller, M., Para, M., Yerly, S., Zolopa, A., Murray, J., Patick, A., Miller, V., Castillo, S., Pedneault, L., & Mellors, J. 2000. The relation between baseline HIV drug resistance and response to antiretroviral therapy: re-analysis of retrospective and prospective studies using a standardized data analysis plan. Antivir.Ther., 5, (1) 41-48 available from: PM:10846592
94. Grinsztejn, B., Nguyen, B.Y., Katlama, C., Gatell, J.M., Lazzarin, A., Vittecoq, D., Gonzalez, C.J., Chen, J., Harvey, C.M., & Isaacs, R.D. 2007. Safety and efficacy of the HIV-1 integrase inhibitor raltegravir (MK-0518) in treatment-experienced patients with multidrug-resistant virus: a phase II randomised controlled trial. Lancet, 369, (9569) 1261-1269 available from: PM:17434401
95. Rosenbloom, D.I., Hill, A.L., Rabi, S.A., Siliciano, R.F., & Nowak, M.A. 2012. Antiretroviral dynamics determines HIV evolution and predicts therapy outcome. Nat.Med., 18, (9) 1378-1385 available from: PM:22941277
96. Genberg, B.L., Wilson, I.B., Bangsberg, D.R., Arnsten, J., Goggin, K., Remien, R.H., Simoni, J., Gross, R., Reynolds, N., Rosen, M., & Liu, H. 2012. Patterns of antiretroviral therapy adherence and impact on HIV RNA among patients in North America. AIDS, 26, (11) 1415-1423 available from: PM:22767342

97. Cambiano, V., Lampe, F.C., Rodger, A.J., Smith, C.J., Geretti, A.M., Lodwick, R.K., Holloway, J., Johnson, M., & Phillips, A.N. 2010b. Use of a prescription-based measure of antiretroviral therapy adherence to predict viral rebound in HIV-infected individuals with viral suppression. HIV.Med., 11, (3) 216-224 available from: PM:20002781
98. Arnsten, J.H., Demas, P.A., Farzadegan, H., Grant, R.W., Gourevitch, M.N., Chang, C.J., Buono, D., Eckholdt, H., Howard, A.A., & Schoenbaum, E.E. 2001. Antiretroviral therapy adherence and viral suppression in HIV-infected drug users: comparison of self-report and electronic monitoring. Clin.Infect.Dis., 33, (8) 1417-1423 available from: PM:11550118
99. Montaner, J.S., Reiss, P., Cooper, D., Vella, S., Harris, M., Conway, B., Wainberg, M.A., Smith, D., Robinson, P., Hall, D., Myers, M., & Lange, J.M. A randomized, double-blind trial comparing combinations of nevirapine, didanosine, and zidovudine for HIV-infected patients: the INCAS Trial. Italy, The Netherlands, Canada and Australia Study. JAMA 1998, 279, (12) 930-937 available from: PM:9544767
100. Eron, J.J., Benoit, S.L., Jemsek, J., MacArthur, R.D., Santana, J., Quinn, J.B., Kuritzkes, D.R., Fallon, M.A., & Rubin, M. 1995. Treatment with lamivudine, zidovudine, or both in HIV-positive patients with 200 to 500 CD4+ cells per cubic millimeter. North American HIV Working Party. N.Engl.J.Med., 333, (25) 1662-1669 available from: PM:7477218
101. Havlir, D., McLaughlin, M.M., & Richman, D.D. 1995. A pilot study to evaluate the development of resistance to nevirapine in asymptomatic human immunodeficiency virus-infected patients with CD4 cell counts of > 500/mm3: AIDS Clinical Trials Group Protocol 208. J.Infect.Dis., 172, (5) 1379-1383 available from: PM:7594683
102. Kuritzkes, D.R., Quinn, J.B., Benoit, S.L., Shugarts, D.L., Griffin, A., Bakhtiari, M., Poticha, D., Eron, J.J., Fallon, M.A., & Rubin, M. 1996. Drug resistance and virologic response in NUCA 3001, a randomized trial of lamivudine (3TC) versus zidovudine (zidovudine) versus zidovudine plus 3TC in previously untreated patients. AIDS, 10, (9) 975-981 available from: PM:8853730
103. Larder, B.A. 1995. Viral resistance and the selection of antiretroviral combinations. J.Acquir.Immune.Defic.Syndr.Hum.Retrovirol., 10 Suppl 1, S28-S33 available from: PM:8595505
104. Phillips, A.N., Eron, J., Bartlett, J., Kuritzkes, D.R., Johnson, V.A., Gilbert, C., Johnson, J., Keller, A., & Hill, A.M. 1997. Correspondence between the effect of zidovudine plus lamivudine on plasma HIV level/CD4 lymphocyte count and the incidence of clinical disease in infected individuals. North American Lamivudine HIV Working Group. AIDS, 11, (2) 169-175 available from: PM:9030363
105. Wittkop, L., Gunthard, H.F., de, W.F., Dunn, D., Cozzi-Lepri, A., De, L.A., Kucherer, C., Obel, N., von, W., V, Masquelier, B., Stephan, C., Torti, C., Antinori, A., Garcia, F., Judd, A., Porter, K., Thiebaut, R., Castro, H., van Sighem, A.I., Colin, C., Kjaer, J., Lundgren, J.D., Paredes, R., Pozniak, A., Clotet, B., Phillips, A., Pillay, D., & Chene, G. 2011. Effect of transmitted drug resistance on virological and immunological response to initial combination antiretroviral therapy for HIV (EuroCoord-CHAIN joint project): a European multicohort study. Lancet Infect.Dis., 11, (5) 363-371 available from: PM:21354861
106. Wittkop, L., Bitard, J., Lazaro, E., Neau, D., Bonnet, F., Mercie, P., Dupon, M., Hessamfar, M., Ventura, M., Malvy, D., Dabis, F., Pellegrin, J.L., Moreau, J.F., Thiebaut, R., & Pellegrin, I. 2013. Effect of cytomegalovirus-induced immune response, self antigen-induced immune response, and microbial translocation on chronic immune activation in successfully treated HIV type 1-infected patients: the ANRS CO3 Aquitaine Cohort. J.Infect.Dis., 207, (4) 622-627 available from: PM:23204178
107. Gallant, J.E., Staszewski, S., Pozniak, A.L., DeJesus, E., Suleiman, J.M., Miller, M.D., Coakley, D.F., Lu, B., Toole, J.J., & Cheng, A.K. 2004. Efficacy and safety of tenofovir DF vs stavudine in combination therapy in antiretroviral-naive patients: a 3-year randomized trial. JAMA, 292, (2) 191-201 available from: PM:15249568

108. Harrigan, P.R., Hogg, R.S., Dong, W.W., Yip, B., Wynhoven, B., Woodward, J., Brumme, C.J., Brumme, Z.L., Mo, T., Alexander, C.S., & Montaner, J.S. 2005. Predictors of HIV drug-resistance mutations in a large antiretroviral-naive cohort initiating triple antiretroviral therapy. J.Infect.Dis., 191, (3) 339-347 available from: PM:1563309
109. Ledergerber, B., Egger, M., Opravil, M., Telenti, A., Hirschel, B., Battegay, M., Vernazza, P., Sudre, P., Flepp, M., Furrer, H., Francioli, P., & Weber, R. 1999. Clinical progression and virological failure on highly active antiretroviral therapy in HIV-1 patients: a prospective cohort study. Swiss HIV Cohort Study. Lancet, 353, (9156) 863-868 available from: PM:10093977
110. Phillips, A.N., Staszewski, S., Weber, R., Kirk, O., Francioli, P., Miller, V., Vernazza, P., Lundgren, J.D., & Ledergerber, B. 2001. HIV viral load response to antiretroviral therapy according to the baseline CD4 cell count and viral load. JAMA, 286, (20) 2560-2567 available from: PM:11722270
111. Phillips, A.N., Dunn, D., Sabin, C., Pozniak, A., Matthias, R., Geretti, A.M., Clarke, J., Churchill, D., Williams, I., Hill, T., Green, H., Porter, K., Scullard, G., Johnson, M., Easterbrook, P., Gilson, R., Fisher, M., Loveday, C., Gazzard, B., & Pillay, D. 2005. Long term probability of detection of HIV-1 drug resistance after starting antiretroviral therapy in routine clinical practice. AIDS, 19, (5) 487-494 available from: PM:15764854
112. Staszewski, S., Miller, V., Sabin, C., Carlebach, A., Berger, A.M., Weidmann, E., Helm, E.B., Hill, A., & Phillips, A. 1999a. Virological response to protease inhibitor therapy in an HIV clinic cohort. AIDS, 13, (3) 367-373 available from: PM:10199227
113. Staszewski, S., Miller, V., Sabin, C., Schlecht, C., Gute, P., Stamm, S., Leder, T., Berger, A., Weidemann, E., Hill, A., & Phillips, A. 1999b. Determinants of sustainable CD4 lymphocyte count increases in response to antiretroviral therapy. AIDS, 13, (8) 951-956 available from: PM:10371176
114. Van Leth, L.F., Phanuphak, P., Ruxrungtham, K., Baraldi, E., Miller, S., Gazzard, B., Cahn, P., Lalloo, U.G., van der Westhuizen, I.P., Malan, D.R., Johnson, M.A., Santos, B.R., Mulcahy, F., Wood, R., Levi, G.C., Reboredo, G., Squires, K., Cassetti, I., Petit, D., Raffi, F., Katlama, C., Murphy, R.L., Horban, A., Dam, J.P., Hassink, E., van, L.R., Robinson, P., Wit, F.W., & Lange, J.M. 2004. Comparison of first-line antiretroviral therapy with regimens including nevirapine, efavirenz, or both drugs, plus stavudine and lamivudine: a randomised open-label trial, the 2NN Study. Lancet, 363, (9417) 1253-1263 available from: PM:15094269
115. Cambiano V, Bertagnolio S, Jordan M, Pillay D, Perriens J, Venter F, et al. Predicted levels of HIV drug resistance: potential impact of expanding diagnosis, retention, and eligibility criteria for antiretroviral therapy initiation. AIDS 2014, 28 (Suppl 1):S15–S23.
116. Ruggiero A, Cozzi-Lepri A, Beloukas A, Richman D, Khoo S, Phillips AN, et al. ERAS Study Group. Factors Associated With Persistence of Plasma HIV-1 RNA During Long-term Continuously Suppressive Firstline Antiretroviral Therapy. Open Forum Infectious Diseases 2018, 5 https://doi.org/10.1093/ofid/ofy032
117. Gross, R., Bilker, W.B., Friedman, H.M., & Strom, B.L. 2001. Effect of adherence to newly initiated antiretroviral therapy on plasma viral load. AIDS, 15, (16) 2109-2117 available from: PM:11684930
118. Ledergerber, B., Lundgren, J.D., Walker, A.S., Sabin, C., Justice, A., Reiss, P., Mussini, C., Wit, F., d'Arminio, M.A., Weber, R., Fusco, G., Staszewski, S., Law, M., Hogg, R., Lampe, F., Gill, M.J., Castelli, F., & Phillips, A.N. 2004. Predictors of trend in CD4-positive T-cell count and mortality among HIV-1-infected individuals with virological failure to all three antiretroviral-drug classes. Lancet, 364, (9428) 51-62 available from: PM:15234856.
119. A Mocroft, A N Phillips, J Gatell, B Ledergerber, M Fisher, N Clumeck,et al. Normalisation of CD4 counts in patients with HIV-1 infection and maximum virological suppression who are taking combination antiretroviral therapy: an observational cohort study Lancet 2007; 370: 407–13 Lancet 2007; 370: 407–13

120. Bishop J, DeShields S, Cunningham T, Troy SB. CD4 Count Recovery After Initiation of Antiretroviral Therapy in Patients Infected With Human Immunodeficiency Virus. Am JmedSci 2016;352(3):239–244
121. Geng E, Neilands T, Thiebaut R, Bosco Bwana M, Nash D, Moore R, et al. CD4 T cell recovery during suppression of HIV replication: an international comparison of the immunological efficacy of antiretroviral therapy in North America, Asia and Africa. International Journal of Epidemiology, 2015, 251–263 doi: 10.1093/ije/dyu271
122. O'Connor J, Smith CJ, Lampe FC, Hill T, Gompels M, Hay P, et al. Failure to achieve a CD4 cell count response on combination antiretroviral therapy despite consistent viral load suppression. AIDS 2014, 28:919–924
123. d'Arminio Monforte A, Cozzi Lepri A, Phillips AN, et al. Interruption of HAART in HIV clinical practice. Results from the ICONA study. JAIDS 2005; 38: 407-416
124. Li X, Margolick JB, Conover CS, et al. Interruption and discontinuation of HART in the MACS. JAIDS 2005; 38: 3:320-328.
125. Mocroft A, Youle M, Moore A, et al. Reasons for modification and discontinuation of antiretrovirals: results from a single treatment centre. AIDS 2001; 15 (2): 185-194.
126. Wit FWNM, Blanckenberg DH, Brinkman K, et al. Safety of long-term interruption of successful antiretroviral therapy: the ATHENA cohort study. AIDS 2005; 19: 345-348
127. Sigaloff K, et a. Accumulation of HIV Drug Resistance Mutations in Patients Failing First-Line Antiretroviral Treatment in South Africa. AIDS Res Hum Retr 2012; 28:171-175.
128. Deeks SG, Grant RM, Wrin T, et al. Persistence of drug-resistant HIV-1 after a structured treatment interruption and its impact on treatment response. AIDS 2003; 17:361-370.
129. Devereux HL, Youle M, Johnson MA, et al Rapid decline in detectability of HIV-1 drug resistance mutations after stopping therapy. AIDS 1999; 13:F123-F127.
130. Devereux HL, Emery VC, Johnson MA, et al. Replicative fitness in vivo of HIV-1 variants with multiple drug resistance associated mutations. J Med Virol 2001:; 65:218-224.
131. Hance AJ, Lemiale V, Izopet J, et al. Changes in HIV-1 populations after treatment interruption in patients failing antiretroviral therapy. J Virol 2001; 75:6410-6417
132. Tarwater PM, Parish M, Gallant JE. Prolonged treatment interruption after immunologic response to HAART. Clin Infect Dis 2003; 37:1541-1548.
133. Walter H, Low P, Harrer T, et al. No evidence for persistence of multidrug resistant viral strains after a 7-month treatment interruption in an HIV-1 infected individual. JAIDS 2002; 31:137-146
134. Phillips A, CASCADE Collaboration. Short-term risk of AIDS according to current CD4 cell count and viral load in antiretroviral drug-naïve individuals and those treated in the monotherapy era. AIDS, 2004. 18(1):51-8.
135. UN. 2019 Revision of World Population Prospects https://population.un.org/wpp/
136. Vandepitte J, Lyerla J, Dallabetta G, Crabbe F, Alary M, Buve A. Estimates of the number of female sex workers in different regions of the world. Sex Transm Infect 2006;82(Suppl III):iii18–iii25. doi: 10.1136/sti.2006.020081
137. Cowan FM, Davey CB, Fearon E, Mushati P, Dirawo J, Cambiano V et al. The HIV Care Cascade Among Female Sex Workers in Zimbabwe: Results of a Population-Based Survey From the Sisters Antiretroviral Therapy Programme for Prevention of HIV, an Integrated Response (SAPPH-IRe) Trial. J Acquir Immune Defic Syndr 2017;74:375–382
138. Gregson, S., et al., HIV decline in Zimbabwe due to reductions in risky sex? Evidence from a comprehensive epidemiological review. International Journey Epidemiology, 2010. 39(5): p. 1311-23.
139. Halperin, D.T., et al., A surprising prevention success: Why did the HIV epidemic decline in Zimbabwe? PLoS Medicine, 2011. 8(2).
140. Mishra, Sharmistha Using mathematical models to characterize HIV epidemics for the design of HIV prevention strategies. https://spiral.imperial.ac.uk/handle/10044/1/24913

141. Masson L, Passmore J, Liebenberg LJ, Werner L, Baxter C, Arnold KB. Genital Inflammation and the Risk of HIV Acquisition in Women. Clinical Infectious Diseases 2015;61(2):260–9
142. Nicolosi, A., et al., The Efficiency of Male-to Female and Female-to-Male Sexual Transmission of the Human Immunodeficiency Virus: A Study of 730 Stable Couples. Epidemiology, 1994. 5(6).
143. Tan W, Chow E, Fairley CK., Chen M, Bradshaw C, Read TRH. Sensitivity of HIV rapid tests compared with fourth-generation enzyme immunoassays or HIV RNA tests AIDS: July 31, 2016 - Volume 30 - Issue 12 - p 1951-1960 doi: 10.1097/QAD.0000000000001134
144. Rosen S, Fox MP. Retention in HIV Care between Testing and Treatment in Sub-Saharan Africa: A Systematic Review. PLOS Medicine 2011; 8. Article Number: e1001056
145. Jain V, Sucupira MC, Bacchetti P, Hartogensis W, Diaz RS, Kallas EG, et al. Differential Persistence of Transmitted HIV-1 Drug Resistance Mutation Classes. J Infect Dis 2011; 203(8):1174-1181.
146. Yang W-L, Kouyos RD, Böni J, Yerly S, Klimkait T, Aubert V, et al. Persistence of Transmitted HIV-1 Drug Resistance Mutations Associated with Fitness Costs and Viral Genetic Backgrounds. PLoS Pathog 2015 11(3): e1004722. doi:10.1371/journal.ppat.1004722
147. Heestermans T, Browne JL, Aitken SC, et al. Determinants of adherence to antiretroviral therapy among HIV-positive adults in sub-Saharan Africa: a systematic review. BMJ Global Health 2016;1:e000125. doi:10.1136/bmjgh-2016-000125
148. McMahon JH, Spelman T, Ford N, Greig J, Mesic A, Ssonko C et al. Risk factors for unstructured treatment interruptions and association with survival in low to middle income countries. AIDS Res and Therapy 2016; 13. Article Number: 25
149. Agbaji OO, Abah IO, Falang KD, Ebonyi AO, Musa J, Ugoagwu P, et al. Treatment Discontinuation in Adult HIV-Infected Patients on First-Line Antiretroviral Therapy in Nigeria. Curr HIV Research 2015; 13: 184-192 DOI: 10.2174/1570162X1303150506181945
150. Tenores Study Group. Global epidemiology of drug resistance after failure of WHO recommended first-line regimens for adult HIV-1 infection: a multicentre retrospective cohort study. Lancet Infect Dis 2016. http://dx.doi.org/10.1016/S1473-3099(15)00536-8
151. Msyamboza KP, Ngwira B, Dzowela T, Mvula C, Kathyola D, et al. (2011) The Burden of Selected Chronic Non-Communicable Diseases and Their Risk Factors in Malawi: Nationwide STEPS Survey. PLoS ONE 6(5): e20316. doi:10.1371/journal.pone.0020316
152. Thorogood M, Connor M, Tollman S, Lewando Hundt G, Fowkes G, Marsh J. A cross-sectional study of vascular risk factors in a rural South African population: data from the Southern African Stroke Prevention Initiative (SASPI). BMC Public Health 2007, 7:326. doi:10.1186/1471-2458-7-326
153. Berrington de Gonzalez A, Hartge P, Cerhan JR, Flint AJ, Hannan L, MacInnis RJ. Body-Mass Index and Mortality among 1.46 Million White Adults. N Engl J Med. 2010 December 2; 363(23): 2211–2219. doi:10.1056/NEJMoa1000367.
154. Flegal KM, Kit BK, Orpana H, Graubard BI. Association of All-Cause Mortality With Overweight and Obesity Using Standard Body Mass Index Categories: A Systematic Review and Meta-analysis. JAMA. 2013 January 2; 309(1): 71–82. doi:10.1001/jama.2012.113905.
155. Achhra AC, Sabin CA, Ryom L, Hatleberg C, d'Aminio Monforte, de Wit S, et al. Body Mass Index and the Risk of Serious Non-AIDS Events and All-Cause Mortality in Treated HIV-Positive individuals: D:A:D Cohort Analysis. J Acquir Immune Defic Syndr _ Volume 78, Number 5, August 15, 2018.
156. Kivimäki M, Kuosma , Ferrie JF, Luukkonen R, Nyberg ST, Alfredsson L, et al. Overweight, obesity, and risk of cardiometabolic multimorbidity: pooled analysis of individual-level data for 120 813 adults from 16 cohort studies from the USA and Europe. Lancet Public Health 2017; 2: e277–85.
157. Kanters S, et al. Meta-analysis of association between baseline NNRTI resistance and virologic failure prepared for WHO guidelines meeting June 2019.

158. https://clinicalinfo.hiv.gov/en/guidelines/pediatric-arv/zidovudine
159. Cresswell J, Campbell OMR, De Silva MJ, Filippi V. Effect of maternal obesity on neonatal death in sub-Saharan Africa: multivariable analysis of 27 national datasets. Lancet 2012; 380: 1325–30
160. Cowan FM, Davey C, Fearon E, Mushati P, Dirawo J, Chabata S, et al. Targeted combination prevention to support female sex workers in Zimbabwe accessing and adhering to antiretrovirals for treatment and prevention of HIV (SAPPH-IRe): a cluster-randomised trial. Lancet HIV 2018 http://dx.doi.org/10.1016/S2352-3018(18)30111-5
161. Wilson D (2015) HIV Programs for Sex Workers: Lessons and Challenges for Developing and Delivering Programs. PLoS Med 12(6):e1001808. doi:10.1371/journal.pmed.1001808)
162. Salomon JA, Vos T, Hogan DR, et al. Common values in assessing health outcomes from disease and injury: disability weights measurement study for the Global Burden of Disease Study 2010. Lancet 2012; 380: 2129–43.
163. CHAI Market report 2020. clintonhealthaccess.org/hiv-mid-year-market-memo-2020/
164. Eaton J et al. Health benefits, costs, and cost-effectiveness of earlier eligibility for adult antiretroviral therapy and expanded treatment coverage: a combined analysis of 12 mathematical models. Lancet Global Health 2014: E23-E34
165. Hyle, E. P., Jani, I. V, Lehe, J., Su, A. E., Wood, R., Quevedo, J., … Walensky, R. P. (2014). The Clinical and Economic Impact of Point-of-Care CD4 Testing in Mozambique and Other Resource-Limited Settings: A Cost-Effectiveness Analysis. PLoS Med, 11(9), e1001725. doi:10.1371/journal.pmed.1001725.
166. Keebler D, Revill P, et al. How Should HIV Programmes Monitor Adults on ART? A Combined Analysis of Three Mathematical Models. Lancet Global Health 2014. E35-E43.
167. Global Fund Releases. http://www.theglobalfund.org/en/mediacenter/newsreleases/2015-06-10_New_Approach_on_HIV_Viral_Load_Testing/ http://www.theglobalfund.org/en/procurement/viral-load-early-infant-diagnostics/)
168. Siapka M, Remme M, Dayo Obure C, Maier C, Dehne KL, Vassall A. Is there scope for cost savings and efficiency gains in HIV services? A systematic review of the evidence from low- and middle-income countries. Bull World Health Organ 2014;92:499–511AD doi:http://dx.doi.org/10.2471/BLT.13.127639;
169. Tagar E, Sundaram M, Condliffe K, Matatiyo B, Chimbwandira F, et al. Multi-Country Analysis of Treatment Costs for HIV/AIDS (MATCH): Facility-Level ART Unit Cost Analysis in Ethiopia, Malawi, Rwanda, South Africa and Zambia. PLoS ONE 2014; 9(11): e108304. doi:10.1371/journal.pone.0108304;
170. Menzies NA, Berruti AA, Blandford JM (2012) The Determinants of HIV Treatment Costs in Resource Limited Settings. PLoS ONE 7(11): e48726. doi:10.1371/journal.pone.0048726
171. Jamieson L, Gomez GB, Rebed K, Brown B, Subedar H, Jenkins S, et al. The impact of self-selection based on HIV risk on the cost-effectiveness of preexposure prophylaxis in South Africa. AIDS 2020, 34:883–891
172. Pretorius C, Schnure M, Dent J, Glaubius R, Mahiane G, Hamilton M et al. Modelling impact and cost-effectiveness of oral pre-exposure prophylaxis in 13 low-resource countries. J Int AIDS Soc 2020 e25451 DOI: 10.1002/jia2.25451
173. Roberts DA, Barnabas RV, Abuna F, Lagat H, Kinuthia J, Pintye J et al. The role of costing in the introduction and scale-up of HIV pre-exposure prophylaxis: evidence from integrating PrEP into routine maternal and child health and family planning clinics in western Kenya. J Int AIDS Soc 2019. e25296 DOI: 10.1002/jia2.25296.
